# Molecular profiling of pediatric and young adult colorectal cancer reveals a distinct genomic landscapes and potential therapeutic avenues

**DOI:** 10.1038/s41598-024-64149-7

**Published:** 2024-06-07

**Authors:** A. Busico, P. Gasparini, E. Rausa, L. Cattaneo, F. Bozzi, M. Silvestri, I. Capone, E. Conca, E. Tamborini, F. Perrone, M. Vitellaro, M.T. Ricci, M. Casanova, S. Chiaravalli, L. Bergamaschi, M. Massimino, M. Milione, G. Sozzi, G. Pruneri, A. Ferrari, S. Signoroni

**Affiliations:** 1 Department of Diagnostic Innovation, Pathology Unit 2, Fondazione IRCCS Istituto Nazionale Dei Tumori Di Milano, Milano, Italy; 2 sc Epigenomics and Biomarkers of Solid Tumors, Fondazione IRCCS Istituto Nazionale Dei Tumori Di Milano, Milano, Italy; 3 Unit of Hereditary Digestive Tract Tumors, Fondazione IRCCS Istituto Nazionale Dei Tumori Di Milano, Milano, Italy; 4 Department of Diagnostic Innovation, Biobank, Fondazione IRCCS Istituto Nazionale Dei Tumori Di Milano, Milano, Italy; 5 Colorectal Surgery Unit, Fondazione IRCCS Istituto Nazionale Dei Tumori Di Milano, Milano, Italy; 6 sc Pediatric Oncology, Fondazione IRCCS Istituto Nazionale Dei Tumori Di Milano, Milano, Italy; 7 Department of Diagnostic Innovation, Pathology Unit 1, Fondazione IRCCS Istituto Nazionale Dei Tumori Di Milano, Milano, Italy

**Keywords:** Cancer genomics, Paediatric cancer, Colorectal cancer

## Abstract

Colorectal cancer (CRC) is a global health concern, and the incidence of early onset (EO) CRC, has an upward trend. This study delves into the genomic landscape of EO-CRC, specifically focusing on pediatric (PED) and young adult (YA) patients, comparing them with adult (AD) CRC. In this retrospective monocentric investigation, we performed targeted next-generation sequencing to compare the mutational profile of 38 EO-CRCs patients (eight PED and 30 YA) to those of a ‘control group’ consisting of 56 AD-CRCs. Our findings reveal distinct molecular profiles in EO-CRC, notably in the WNT and PI3K-AKT pathways. In pediatrics, we observed a significantly higher frequency of RNF43 mutations, whereas APC mutations were more prevalent in adult cases. These observations suggest age-related differences in the activation of the WNT pathway. Pathway and copy number variation analysis reveal that AD-CRC and YA-CRC have more similarities than the pediatric patients. PED shows a peculiar profile with CDK6 amplification and the enrichment of lysine degradation pathway. These findings may open doors for personalized therapies, such as PI3K-AKT pathway inhibitors or CDK6 inhibitors for pediatric patients. Additionally, the distinct molecular signatures of EO-CRC underscore the need for age-specific treatment strategies and precision medicine. This study emphasizes the importance of comprehensive molecular investigations in EO-CRCs, which can potentially improve diagnostic accuracy, prognosis, and therapeutic decisions for these patients. Collaboration between the pediatric and adult oncology community is fundamental to improve oncological outcomes for this rare and challenging pediatric tumor.

## Introduction

Colorectal cancer (CRC) is the third most common cancer worldwide and the second cause of cancer-related deaths^1^. Despite the great majority of CRCs are sporadic (sCRC), they can also arise as secondary to predisposing genetic syndromes such as Lynch syndrome (LS) and familial adenomatous polyposis (FAP)^2^. While the incidence of colorectal cancer (CRC) has remained stable for years, it has been observed increasing in the population under the age of 40 years (early onset, EO-CRC)^3^. During the last two decades, tremendous improvements CRC treatment have been achieved, in particular in patients with a late onset CRC (over 50 years of age)^4^, mostly based on strict screening programs and an increasing understanding of the CRCs’ underlying molecular biology. On the other hand, the same cannot be said for EO-CRC, in particular PED-CRC in which a proper treatment strategy is not outlined and physicians tend to apply adult-CRC (AD-CRC) protocols^5,6^. In the pediatric age (PED: 0–19 years), onset of CRC is extremely rare, accounting for less than 0.1%^7^.

Plenty of evidence is available regarding AD-CRCs biology showing that they are an heterogeneous group of molecular diseases categorized by genomic and epigenetic alterations. Also, some studies highlighted the biological and clinical asset between EO-CRC and AD-CRC, demonstrating that EO-CRC exhibits a distinct molecular profile with a significant decrease in the prevalence of APC and WNT pathway mutations^7,8^. However, very few molecular studies are dedicated uniquely to pediatric CRC, as these young patients are usually included in early onset group^9,10^. A better comprehension of the molecular mechanisms behind tumorigenesis of PED-CRCs is crucial to design a specific treatment strategy in this rare disease the clinical management of which remains a challenge and currently no validated screening protocols are available.

To this, taking advantage of high-throughput technologies providing hundreds of gene mutations, copy number variations, microsatellite instability and mutational tumor burden, we here described and compared the genomic mutational landscape of a retrospective EO-CRC cohort of sporadic PED and young adults (YA) with AD-CRC patients. This comparison aims to enhance our understanding of this rare disease and identify more effective therapeutic pathways and improve the clinical management of this subset of PED and YA-CRC.

## Results

### Clinicopathological features of the studied cohort of CRCs

The retrospective clinical series analyzed consisted of 38 sporadic EO-CRCs tumor specimens, including eight PED-CRC (ranging from 9 to 19; mean age 14.9 years) and 30 YA-CRC (20–39 years; mean age 32.7 years). The results obtained in these two groups were compared to those of a ‘control group’ consisting of 56 AD-CRC (> 60 years, mean age 70.6 years). Clinical-pathological descriptions of all the studied clinical series are summarized in Table 1.

**Table 1:** The clinicopathological features of enrolled patients.

Features	Age		
	≤ 19 (%)	20–39 (%)	≥ 60 (%)
N of patients	8	30	56
Sex			
Male	5 (63)	16 (53)	32 (57)
Female	3 (37)	14 (47)	24 (43)
Median age	14.9	32.7	70.6
Primary tumor location			
Right-side colon	3 (38)	4 (13)	21 (38)
Left-side colon	4 (50)	15 (50)	18 (32)
Rectal	1 (12)	10 (33)	12 (21)
NA	0	1 (4)	5 (9)
Hystological type			
Adenocarcinoma NOS	4 (50)	26 (87)	54 (96)
Mucinous adenocarcinoma	2 (25)	3 (10)	1 (2)
Signet-ring cell carcinoma	2 (25)	1 (3)	1 (2)
Pathological stage			
I	0	2 (7)	1 (2)
II	1 (12)	4 (13)	11 (20)
III	3 (38)	12 (40)	16 (29)
IV	4 (50)	10 (33)	22 (39)
NA	0	2 (7)	6 (11)
MS STATUS			
MSS	6 (75)	28 (93)	47 (84)
L-MSI	0	0	3 (5)
H-MSI	2 (25)	2 (7)	6 (11)

*MSS* microsatellite stability, *L-MSI* low microsatellite instability, *H-MSI* high microsatellite instability.

A majority of PED-CRC were adenocarcinoma NOS (50%), while two (25%) and two (25%) were diagnosed with mucinous and signet ring cell carcinoma, respectively. Regarding the 30 YA-CRC, 26 (87%) were adenocarcinoma NOS, while one signet-ring cell subtype (3%) and three mucinous adenocarcinoma (10%). Pathological stage and morphological features characterizing CRCs is similar regardless ages of the patients.

### Genomic landscape overview of targeted mutational profile in CRC

**Figure 1:**
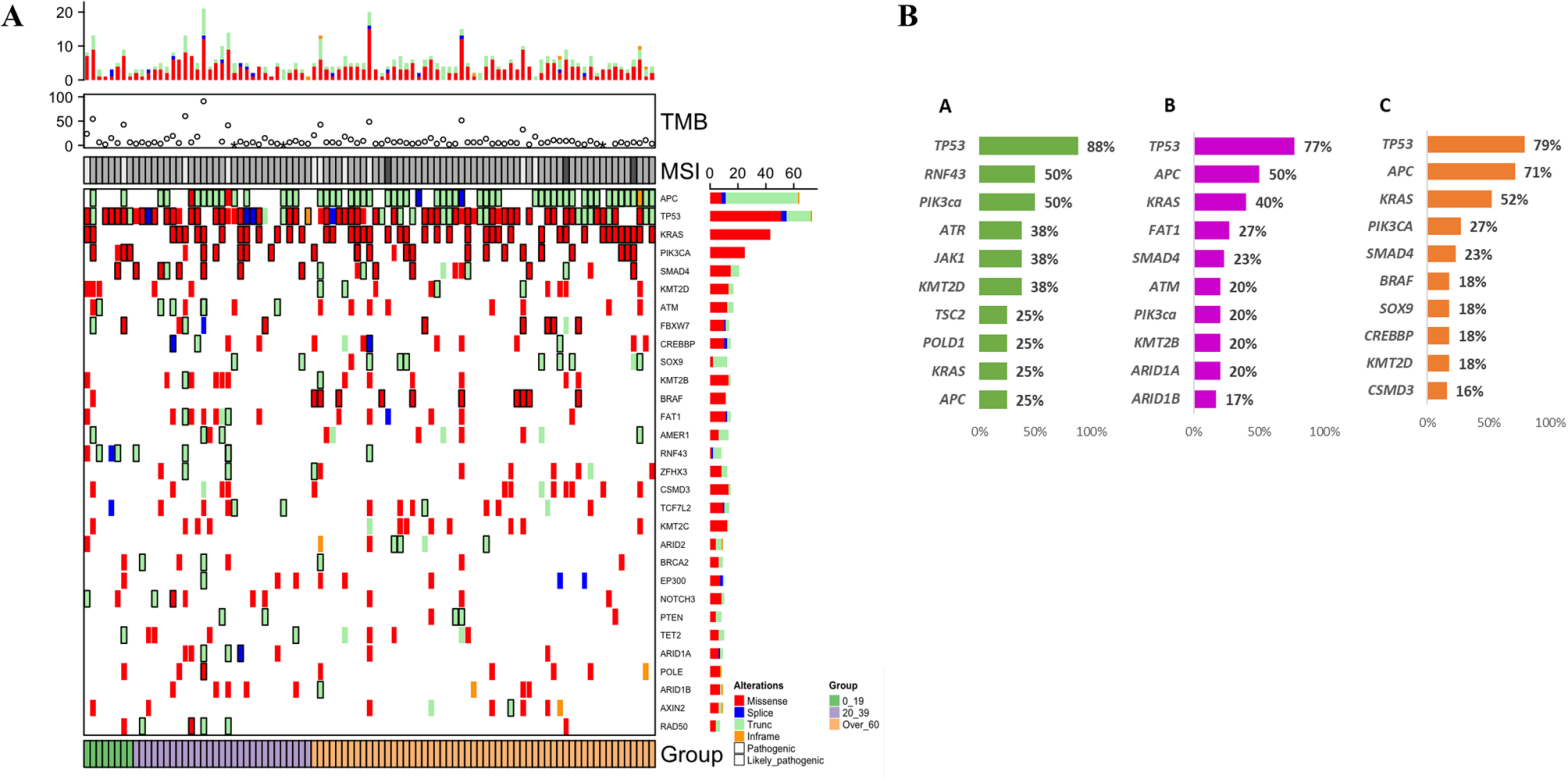
(**A**) Heatmap of top 30 altered genes in PED group, color green on the left (0–19 years); YA group, color purple in the middle (20–39 years) and AD group, color orange on the right (over 60 years). Information related to TMB and MSI (MSS, grey; H-MSI, white; L-MSI dark grey) were reported on the top while patient group association on the bottom. The types of alterations were indicated by different colors. Each column represented one patient. (**B**) Top 10 altered genes in PED-CRC (green, A), YA-CRC (purple, B) AD-CRC (orange, C).

A heat map of the top 30 altered genes of PED-CRC, YA-CRC and AD-CRC is illustrated in Fig. 1A. In more details, frequencies of the top 10 most altered genes among CRC of different age group (Fig. 1B) show how, with the exception of TP53, which is equally mutated in all groups, PED-CRCs have distinct frequencies and mutated genes than YA-and AD. Notably, in PED-CRCs, RNF43 (50%) and PIK3ca (50%) result to be frequently altered, followed by ATR, JAK1, KMT2D (38%), TSC2, KRAS, POLD1 and APC (25%). Among YA-CRC, APC (50%) was the most frequently mutated gene followed by KRAS (40%), FAT1 (27%), SMAD4 (23%), ATM, PIK3ca, KMT2B, ARID1A (20%) and ARID1B (17%). APC was observed to be mutated in 71% of AD-CRC, followed by KRAS (52%), PIK3ca (27%), SMAD4 (23%), BRAF, SOX9, CREBBP, KMT2D (18%) and CSMD3 (16%) (Fig. 1B).

H-MSI was observed in two (25%) PED, two (7%) YA and five (9%) AD-CRC.

Tumor mutational burden (TMB) was calculated for all samples, revealing a higher TMB (TMB-H) for PED-CRC compared to the other two groups. Specifically, PED-CRC showed a median of 20 mutations/Mb respect to YA- and AD-CRC where a median of 13.4 and 10.5 mutations/Mb was observed, respectively. PED-CRC were enriched with TMB-H patients (50%) compared to 20% in YA- and 30% in AD-CRC. TMB-H was associated with H-MSI in two PED, two YA, and six AD-CRC samples respectively (18.95–60.45 mutations/Mb), as well with a pathogenic mutation in POLE in a YA-CRC (91.22 mutation/Mb). Mutations in MSH2 and MLH1 DNA mismatch repair genes were detected in one PED and two in YA-CRC, while MSH2 and MSH6 single-copy gene loss was identified in one PED-CRC. Interestingly, three cases with H-MSI (two YA and one AD) also presented the variant mutation RNF43 p.Gly659ValfsTer41.

### EO-CRC showed different mutations respect to AD-CRC samples

A mutation frequency comparison between EO-CRC and AD-CRC samples was performed using the Fisher exact test. EO-CRC samples were characterized by the increased frequency of RNF43 (a WNT suppressor gene), TPTE (a PTEN-related tyrosine phosphatase) and RAD50 as well by a decrease of APC and BRAF mutations (Fisher test, p value < 0.05) (Table 2).

**Table 2:** Genes significantly enriched with groupwise comparison: EO-CRC vs AD-CRC (Fisher exact test, p-value < 0.05).

Gene	EO-CRC	AD-CRC	n_mutated_EO-CRC	n_mutated_AD-CRC	p-value
RNF43	0–19 + 20–39	Over 60	7 of 38	1 of 56	< 0.05
APC	0–19 + 20–39	Over 60	16 of 38	40 of 56	< 0.05
TPTE	0–19 + 20–39	Over 60	6 of 38	0 of 56	< 0.05
BRAF	0–19 + 20–39	Over 60	1 of 38	10 of 56	< 0.05
RAD50	0–19 + 20–39	Over_60	5 of 38	1 of 56	< 0.05

**Figure 2:**
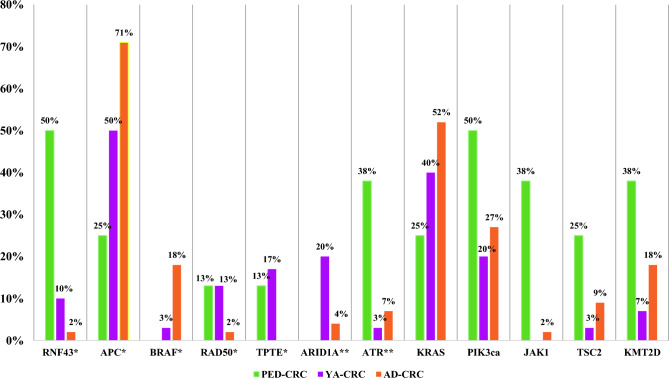
Distribution of genes enriched among the cohorts. Genes with* were significantly enriched (*Fisher exact test, p < 0.050; ** Fisher exact test, fdr < 0.050).

In order to discover significant differences and set priorities among PED-CRC, YA-CRC (EO-CRC) and AD-CRC, further analyses were performed comparing groups “in pairs”, and a distribution of genes enriched among the cohorts is represented in Fig. 2. A significant enrichment of RNF43 mutations observed in EO-CRC samples was maintained only by the PED-CRC respect to the YA-CRC and AD-CRC (fdr < 0.05) (Table 3). In these patients, mutations were enriched at RNF43 N-terminal as illustrated in Supplementary Fig. 1. At variance, APC was confirmed to be significantly less frequently mutated (fdr < 0.05) in both pediatric and young when compared with AD-CRC samples. In addition, our evidence showed that APC and RNF43 mutations were mutually exclusive in all but two samples (one YA- and one AD-). Interestingly, these two CRCs with both mutations showed H-MSI and the RNF43 p.Gly659ValfsTer41 variant that was previously reported to be enriched in H-MSI cancers. Also, the TPTE, enrichment observed in EO-CRC, defined YA-CRC (p.value < 0.05) but not PED-CRC.

**Table 3:** Genes significantly enriched with paired comparison (0_19 vs 20_39; 0_19 vs over_60; 20_39 vs over_60 (Fisher exact test, fdr < 0.05).

Gene	Group1	Group2	n_mutated_group1	n_mutated_group2	fdr
RNF43	0_19	Over_60	4 of 8	1 of 56	≤ 0.05
RNF43	0_19	20_39	4 of 8	3 of 30	≤ 0.05
ATR	0_19	20_39	3 of 8	1 of 30	≤ 0.05
ATR	0_19	Over_60	3 of 8	4 of 56	≤ 0.05
TPTE	20_39	Over_60	5 of 30	0 of 56	≤ 0.05
BRAF	20_39	Over_60	1 of 30	10 of 56	≤ 0.05
APC	0_19	Over_60	2 of 8	40 of 56	≤ 0.05
APC	20_39	Over_60	14 of 30	40 of 56	≤ 0.05
ARID1A	20_39	Over_60	6 of 30	2 of 56	≤ 0.05

BRAF^V600^ was confirmed to be significantly less mutated in YA- with respect to AD-CRC sample.

When compared “in pairs”, the RAD50 mutation enrichment observed in EO-CRC samples lost significance. Furthermore, by pairs comparison, two further genes emerged as differentially mutated: ATR in PED-CRC, and ARID1A in YA-CRC. While not statistically significant, KRAS mutation frequency increased accordingly with age: 25% in PED-, 40% in YA- and 52% in AD-CRC. Finally, PED-CRC also had an increased prevalence of PIK3ca, TSC2 and KMT2D, mutations, although not statistically significant (Fig. 2).

Overall, these results showed that pediatric was exclusively characterized by higher RNF43 and ATR mutations frequencies than young and adults. On the other hand, YA-CRC patients exhibited mutations in TPTE and ARID1A genes, while the reduction of APC and BRAF^V600^ mutations compared with AD CRC was observed in both PED and YA cases.

### Pathway analysis reveals a different WNT activation in PED-CRC specimen

**Figure 3:**
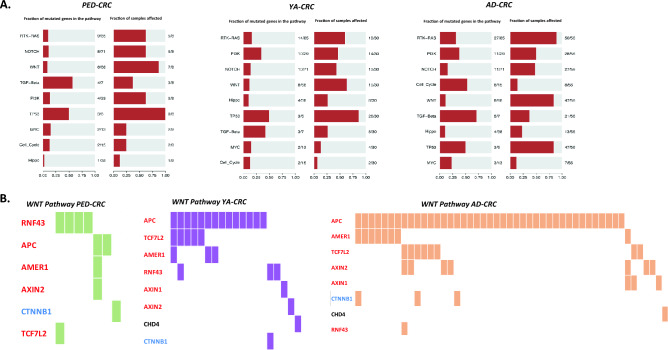
Oncogenic pathway enriched in PED (left), YA (middle) and AD-CRCs (right). The pathways analyzed are: (1) cell cycle, (2) Hippo signaling, (3) Myc signaling, (4) Notch signaling, (5) oxidative stress response/NRF2, (6) PI-3-Kinase signaling, (7) receptor-tyrosine kinase (RTK)/RAS/MAP-Kinase signaling, (8) TGFβ signaling, (9) P53 and (10) β-catenin/WNT signaling. (**A**) Barplots on the left show the fraction of mutated genes in the pathway; panels on the right show the fraction of samples with mutated genes in the pathway. (**B**) Oncoplot panels show the variants in WNT pathway in each group.

In order to infer which pathways were activated in EO-CRC and with what incidence, the mutational profiles of each group were interrogated using an ad hoc function implemented in Maftools R package^11^ (see Sect “Materials and methods” for details) (Fig. 3). As expected, TP53 and WNT pathways were found to be most frequently mutated in CRCs, regardless of age (Fig. 3a). Furthermore, PED-CRC samples were enriched in PI3K (62%) and NOTCH (62%) pathway mutations compared with YA- and AD-CRC. By contrast, RAS pathway genes showed higher alteration rates in AD-CRC (89%) compared to both PED (62%) and YA-CRC (60%) (Fig. 3a).

Interestingly, the activation of the WNT pathway resulted mainly driven by different mutated-genes according to age groups: (i) RNF43 (50%) and APC (25%) in PED-CRC, (ii) APC (50%), TCF7L2 (17%), AMER1 and RNF43 (10%) in YA, (iii) almost exclusively APC mutations (71%) and TCF7L2 and AMER1 (17%) in AD-CRC. (Fig. 3b).

**Figure 4:**
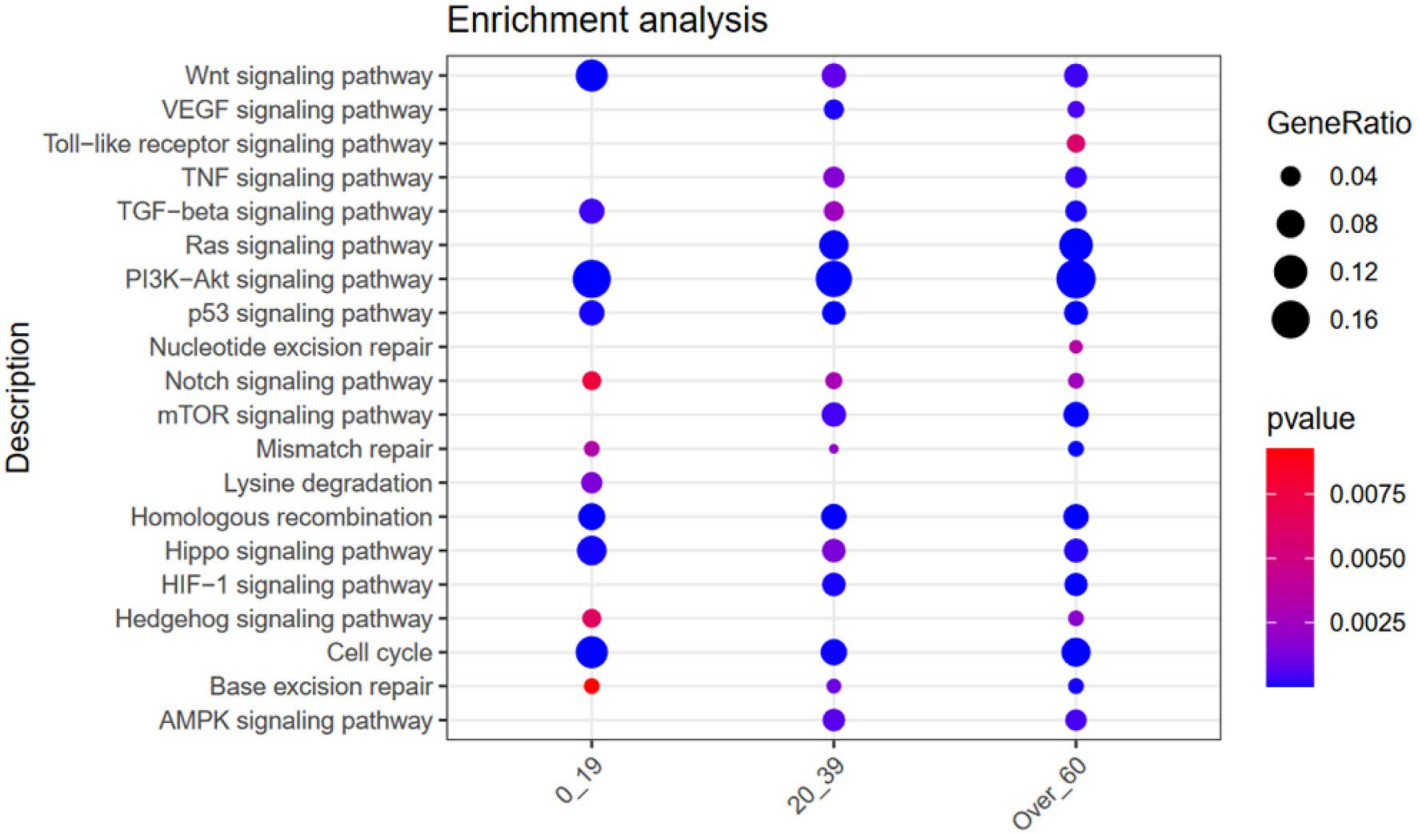
Pathway enrichment analysis using KEGG database. Significantly altered signaling pathways obtained from KEGG database and the three groups of patients characterizing the cohort are reported on y-axis and x-axis respectively. For each group, the fraction of altered genes composing the pathway (GeneRatio) is reported as dot where the size represents the magnitude of the ratio. The level of significance of each pathway within the groups is reported as color scale (blue = higher significance; red = lower significance).

Pathway enrichment analysis interrogating KEGG database was utilized to have a global overview all the pathways involved (Fig. 4), other than the 10 evaluated by Maftool. Again, PED-CRC revealed a distinct pathway signature different from YA- and AD-CRC. Notably, the lysine degradation pathway, involving epigenetic regulator genes such as SETD2, KMT2A, KMT2C, NSD2, and KMT2D, was only reported to be mutated in PED-CRC. PED-CRC also differentiated from the other age group for enrichment of WNT, Mismatch repair, Hippo signaling, and Hedgehog. On the other hand, PED-CRC is lacking activation of VEGF, TLR, TNF, RAS, Nucleotide excision, mTOR, HIF-1 and AMPK signalling pathways, all present in YA- and AD-CRC. The PI3K-AKT, TP53 and Homologous recombination pathways were similarly enriched in all CRC.

### Overview of targeted CNVs

**Figure 5:**
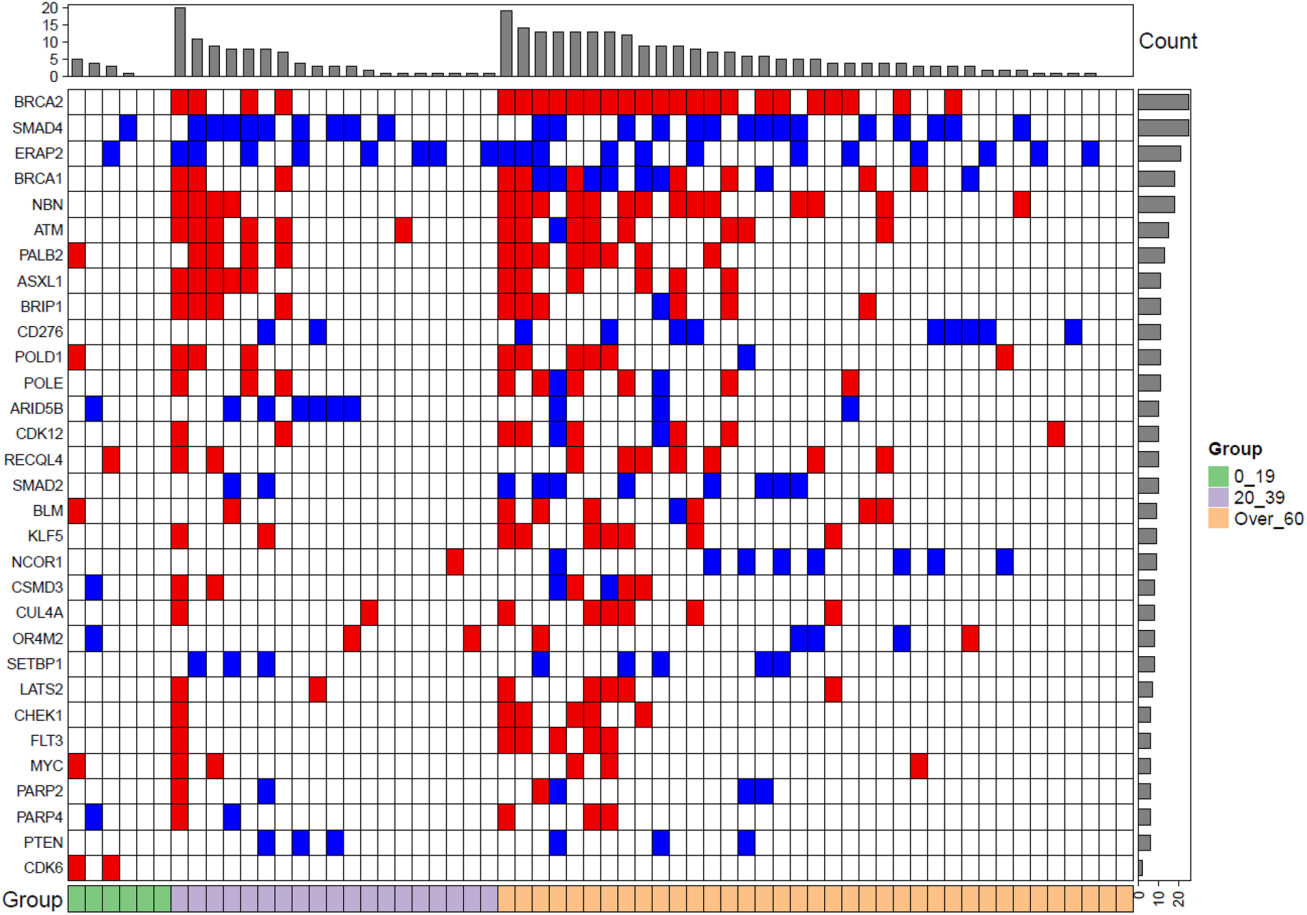
Copy number variation analysis. Heatmap of top 31 altered genes in PED-CRC, color green on the left (0–19 years); YA-CRC, color purple in the middle (20–39 years) and AD-CRC, color orange on the right (over 60 years). Red and blue colors refer to amplification and deletion events respectively. Each column represented one patient.

As CNVs play a crucial role in tumorigenesis, we assessed copy number alterations in all our studied cohorts by utilizing Ion reporter software. We successfully obtained results from most of our samples: 6/8 PED-, 19/30 YA- and 47/56 AD-CRC. Overall, a total of 33 copy gains and losses were detected in PED-CRC (median 5.5; range 3–10), 233 in YA-CRC (median 12.3; range 0–62) and 664 in AD patients (median 14.1, range 0–111). In PED-CRC, CDK6 gain (33%) was the most common amplification, absent in the other two groups. Copy number gains among YA and AD-CRC were observed in ATM, NBN, BRCA2, PALB2, ASXL1 and BRIP1, while SMAD4 and ERAP2 resulted as bi-allelic losses (Fig. 5). Overall, the AD-CRC and YA-CRC have more similarities in genes involved in CNVs than the pediatric ones, characterized by a much lower CNVs (Fig. 5).

## Discussion

Sporadic early-onset CRCs are extremely rare and their clinical management remains a challenge. Currently, no validated screening protocols are available for early-onset CRCs; this, in addition to more aggressive biological behaviour of EO-CRCs compared to AD-CRCs, often results in an advanced disease stage at diagnosis. A better comprehension of the mechanisms behind tumorigenesis of EO-CRCs is crucial to design a specific treatment strategy and ultimately improve long-term oncological outcomes.

We investigated the genomic mutational landscape of EO-CRCs and demonstrated that pediatric CRCs are characterized by a distinct molecular profile when compared to adult CRCs. We showed that WNT signalling and PI3K-AKT are the most affected pathways involved in PED-CRCs. Our evidence showed that WNT signaling pathway in PED-CRCs was mostly activated by RNF43 rather than APC mutations, that WNT activating mutations of APC and RNF43 are mutually exclusive, and that their frequency seems to be strictly related to the age. Indeed, RNF43 mutations are more frequent among pediatrics’ while APC among adults. Moreover, the only two cases showing both mutations observed in YA- and AD- had a different variant in a C-terminal truncating mutations (p.Gly659ValfsTer41) rather than an N-terminal one and were associated with a high MSI. This variant was reported to be enriched in H-MSI cancers and previous articles showed that the C-terminal truncating mutations, (like G659fs), do not confer WNT dependency onto CRC cells^12–14^. Katoh et al. showed that *APC* mutations usually sustain the vast majority of sporadic AD-CRCs by leading to constitutive activation of the canonical WNT/β catenin pathway, while RNF43 mutations can activate both canonical and non-canonical WNT signalling that do not require APC for execution^15^. Altogether these results highlight that the specific transcriptional programs mediated by non-canonical WNT pathways are important biological difference between pediatric and adult CRC and might allow personalized approaches for their treatment.

Another work by Elez et al. reported showed the significance of RNF43 mutations, which are implicated in 29% of MSS-mCRCBRAF-V600E tumors, in predicting treatment response and clinical outcomes (REF). Their findings underscore the potential of incorporating RNF43 mutations as routine biomarkers, aiding in treatment sequencing decisions for MSS-mCRCBRAF-V600E patients, and highlight a significant interplay between MAPK and RNF43-WNT pathways in the efficacy of targeted therapies. We concur with Elez et al.’s proposition of RNF43 as a routine biomarker, extending its relevance beyond MSS-mCRCBRAF-V600E to pediatric CRC. This underscores the multifaceted role of RNF43 in CRC, potentially serving as a therapeutic target and guiding clinical management in pediatrics.

Interestingly, Li et al. observed a frequent *RNF43* mutation contributing to a moderate activation of WNT signalling pathways in a subset of colorectal signet-ring cell carcinoma^16^. Similarly, in the present pediatric cohort, two RNF43-mutated samples were characterized by a signet-ring cell histology, and aggressive clinical behavior. Furthermore it has also been demonstrated that cell lines bearing RNF43 loss of function mutations were efficiently inhibited by porcupine inhibitors^17^. These functional results support the notion that the high frequency of RNF43 mutations in PED-CRCs could be a pivotal factor sustaining a more aggressive behavior characterized by signet ring and mucinous features, and suggest that specific upstream WNT inhibitors can be included in personalized therapeutic regimens.

Another work by Elez et al. showed the significance of RNF43 mutations, which are implicated in 29% of MSS-mCRC^BRAF-V600E^ tumors, in predicting treatment response and clinical outcomes^18^. Their findings underscore the potential of incorporating RNF43 mutations as routine biomarkers, aiding in treatment sequencing decisions for MSS-mCRC^BRAF-V600E^ patients, and highlight a significant interplay between MAPK and RNF43-WNT pathways in the efficacy of targeted therapies. We concur with Elez et al.’s proposition of RNF43 as a routine biomarker, extending its relevance beyond MSS-mCRC^BRAF-V600E^ to pediatric CRC. This underscores the multifaceted role of RNF43 in CRC, potentially serving as a therapeutic target and guiding clinical management in pediatrics.

Recently, authors have shown that the deregulation of PI3K-AKT signaling pathway drives cell survival, cell cycle progression and cellular growth in different malignancies^19^. In our pediatric group, PIK3ca was observed to be the second most aberrant gene with RNF43, compared to YA- and AD- that display a lower frequencies of PIK3ca-mutated cases. The PI3K/AKT pathway has been identified as deregulated in EO-CRC^20^. Our observations suggest that this pathway may be a predominant tumor progression driver in the pediatric age and for this reason, could have great potential as a target for inhibition. In the current clinical practice of pediatrics, a number of agents, such as rapamycin analogue sirolimus, were used to target this pathway with promising results in hematologic malignancies^21^. Based on this rationale, further ongoing trials are investigating the potential of a variety of PI3K/AKT/mTOR pathway inhibitors, alone or in combination, in pediatric solid tumors^22^. In addition to the most common mutations, we identified alterations within DNA repair genes, such as RAD50 and ATR, in particular in EO-CRCs rather than AD-CRCs. In this regard, defects in the DNA repair genes may promote tumorigenesis among younger patients^23^.

Ultimately, the epigenetic pathways and the disordered cell cycle regulation are known to be additional mechanisms for the development of malignancy in many pediatric patients^24^. First, we showed that the lysine degradation pathway was consistently more enriched among pediatrics when compared to the other groups. Particularly, SETD2, KMT2A, KMT2C, NSD2, and KMT2D, which encode histone lysine methyltransferases, appeared to be the most altered genes in this pathway. Over the last decades, the role of histone lysine methylation in chromatin regulation and tumors has been extensively investigated^25^. According to this understanding, new ways to chemically or biologically modulate other histone KMTs, such as NSD2, may be available in the next future in clinical practice.

Besides, we investigated the incidence of cyclin-dependent kinase 6 (CDK6) amplifications among the three cohorts and we found that it was that was exclusively present in PED group. The relevance of CDKs in promoting cancer initiation and progression made them a target for pharmacological inhibition for the scientific community. Recently, CDK4/6 kinase inhibitors such as palbociclib, ribociclib, and abemaciclib have been approved for clinical use also for pediatric patients, suggesting that a subgroup of PED-CRC with CDK6 amplification could benefit from these inhibitors^26–28^.

Notably, our evidence shows that specific pathways and CNVs are strictly linked with age. Particularly, we observed AMPK, RAS, mTOR, TNF signalling and SMAD4 deletion were significantly more frequent in YA- and AD-CRCs. Nonetheless, we observed 50% of pediatric patients with high TMB and 25% with H-MSI indicating the possibility of these patient to benefit to benefit from immune checkpoint inhibitors, potentially improving the poor prognosis^29^.

According to the aforementioned results, pediatric CRCs display unique histological and molecular characteristics driving carcinogenesis when compared to YA- and AD-CRCs. Validation of our findings (data not shown) with public database (TCGA and cbioportal.org) resulted in a similar trend of mutated genes and CNV within YA and AD. Nonetheless, public datasets lack sufficient data on PED-CRC, making it challenging to corroborate our observations. To this extent, our case series accurately delineates the genomic alteration differences among age groups.

Precision medicine could play an important role for PED-CRC now that the molecular profile is more clear and should be taken in consideration in clinical practice in order to optimize the oncological outcomes. Being an extremely rare pediatric tumor, this can be achieved only through the implementation of a broad international cooperation, such The European Cooperative Study Group for Pediatric Rare Tumors (EXPeRT) project^30^.

In conclusion, our molecular characterization of pediatric patients appears to have distinct clinical characteristics and crucial molecular pathways involved, implying that further investigation of this subgroup have the potential to improve diagnosis, prognosis, and therapeutic decisions for these patients, whose survival and clinical management remains challenging.

## Materials and methods

### Patient cohort

Our study is a retrospective monocentric analysis of a cohort of consecutive CRC cases diagnosed and treated between the years 2002–2020 at the Fondazione IRCCS Istituto Nazionale dei Tumori (INT), Milan Italy.

Formalin fixed paraffin embedded (FFPE) tissues of EO-CRC were selected and retrieved from the archives of the department of diagnostic pathology and from pediatric oncology unit of our Institute. All cases have been reviewed and reclassified by the internal pathologist according to the latest edition of WHO classification of tumours—5th edition, digestive system tumours, 2019^31^. For all patients, a colorectal polyposis was endoscopically excluded and a negative family history for CRC/polyps was assessed. Additionally, in order to further exclude gastrointestinal cancer predispositions, we performed genetic testing with a multigene panel in all available PED patients (five out of eight) that resulted not-informative in all of them^32^.

For all our mutational, pathways, CNVs analysis of our EO-CRC cohort, a control group of 56 sporadic AD-CRC was identified within our Institutional Molecular Tumor Board Database^33^ according to the following criteria: (i) having the diagnosis of CRC, (ii) being of age > 60, so that the genomic landscape observed represented that of AD-CRC, (iii) with available NGS Oncomine Comprehensive Assay Plus DNA analysis.

The study and all experimental protocols were approved by Fondazione IRCCS Istituto Nazionale dei Tumori di Milano Internal Audit Committee and the Ethics Committee of our Institute (CE N. INT 81/19), and all experiments were performed in accordance with relevant guidelines and regulations. All patients, and/or their guardians, consented to the the study by signing an informed consent to research activities at the time of admission to the hospital.

### DNA extraction and next-generation sequencing

FFPE 5 µm cut sections were manually microdissected to isolate the highest possible percentage of neoplastic cells. Tumor DNA was extracted using the QIAamp Gene Read DNA FFPE kit (Qiagen, Venlo, the Netherlands); this kit includes enzymatic removal of the artifacts caused by formalin fixation and aging. DNA concentration was quantified using Qubit^™^ ds DNA High-Sensitive Assay kit on the Qubit fluorometer (Thermo Fisher Scientific; Waltham, MA, USA).

Target NGS was performed using Oncomine Comprehensive Assay Plus (Thermo Fisher Scientific), a targeted panel, that in addition to be specifically designed for the detection of mutations in about 500 genes of solid tumors, also provides information regarding CNVs, MSI and TMB. Briefly, 20 ng of DNA was used to generate libraries with Ion Ampliseq^™^ library kit plus (Thermo Fisher Scientific) according to the manufacturer’s instructions. Libraries were quantified by ion library TaqMan quantification kit and were sequenced on the Ion GeneStudio S5 Prime (Thermo Fisher Scientific) using Ion Chip 550 and Ion 550 Kit Chef according to the manufacturer’s instructions.

### Data analysis

Sequencing data were processed using the Ion Torrent platform-specific software Torrent Suite Software^™^ (version 5.12) to generate sequence reads, alignment of the reads on the reference genome hg19, trim adapter sequences, filter and remove poor signal-profile reads. The variant calling from the sequencing data was generated using the Variant Caller plugin. We applied some filters to that plugin to eliminate erroneous base calling: we set each variant coverage > 40, a variant frequency on each sample > 2% and a quality value > 30; the average base coverage depth was always more than 500× . Data were analyzed by Ion Reporter^™^ analysis software version 5.18 with Oncomine Comprehensive Plus—w2.2—DNA—Single Sample workflow and OpenCravat tool. Germline variants were filtered by using publically‐available or proprietary database of known polymorphisms (e.g. dbSNP, ExAC, 1000Genomes), excluding variant with MAF > 10^−5^.

The filtered variants were examined using the integrative genomic viewer IGV tool and were further clinically annotated using ClinVar^34^ and cBio portal database; variants classified as benign or likely benign were excluded^35–37^.

For CNVs analysis, only samples with a median of the absolute value of all pairwise differences (MAPD) < 0.5 can be considered for CNVs detection.

TMB was calculated for each sample by Ion reporter software and was classified as low (< 10 mutations/Mb) or high (≥ 10 mutations/Mb).

### Microsatellite instability determination

The MSI status of tumors was assessed by fluorescent pentaplex PCR of 5 quasimonomorphic mononucleotide repeats, as previously described^38^. Tumors were scored as stable (MSS) if none of the markers for MSI were present, MSI-low (L-MSI) if one marker was present and MSI-high (H-MSI) when if two or more markers were present.

### Bioinformatic analysis and statistical methods

Top 30 altered genes were extracted and marked differently based on the variants class detected (missense, splice, trunc, inframe) and their clinical significance based on ClinVar database.

Descriptive and comparative analysis of mutational data associated to each group of patients (0–19; 20–39; Over 60) were performed using maftools R package^39^. In particular, pathway analysis was performed considering canonical oncogenic signaling pathways defined by Vega and colleagues through ad hoc function implemented in the package^11^. Comparison of oncogenic pathway alteration frequencies among 0–19, 20–39 and Over 60 patients was performed by considering that a case was altered in a given pathway if one or more pathway genes had a pathogenic alteration, as previously described.

Moreover, Kegg database was interrogated through ClusterProfiler R package to identify significantly altered pathways (pVal < 0.01)^40,41^.

Fisher’s exact *t*-test was used to find group-specific altered genes. Regarding group-specific altered genes, two type of comparison were performed: paired, where FDR < 0.05 was considered to define the level of significance; groupwise (0–19 vs other; 20–39 vs other; Over 60 vs other), where p-value < 0.05 was considered to define the level of significance. Due to the different distribution of samples between paired and groupwise comparison, we selected FDR or p-value thresholds to obtain a restricted number of genes. T-test was used to find significant difference between TMB values in patient groups (0–19; 20–39; Over 60).

All bioinformatics and statistical analysis were performed considering pathogenic, likely pathogenic and VUS variants.

### Supplementary Information

Supplementary Figure S1.

Supplementary Tables.

## Data Availability

The datasets used and/or analyzed during the current study available from the corresponding author on reasonable request.
