# Supplementary material for: Molecular profiling of pediatric and young adult colorectal cancer reveals a distinct genomic landscapes and potential therapeutic avenues
Source: Sci Rep. 2024 Jun 7;14:13138. doi: 10.1038/s41598-024-64149-7 (PMC11161608; doi:10.1038/s41598-024-64149-7)

Supplementary Fig. 1. Distribution of RNF43 mutations in pediatric, young and adult patients.[15,16]

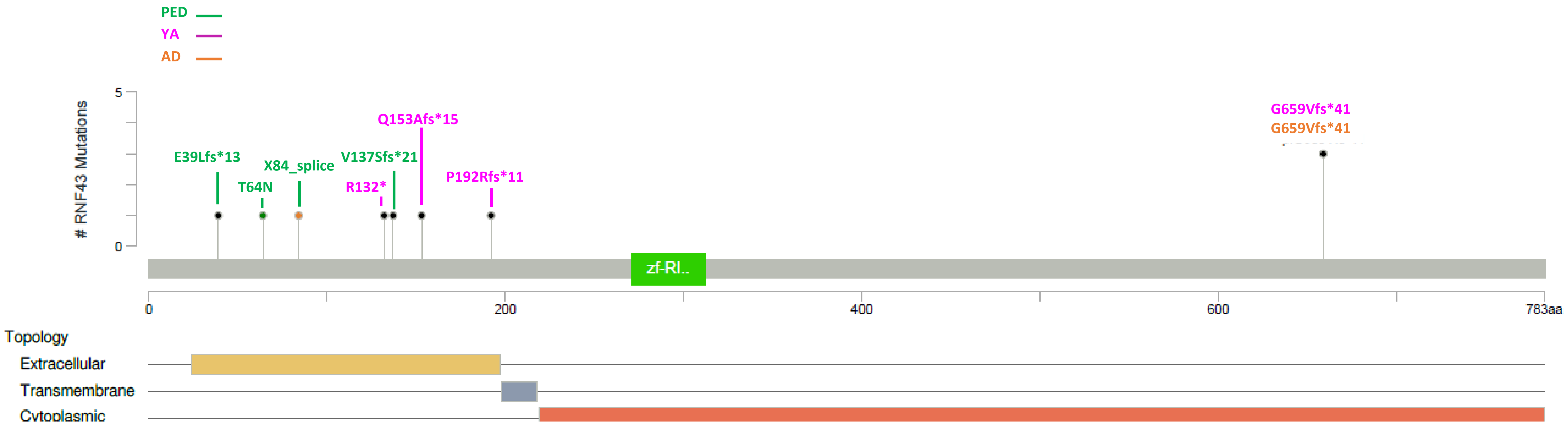

Supplement: Supplementary file 1 — Supplementary Figure S1. [file 41598_2024_64149_MOESM1_ESM.pdf]
