# Supplementary material for: Molecular profiling of pediatric and young adult colorectal cancer reveals a distinct genomic landscapes and potential therapeutic avenues
Source: Sci Rep. 2024 Jun 7;14:13138. doi: 10.1038/s41598-024-64149-7 (PMC11161608; doi:10.1038/s41598-024-64149-7)
Supplement: Supplementary file 2 — Supplementary Tables. [file 41598_2024_64149_MOESM2_ESM.pdf]

Samples	MSS	TMB	Gene	mutation type	cDNA_chang	Protein_Change	VAF	clinical significance	chr	position
PED1	H-MSI	23,85	SDHB	MISSENSE	c.809T>C	p.Met270Thr	25%	VUS	chr1	17345410
PED1	H-MSI	23,85	RAD54L	MISSENSE	c.1535G>A	p.Ser512Asn	6%	VUS	chr1	46739344
PED1	H-MSI	23,85	JAK1	MISSENSE	c.1759G>A	p.Glu587Lys	25%	VUS	chr1	65313355
PED1	H-MSI	23,85	CTLA4	MISSENSE	c.160G>A	p.Ala54Thr	5%	VUS	chr2	204735359
PED1	H-MSI	23,85	SETD2	TRUNC	c.1102C>T	p.Arg368Ter	8%	Likely pathogenic	chr3	47165024
PED1	H-MSI	23,85	ATR	MISSENSE	c.4435T>C	p.Tyr1479His	22%	VUS	chr3	142234305
PED1	H-MSI	23,85	INPP4B	MISSENSE	c.1087G>A	p.Val363Ile	47%	VUS	chr4	143114334
PED1	H-MSI	23,85	FAT1	MISSENSE	c.2200T>C	p.Ser734Pro	20%	VUS	chr4	187628782
PED1	H-MSI	23,85	FLT4	TRUNC	c.3699G>A	p.Trp1233Ter	8%	VUS	chr5	180037013
PED1	H-MSI	23,85	ANKRD61	MISSENSE	c.11T>C	p.Ile4Thr	50%	VUS	chr7	6071017
PED1	H-MSI	23,85	TRRAP	MISSENSE	c.2446C>T	p.Arg816Trp	4%	VUS	chr7	98515126
PED1	H-MSI	23,85	CUL1	MISSENSE	c.1453G>A	p.Glu485Lys	7%	VUS	chr7	148484186
PED1	H-MSI	23,85	PPP2R2A	MISSENSE	c.1267G>A	p.Ala423Thr	5%	VUS	chr8	26227852
PED1	H-MSI	23,85	LARP4B	MISSENSE	c.346C>A	p.Pro116Thr	5%	VUS	chr10	909767
PED1	H-MSI	23,85	FGFR2	MISSENSE	c.670T>C	p.Ser224Pro	5%	VUS	chr10	123298184
PED1	H-MSI	23,85	ARL2	MISSENSE	c.446G>A	p.Ser149Asn	19%	VUS	chr11	64789218
PED1	H-MSI	23,85	KRAS	MISSENSE	c.35G>A	p.Gly12Asp	23%	Pathogenic	chr12	25398284
PED1	H-MSI	23,85	ARID2	MISSENSE	c.3893A>G	p.Lys1298Arg	52%	VUS	chr12	46245799
PED1	H-MSI	23,85	KMT2D	MISSENSE	c.15322C>T	p.Arg5108Cys	26%	Likely pathogenic	chr12	49420427
PED1	H-MSI	23,85	PCNX1	MISSENSE	c.1733G>T	p.Arg578Met	26%	VUS	chr14	71444787
PED1	H-MSI	23,85	TSC2	MISSENSE	c.3347C>A	p.Ala1116Asp	33%	VUS	chr16	2129620
PED1	H-MSI	23,85	TP53	MISSENSE	c.546C>A	p.Cys182Ter	6%	Likely pathogenic	chr17	7578384
PED1	H-MSI	23,85	MYO15A	MISSENSE	c.7873G>A	p.Ala2625Thr	50%	VUS	chr17	18055245
PED1	H-MSI	23,85	RNF43	MISSENSE	c.191C>A	p.Thr64Asn	23%	VUS	chr17	56492748
PED1	H-MSI	23,85	NOTCH3	TRUNC	c.3584delC	p.Pro1195GlnfsTer77	23%	Likely pathogenic	chr19	15289969
PED1	H-MSI	23,85	POLD1	TRUNC	c.193delG	p.Val65LeufsTer11	28%	VUS	chr19	50902296
PED1	H-MSI	23,85	PPP2R1A	MISSENSE	c.553G>A	p.Ala185Thr	19%	VUS	chr19	52715988
PED1	H-MSI	23,85	RUNX1	MISSENSE	c.1093G>A	p.Gly365Ser	29%	VUS	chr21	36164782
PED1	H-MSI	23,85	PUDP	MISSENSE	c.449A>T	p.His150Leu	44%	VUS	chrX	6995391
PED1	H-MSI	23,85	KDM5C	MISSENSE	c.1168C>T	p.Arg390Trp	45%	VUS	chrX	53241043
PED1	H-MSI	23,85	JAK1	MISSENSE	c.289G>A	p.Val97Ile	45%	VUS	chr1	65344748
PED1	H-MSI	23,85	TRPM3	TRUNC	c.5062C>T	p.Arg1688Ter	50%	VUS	chr9	73150943

PED1	H-MSI	23,85	DPCD	MISSENSE	c.484G>A	p.Ala162Thr	27%	VUS	chr10	103368671
PED1	H-MSI	23,85	CALCA	MISSENSE	c.276G>A	p.Met92Ile	53%	VUS	chr11	14990495
PED1	H-MSI	23,85	KMT2B	MISSENSE	c.2702G>A	p.Arg901Gln	22%	VUS	chr19	36213600
PED2	MSS	54,88	APC	TRUNC	c.2054G>A	p.Trp685Ter	11%	Pathogenic	chr5	112173345
PED2	MSS	54,88	PIK3CA	MISSENSE	c.3140A>G	p.His1047Arg	8%	Pathogenic	chr3	178952085
PED2	MSS	54,88	KRAS	MISSENSE	c.351A>T	p.Lys117Asn	11%	Pathogenic	chr12	25378647
PED2	MSS	54,88	TP53	TRUNC	c.658del	p.Tyr220MetfsTer27	11%	Pathogenic	chr17	7578191
PED2	MSS	54,88	PMS2	TRUNC	c.683del	p.Gly228AlafsTer30	14%	Likely pathogenic	chr7	6038761
PED2	MSS	54,88	AMER1	TRUNC	c.1489C>T	p.Arg497Ter	8%	Likely pathogenic	chrX	63411678
PED2	MSS	54,88	B2M	SPLICE	c.68-2A>G		9%	Likely pathogenic	chr15	45007619
PED2	MSS	54,88	FBXW7	TRUNC	c.1952T>A	p.Leu651Ter	9%	Likely pathogenic	chr4	153244205
PED2	MSS	54,88	SPEN	MISSENSE	c.10507A>G	p.Lys3503Glu	8%	VUS	chr1	16264138
PED2	MSS	54,88	JAK1	MISSENSE	c.382C>T	p.Arg128Cys	9%	VUS	chr1	65339154
PED2	MSS	54,88	NOTCH2	MISSENSE	c.3183G>T	p.Gln1061His	10%	VUS	chr1	120483178
PED2	MSS	54,88	LHX4	MISSENSE	c.973C>T	p.Pro325Ser	10%	VUS	chr1	180243514
PED2	MSS	54,88	GALNT5	MISSENSE	c.152G>T	p.Arg51Met	9%	VUS	chr2	158114746
PED2	MSS	54,88	BARD1	MISSENSE	c.2110A>G	p.Ser704Gly	10%	VUS	chr2	215593624
PED2	MSS	54,88	ATR	MISSENSE	c.4273C>T	p.Leu1425Phe	12%	VUS	chr3	142238620
PED2	MSS	54,88	PDGFRA	TRUNC	c.1753A>T	p.Arg585Ter	8%	VUS	chr4	55141107
PED2	MSS	54,88	ERAP1	MISSENSE	c.857C>T	p.Ala286Val	10%	VUS	chr5	96130807
PED2	MSS	54,88	PSMB9	MISSENSE	c.640C>T	p.Pro214Ser	9%	VUS	chr6	32827289
PED2	MSS	54,88	ROS1	MISSENSE	c.6289A>T	p.Ile2097Leu	9%	VUS	chr6	117631389
PED2	MSS	54,88	ABCB1	SPLICE	c.702+2T>C		8%	VUS	chr7	87195384
PED2	MSS	54,88	BRAF	MISSENSE	c.2324G>A	p.Arg735Gln	9%	VUS	chr7	140434494
PED2	MSS	54,88	KMT2C	MISSENSE	c.12023A>G	p.Glu4008Gly	11%	VUS	chr7	151851468
PED2	MSS	54,88	PXDNL	MISSENSE	c.1561A>G	p.Ser521Gly	9%	VUS	chr8	52339283
PED2	MSS	54,88	DCAF4L2	MISSENSE	c.871A>T	p.Thr291Ser	10%	VUS	chr8	88885329
PED2	MSS	54,88	CSMD3	MISSENSE	c.6077A>G	p.Tyr2026Cys	9%	VUS	chr8	113392640
PED2	MSS	54,88	RECQL4	MISSENSE	c.2224G>A	p.Glu742Lys	13%	VUS	chr8	145738841
PED2	MSS	54,88	IFNB1	MISSENSE	c.175T>C	p.Phe59Leu	11%	VUS	chr9	21077694
PED2	MSS	54,88	PAX5	MISSENSE	c.267G>T	p.Lys89Asn	9%	VUS	chr9	37015137
PED2	MSS	54,88	MAPK8	MISSENSE	c.569A>G	p.Tyr190Cys	10%	VUS	chr10	49628316
PED2	MSS	54,88	MAPK8	MISSENSE	c.884G>T	p.Arg295Met	8%	VUS	chr10	49634435

PED2	MSS	54,88	ATM	MISSENSE	c.7624A>G	p.Asn2542Asp	9%	VUS	chr11	108202279
PED2	MSS	54,88	KMT2A	MISSENSE	c.5960A>G	p.Glu1987Gly	11%	VUS	chr11	118369242
PED2	MSS	54,88	KMT2D	MISSENSE	c.13130_131	p.Trp4377Tyr	10%	VUS	chr12	49425357
PED2	MSS	54,88	STAT6	MISSENSE	c.2014C>A	p.Leu672Ile	8%	VUS	chr12	57492627
PED2	MSS	54,88	TBX3	MISSENSE	c.782T>C	p.Val261Ala	11%	VUS	chr12	115117332
PED2	MSS	54,88	RB1	MISSENSE	c.826G>T	p.Val276Phe	11%	VUS	chr13	48937058
PED2	MSS	54,88	FANCM	MISSENSE	c.5987C>T	p.Ser1996Phe	10%	VUS	chr14	45668117
PED2	MSS	54,88	TSC2	MISSENSE	c.1154A>G	p.His385Arg	8%	VUS	chr16	2111906
PED2	MSS	54,88	TSC2	MISSENSE	c.5216A>G	p.Lys1739Arg	8%	VUS	chr16	2138283
PED2	MSS	54,88	MAP2K4	MISSENSE	c.974C>T	p.Pro325Leu	9%	VUS	chr17	12032538
PED2	MSS	54,88	ERBB2	MISSENSE	c.3142C>T	p.Arg1048Cys	12%	VUS	chr17	37883239
PED2	MSS	54,88	PPM1D	MISSENSE	c.1289T>C	p.Val430Ala	8%	VUS	chr17	58740384
PED2	MSS	54,88	BRIP1	MISSENSE	c.1208G>A	p.Arg403Gln	10%	VUS	chr17	59876593
PED2	MSS	54,88	AXIN2	MISSENSE	c.1481C>T	p.Pro494Leu	18%	VUS	chr17	63533673
PED2	MSS	54,88	AXIN2	MISSENSE	c.1291T>C	p.Tyr431His	10%	VUS	chr17	63533863
PED2	MSS	54,88	JAK3	MISSENSE	c.187A>G	p.Ile63Val	15%	VUS	chr19	17954707
PED2	MSS	54,88	ERCC2	MISSENSE	c.1066G>A	p.Ala356Thr	11%	VUS	chr19	45867053
PED2	MSS	54,88	POLD1	MISSENSE	c.946G>A	p.Asp316Asn	10%	VUS	chr19	50905974
PED2	MSS	54,88	ASXL1	MISSENSE	c.4600T>C	p.Cys1534Arg	5%	VUS	chr20	31025115
PED2	MSS	54,88	TPTE	MISSENSE	c.439G>A	p.Val147Ile	22%	VUS	chr21	10951273
PED2	MSS	54,88	KDM6A	MISSENSE	c.1108A>T	p.Ile370Phe	10%	VUS	chrX	44918697
PED2	MSS	54,88	AMER1	MISSENSE	c.2245T>C	p.Ser749Pro	6%	VUS	chrX	63410922
PED2	MSS	54,88	ZMYM3	MISSENSE	c.2893C>A	p.Leu965Met	9%	VUS	chrX	70465303
PED2	MSS	54,88	ZMYM3	MISSENSE	c.1961G>T	p.Ser654Ile	9%	VUS	chrX	70468026
PED2	MSS	54,88	SATL1	MISSENSE	c.356C>T	p.Pro119Leu	11%	VUS	chrX	84363619
PED2	MSS	54,88	ZNF138	MISSENSE	c.287G>C	p.Arg96Thr	8%	VUS	chr7	64291907
PED3	MSS	6,8	ZDBF2	MISSENSE	c.5084A>G	p.Asn1695Ser	61%	VUS	chr2	207174336
PED3	MSS	6,8	SDHA	MISSENSE	c.1133C>T	p.Thr378Met	60%	VUS	chr5	235327
PED3	MSS	6,8	GLT6D1	MISSENSE	c.721G>C	p.Asp241His	56%	VUS	chr9	138516053
PED3	MSS	6,8	ATM	TRUNC	c.3699_3703	p.Pro1235TyrfsTer9	12%	Pathogenic	chr11	108153558
PED3	MSS	6,8	KMT2D	MISSENSE	c.8521C>A	p.Pro2841Thr	3%	VUS	chr12	49432618
PED3	MSS	6,8	RNF43	TRUNC	c.113_114du	p.Glu39LeufsTer13	27%	Likely pathogenic	chr17	56492824
PED4	MSS	2,85	TP53	MISSENSE	c.730G>A	p.Gly244Ser	16%	Pathogenic	chr17	7577551

PED4	MSS	2,85	ERBB4	MISSENSE	c.2393T>G	p.Leu798Arg	24%	Likely pathogenic	chr2	212426722
PED4	MSS	2,85	FANCA	MISSENSE	c.4199G>A	p.Arg1400His	26%	VUS	chr16	89805351
PED4	MSS	2,85	SPTA1	MISSENSE	c.4641A>C	p.Glu1547Asp	20%	VUS	chr1	158612297
PED4	MSS	2,85	TRIM51	MISSENSE	c.1282A>C	p.Ser428Arg	11%	VUS	chr11	55659031
PED4	MSS	2,85	ASXL1	MISSENSE	c.656G>T	p.Gly219Val	49%	VUS	chr20	31017794
PED5	MSS	15,25	TP53	MISSENSE	c.817C>T	p.Arg273Cys	32%	Pathogenic	chr17	7577121
PED5	MSS	15,25	RNF43	SPLICE	c.252+2C>G		31%	Likely pathogenic	chr17	56492685
PED5	MSS	15,25	SMAD2	MISSENSE	c.556A>T	p.Ile186Phe	45%	VUS	chr18	45394793
PED5	MSS	15,25	MUTYH	MISSENSE	c.488G>A	p.Arg163Gln	43%	VUS	chr1	45798439
PED5	MSS	15,25	OR2W3	MISSENSE	c.70G>A	p.Glu24Lys	11%	VUS	chr1	248058958
PED5	MSS	15,25	GLI3	MISSENSE	c.3677C>T	p.Pro1226Leu	52%	VUS	chr7	42004994
PED5	MSS	15,25	GLI3	MISSENSE	c.3116C>A	p.Ala1039Glu	50%	VUS	chr7	42005555
PED5	MSS	15,25	TCF7L2	SPLICE	c.382-2_382del		57%	VUS	chr10	114724312
PED5	MSS	15,25	ATRX	MISSENSE	c.4141G>T	p.Asp1381Tyr	8%	VUS	chrX	76912123
PED5	MSS	15,25	MYCN	MISSENSE	c.691G>C	p.Ala231Pro	27%	VUS	chr2	16082877
PED6	MSS	5,88	SMAD4	MISSENSE	c.1081C>T	p.Arg361Cys	10%	Pathogenic	chr18	48591918
PED6	MSS	5,88	TP53	MISSENSE	c.358A>G	p.Lys120Glu	14%	Likely pathogenic	chr17	7579329
PED6	MSS	5,88	RNF43	TRUNC	c.409del	p.Val137SerfsTer21	21%	Likely pathogenic	chr3	178952094
PED6	MSS	5,88	PIK3CA	MISSENSE	c.3149G>C	p.Gly1050Ala	6%	VUS	chr1	57340655
PED6	MSS	5,88	C8A	MISSENSE	c.205T>G	p.Phe69Val	46%	VUS	chr3	52662944
PED6	MSS	5,88	PBRM1	MISSENSE	c.1409A>G	p.Tyr470Cys	49%	VUS	chr4	1976630
PED6	MSS	5,88	NSD2	MISSENSE	c.3413G>A	p.Arg1138Gln	3%	VUS	chr4	41984107
PED6	MSS	5,88	DCAF4L1	MISSENSE	c.298G>A	p.Gly100Ser	21%	VUS	chr6	106553668
PED6	MSS	5,88	PRDM1	MISSENSE	c.1633G>A	p.Glu545Lys	5%	VUS	chr9	98224206
PED6	MSS	5,88	PTCH1	MISSENSE	c.2635G>A	p.Asp879Asn	3%	VUS	chr11	69458730
PED6	MSS	5,88	CCND1	MISSENSE	c.545C>T	p.Ala182Val	4%	VUS	chr11	118343235
PED6	MSS	5,88	KMT2A	MISSENSE	c.1361C>T	p.Pro454Leu	3%	VUS	chr15	44061839
PED6	MSS	5,88	PDIA3	MISSENSE	c.1261G>A	p.Glu421Lys	5%	VUS	chr15	52643623
PED6	MSS	5,88	MYO5A	MISSENSE	c.3677G>A	p.Arg1226His	4%	VUS	chr16	3639473
PED6	MSS	5,88	SLX4	MISSENSE	c.4166C>T	p.Ala1389Val	47%	VUS	chr17	56440928
PED6	MSS	5,88	NOTCH3	MISSENSE	c.5524G>A	p.Ala1842Thr	4%	VUS	chr19	15276741
PED6	MSS	5,88	RASA1	MISSENSE	c.566C>T	p.Thr189Met	3%	VUS	chr5	86627191
PED6	MSS	5,88	CYP2D6	SPLICE	c.506-1G>A	p.?	41%	VUS	chr22	42524947

PED7	H-MSI	42,91	PIK3CA	MISSENSE	c.3140A>G	p.His1047Arg	28%	Pathogenic	chr3	178952085
PED7	H-MSI	42,91	TP53	MISSENSE	c.638G>A	p.Arg213Gln	27%	Pathogenic	chr17	7578211
PED7	H-MSI	42,91	TGFBR2	MISSENSE	c.1658G>A	p.Arg553His	23%	Pathogenic	chr3	30732970
PED7	H-MSI	42,91	FBXW7	MISSENSE	c.1393C>T	p.Arg465Cys	8%	Likely pathogenic	chr4	153249385
PED7	H-MSI	42,91	APC	TRUNC	c.1495C>T	p.Arg499Ter	19%	Pathogenic	chr5	112162891
PED7	H-MSI	42,91	APC	TRUNC	c.3682C>T	p.Gln1228Ter	23%	Pathogenic	chr5	112174973
PED7	H-MSI	42,91	MSH2	TRUNC	c.1147C>T	p.Arg383Ter	22%	Pathogenic	chr2	47656951
PED7	H-MSI	42,91	TET2	TRUNC	c.1740delA	p.Lys580AsnfsTer21	15%	Likely pathogenic	chr4	106156835
PED7	H-MSI	42,91	POLE	MISSENSE	c.3323A>G	p.His1108Arg	22%	VUS	chr12	133234509
PED7	H-MSI	42,91	BRCA1	MISSENSE	c.1492C>A	p.Leu498Ile	26%	VUS	chr17	41246056
PED7	H-MSI	42,91	BRCA2	MISSENSE	c.6250G>A	p.Asp2084Asn	3%	VUS	chr13	32914742
PED7	H-MSI	42,91	JAK1	MISSENSE	c.1091A>T	p.Asn364Ile	25%	VUS	chr1	65330555
PED7	H-MSI	42,91	DPYD	MISSENSE	c.874G>A	p.Ala292Thr	25%	VUS	chr1	98060699
PED7	H-MSI	42,91	COL11A1	MISSENSE	c.2490A>C	p.Gln830His	11%	VUS	chr1	103453201
PED7	H-MSI	42,91	MCL1	MISSENSE	c.653A>G	p.Asp218Gly	21%	VUS	chr1	150551354
PED7	H-MSI	42,91	MYCN	MISSENSE	c.691G>C	p.Ala231Pro	42%	VUS	chr2	16082877
PED7	H-MSI	42,91	ASXL2	MISSENSE	c.3566A>T	p.Glu1189Val	4%	VUS	chr2	25965640
PED7	H-MSI	42,91	SLC9A4	MISSENSE	c.323C>T	p.Ala108Val	20%	VUS	chr2	103095364
PED7	H-MSI	42,91	SETD2	MISSENSE	c.6901A>C	p.Thr2301Pro	23%	VUS	chr3	47098373
PED7	H-MSI	42,91	SETD2	MISSENSE	c.3763C>A	p.Leu1255Ile	21%	VUS	chr3	47162363
PED7	H-MSI	42,91	RAD50	MISSENSE	c.584T>C	p.Val195Ala	38%	VUS	chr5	131915586
PED7	H-MSI	42,91	SPINK5	MISSENSE	c.1171C>A	p.Pro391Thr	18%	VUS	chr5	147480095
PED7	H-MSI	42,91	ADAMTS2	MISSENSE	c.1861G>T	p.Asp621Tyr	23%	VUS	chr5	178564860
PED7	H-MSI	42,91	H3C2	MISSENSE	c.299A>G	p.Tyr100Cys	20%	VUS	chr6	26031990
PED7	H-MSI	42,91	MAP3K4	MISSENSE	c.371C>A	p.Pro124Gln	21%	VUS	chr6	161469675
PED7	H-MSI	42,91	MVB12B	MISSENSE	c.88T>C	p.Ser30Pro	4%	VUS	chr9	129102793
PED7	H-MSI	42,91	PSD	TRUNC	c.823delG	p.Val275TrpfsTer19	21%	VUS	chr10	104174920
PED7	H-MSI	42,91	ACVR1B	MISSENSE	c.803A>G	p.Asp268Gly	22%	VUS	chr12	52374975
PED7	H-MSI	42,91	ACVR1B	MISSENSE	c.956T>C	p.Leu319Pro	5%	VUS	chr12	52377804
PED7	H-MSI	42,91	FLT3	MISSENSE	c.2039C>T	p.Ala680Val	16%	VUS	chr13	28602329
PED7	H-MSI	42,91	MGA	MISSENSE	c.418C>T	p.Arg140Cys	3%	VUS	chr15	41961510
PED7	H-MSI	42,91	NQO1	MISSENSE	c.620G>A	p.Gly207Glu	11%	VUS	chr16	69745084
PED7	H-MSI	42,91	COG4	MISSENSE	c.2162C>T	p.Thr721Met	20%	VUS	chr16	70515335

PED7	H-MSI	42,91	NCOR1	MISSENSE	c.6454C>A	p.Pro2152Thr	27%	VUS	chr17	15952241
PED7	H-MSI	42,91	SETBP1	MISSENSE	c.1693A>G	p.Thr565Ala	24%	VUS	chr18	42530998
PED7	H-MSI	42,91	SMAD2	MISSENSE	c.659C>T	p.Thr220Met	21%	VUS	chr18	45391501
PED7	H-MSI	42,91	IGFLR1	MISSENSE	c.7996G>A, (	p.Val2666Met	20%	VUS	chr19	36229306
PED7	H-MSI	42,91	CIC	MISSENSE	c.3704T>A	p.Leu1235Gln	24%	VUS	chr19	42797342
PED7	H-MSI	42,91	PTPRT	MISSENSE	c.3649C>T	p.Leu1217Phe	16%	VUS	chr20	40730886
PED7	H-MSI	42,91	RUNX1	MISSENSE	c.1220A>G	p.Tyr407Cys	25%	VUS	chr21	36164655
PED7	H-MSI	42,91	CHEK2	MISSENSE	c.839T>C	p.Leu280Pro	17%	VUS	chr22	29106001
PED7	H-MSI	42,91	EP300	MISSENSE	c.2708C>T	p.Ala903Val	32%	VUS	chr22	41546093
PED7	H-MSI	42,91	ZMYM3	TRUNC	c.3667C>T	p.Gln1223Ter	21%	Likely pathogenic	chrX	70462155
PED7	H-MSI	42,91	ZMYM3	MISSENSE	c.770C>T	p.Thr257Ile	10%	VUS	chrX	70471036
PED8	MSS	7,64	PIK3CA	MISSENSE	c.1624G>A	p.Glu542Lys	10%	Pathogenic	chr3	178936082
PED8	MSS	7,64	BRCA1	TRUNC	c.3481_3491	p.Glu1161PhefsTer3	18%	Pathogenic	chr17	41244056
PED8	MSS	7,64	TP53	TRUNC	c.629_630in	p.Asn210LysfsTer6	12%	Pathogenic	chr17	7578220
PED8	MSS	7,64	CTNNB1	MISSENSE	c.1364G>A	p.Gly455Asp	12%	VUS	chr3	41275198
PED8	MSS	7,64	PRRT3	MISSENSE	c.646G>A	p.Gly216Ser	5%	VUS	chr3	9991154
PED8	MSS	7,64	ATR	MISSENSE	c.1432G>C	p.Glu478Gln	50%	VUS	chr3	142279214
PED8	MSS	7,64	CTNND2	MISSENSE	c.376T>C	p.Ser126Pro	11%	VUS	chr5	11411711
PED8	MSS	7,64	C6	MISSENSE	c.1393G>A	p.Glu465Lys	5%	VUS	chr5	41161860
PED8	MSS	7,64	CANX	SPLICE	c.529-1G>C		3%	VUS	chr5	179136873
PED8	MSS	7,64	NOTCH1	MISSENSE	c.5302T>C	p.Trp1768Arg	3%	VUS	chr9	139396806
PED8	MSS	7,64	CYP4F12	MISSENSE	c.1543C>T	p.Arg515Trp	4%	VUS	chr19	15807863
PED8	MSS	7,64	RBM10	MISSENSE	c.683G>A	p.Arg228Gln	7%	VUS	chrX	47032582
YA1	MSS	3,82	RNF43	TRUNC	c.456dup	p.Gln153AlafsTer15	40%	Pathogenic	chr17	56440762
YA1	MSS	3,82	SMAD4	MISSENSE	c.1082G>A	p.Arg361His	36%	Pathogenic	chr18	48591919
YA1	MSS	3,82	TP53	MISSENSE	c.475G>C	p.Ala159Pro	36%	VUS	chr17	7578455
YA1	MSS	3,82	TAP1	TRUNC	c.639delC	p.Trp214GlyfsTer15	51%	VUS	chr6	32820239
YA1	MSS	3,82	RAD52	TRUNC	c.962_965de	p.Leu321SerfsTer13	43%	Likely pathogenic	chr12	1023600
YA1	MSS	3,82	FGFR1	MISSENSE	c.1655C>T	p.Thr552Met	16%	VUS	chr8	38274832
YA1	MSS	3,82	CEP128	MISSENSE	c.2190G>T	p.Glu730Asp	19%	VUS	chr14	81251260
YA1	MSS	3,82	MGA	MISSENSE	c.4796C>T	p.Thr1599Ile	52%	VUS	chr15	42034954
YA1	MSS	3,82	DSC1	MISSENSE	c.638A>G	p.Tyr213Cys	27%	VUS	chr18	28728595
YA1	MSS	3,82	ZNF217	MISSENSE	c.1774C>T	p.Leu592Phe	78%	VUS	chr20	52193529

YA1	MSS	3,82	TPTE	SPLICE	c.1521-1G>T		18%	VUS	chr21	10907041
YA2	MSS	7,75	TP53	MISSENSE	c.524G>A	p.Arg175His	26%	Pathogenic	chr17	7578406
YA2	MSS	7,75	RAD50	TRUNC	c.3454C>T	p.Arg1152Ter	30%	Pathogenic	chr5	131972871
YA2	MSS	7,75	BRCA2	TRUNC	c.9784C>T	p.Gln3262Ter	8%	Likely pathogenic	chr13	32972434
YA2	MSS	7,75	CLCA2	MISSENSE	c.2600G>A	p.Arg867Gln	38%	VUS	chr1	86920978
YA2	MSS	7,75	SLC8A1	MISSENSE	c.1840G>A	p.Glu614Lys	10%	VUS	chr2	40405602
YA2	MSS	7,75	MSH2	MISSENSE	c.578A>C	p.Gln193Pro	62%	VUS	chr2	47637444
YA2	MSS	7,75	DCAF4L1	MISSENSE	c.298G>A	p.Gly100Ser	9%	VUS	chr4	41984107
YA2	MSS	7,75	CDH10	MISSENSE	c.206G>T	p.Gly69Val	9%	VUS	chr5	24593394
YA2	MSS	7,75	SNX18	MISSENSE	c.82A>G	p.Ser28Gly	59%	VUS	chr5	53813864
YA2	MSS	7,75	DAXX	MISSENSE	c.334T>G	p.Leu112Val	9%	VUS	chr6	33289254
YA2	MSS	7,75	DAXX	MISSENSE	c.305A>T	p.Asn102Ile	9%	VUS	chr6	33289283
YA2	MSS	7,75	TSHR	MISSENSE	c.1725G>C	p.Glu575Asp	11%	VUS	chr14	81610127
YA2	MSS	7,75	RIT2	MISSENSE	c.632A>C	p.Lys211Thr	13%	VUS	chr18	40323480
YA3	MSS	3,8	TP53	SPLICE	c.993+1G>A		19%	Pathogenic	chr17	7576852
YA3	MSS	3,8	CDHR4	TRUNC	c.220C>T	p.Gln74Ter	36%	VUS	chr3	49836700
YA3	MSS	3,8	KDR	MISSENSE	c.2081C>A	p.Pro694His	19%	VUS	chr4	55968582
YA3	MSS	3,8	TET2	MISSENSE	c.3442G>C	p.Val1148Leu	61%	VUS	chr4	106158541
YA3	MSS	3,8	NPY4R	MISSENSE	c.715C>T	p.Arg239Trp	27%	VUS	chr10	47087498
YA3	MSS	3,8	SCGB1D2	MISSENSE	c.137T>C	p.Leu46Pro	47%	VUS	chr11	62010842
YA3	MSS	3,8	CIITA	MISSENSE	c.680C>A	p.Pro227His	56%	VUS	chr16	10996563
YA3	MSS	3,8	CYLD	MISSENSE	c.1669C>T	p.Arg557Cys	8%	VUS	chr16	50815307
YA3	MSS	3,8	CNTNAP4	MISSENSE	c.340A>G	p.Ser114Gly	90%	VUS	chr16	76389358
YA3	MSS	3,8	AXIN2	MISSENSE	c.1481C>T	p.Pro494Leu	51%	VUS	chr17	63533673
YA4	MSS	7,7	TP53	MISSENSE	c.844C>T	p.Arg282Trp	64%	Pathogenic	chr17	7577094
YA4	MSS	7,7	NOTCH3	TRUNC	c.937C>T	p.Gln313Ter	26%	Likely pathogenic	chr19	15302334
YA4	MSS	7,7	HRAS	TRUNC	c.492_508de	p.Gln165SerfsTer2	26%	VUS	chr11	532698
YA4	MSS	7,7	RASA1	INFRAME	c.285_305de	p.Ala100_Ala106del	21%	VUS	chr5	86564540
YA4	MSS	7,7	BRINP3	MISSENSE	c.539T>G	p.Leu180Arg	6%	VUS	chr1	190234074
YA4	MSS	7,7	MSH6	MISSENSE	c.1894A>G	p.Lys632Glu	39%	VUS	chr2	48027016
YA4	MSS	7,7	PCBP1	MISSENSE	c.136C>G	p.Arg46Gly	26%	VUS	chr2	70315011
YA4	MSS	7,7	CNTN6	MISSENSE	c.1957C>G	p.Leu653Val	19%	VUS	chr3	1415619
YA4	MSS	7,7	DOCK3	MISSENSE	c.382C>A	p.Gln128Lys	31%	VUS	chr3	51101945

YA4	MSS	7,7	ABRAXAS1	MISSENSE	c.1214G>A	p.Arg405Gln	49%	VUS	chr4	84383638
YA4	MSS	7,7	TET2	MISSENSE	c.5166G>T	p.Met1722Ile	22%	VUS	chr4	106196770
YA4	MSS	7,7	LARP4B	MISSENSE	c.1505G>A	p.Arg502Gln	22%	VUS	chr10	866765
YA4	MSS	7,7	KMT2D	MISSENSE	c.8918T>C	p.Leu2973Pro	46%	VUS	chr12	49432221
YA4	MSS	7,7	PSMG2	MISSENSE	c.389A>G	p.Asn130Ser	21%	VUS	chr18	12718616
YA4	MSS	7,7	ASXL3	MISSENSE	c.5132C>T	p.Ser1711Phe	41%	VUS	chr18	31324944
YA5	MSS	4,77	APC	TRUNC	c.1600A>T	p.Lys534Ter	16%	Pathogenic	chr5	112163677
YA5	MSS	4,77	APC	TRUNC	c.4189G>T	p.Glu1397Ter	12%	Likely pathogenic	chr5	112175480
YA5	MSS	4,77	ATM	TRUNC	c.6222C>A	p.Cys2074Ter	21%	Pathogenic	chr11	108188123
YA5	MSS	4,77	SMAD4	MISSENSE	c.1082G>A	p.Arg361His	12%	Pathogenic	chr18	48591919
YA5	MSS	4,77	TCF7L2	MISSENSE	c.1154G>A	p.Gly385Glu	17%	VUS	chr10	114911636
YA5	MSS	4,77	FANCI	MISSENSE	c.2684C>T	p.Ser895Leu	36%	VUS	chr15	89843078
YA5	MSS	4,77	ZFHX3	MISSENSE	c.8558C>A	p.Ala2853Glu	16%	VUS	chr16	72828023
YA5	MSS	4,77	ZNF544	MISSENSE	c.2093G>A	p.Arg698Gln	49%	VUS	chr19	58774065
YA5	MSS	4,77	APIP	SPLICE	c.-1_1delinsT		52%	VUS	chr11	34937831
YA31	MSS	4,74	ASXL2	MISSENSE	c.4027T>C	p.Ser1343Pro	55%	VUS	chr2	25965179
YA31	MSS	4,74	ADAMTS12	MISSENSE	c.4565A>T	p.Glu1522Val	12%	VUS	chr5	33534979
YA31	MSS	4,74	LTO1	MISSENSE	c.98T>C	p.Leu33Ser	55%	VUS	chr11	69488042
YA31	MSS	4,74	B2M	MISSENSE	c.25G>C	p.Val9Leu	52%	VUS	chr15	45003769
YA31	MSS	4,74	BLM	MISSENSE	c.854A>C	p.Lys285Thr	18%	VUS	chr15	91295071
YA31	MSS	4,74	TP53	INFRAME	c.754_762de	p.Leu252_Ile254del	34%	Pathogenic	chr17	7577524
YA31	MSS	4,74	STK11	SPLICE	c.374+1A>G		33%	Pathogenic	chr19	1218500
YA7	MSS	14,51	APC	TRUNC	c.4216C>T	p.Gln1406Ter	35%	Pathogenic	chr5	112175507
YA7	MSS	14,51	SMAD4	TRUNC	c.742C>T	p.Gln248Ter	30%	Pathogenic	chr18	48584569
YA7	MSS	14,51	TP53	MISSENSE	c.734G>A	p.Gly245Asp	46%	Pathogenic	chr17	7577547
YA7	MSS	14,51	FANCI	TRUNC	c.3895C>T	p.Arg1299Ter	8%	Pathogenic	chr15	89858591
YA7	MSS	14,51	CTCF	TRUNC	c.1954_1955	p.Arg652ThrfsTer23	8%	Likely pathogenic	chr16	67670710
YA7	MSS	14,51	ACVR2A	TRUNC	c.1411_1438	p.Ser471ProfsTer6	78%	VUS	chr2	148684709
YA7	MSS	14,51	PDE1A	TRUNC	c.219_220in	p.Leu74IlefsTer35	11%	VUS	chr2	183105016
YA7	MSS	14,51	OR2G6	MISSENSE	c.903G>C	p.Leu301Phe	61%	VUS	chr1	248685850
YA7	MSS	14,51	ERAP2	MISSENSE	c.1666G>A	p.Gly556Arg	8%	VUS	chr5	96237303
YA7	MSS	14,51	CSMD1	MISSENSE	c.4889C>T	p.Thr1630Met	6%	VUS	chr8	3063121
YA7	MSS	14,51	CDKN2A	MISSENSE	c.335G>A	p.Arg112His	7%	VUS	chr9	21971023

YA7	MSS	14,51	PTCH1	MISSENSE	c.194T>C	p.Ile65Thr	7%	VUS	chr9	98270450
YA7	MSS	14,51	ARID5B	MISSENSE	c.3200A>T	p.Glu1067Val	40%	VUS	chr10	63852422
YA7	MSS	14,51	WT1	MISSENSE	c.149C>T	p.Ala50Val	7%	VUS	chr11	32456758
YA7	MSS	14,51	PARP2	MISSENSE	c.212G>C	p.Gly71Ala	19%	VUS	chr14	20813256
YA7	MSS	14,51	RAD51C	MISSENSE	c.712C>G	p.Leu238Val	19%	VUS	chr17	56787226
YA7	MSS	14,51	NOL4	MISSENSE	c.1216G>A	p.Glu406Lys	13%	VUS	chr18	31538223
YA7	MSS	14,51	BCL2	MISSENSE	c.55A>C	p.Ile19Leu	28%	VUS	chr18	60985845
YA7	MSS	14,51	JAK3	MISSENSE	c.521C>T	p.Ala174Val	6%	VUS	chr19	17953881
YA7	MSS	14,51	KMT2B	MISSENSE	c.1634G>A	p.Arg545Gln	6%	VUS	chr19	36211883
YA7	MSS	14,51	ZMYM5	TRUNC	c.376G>T	p.Glu126Ter	8%	VUS	chr13	20425945
YA8	MSS	20,26	CREBBP	SPLICE	c.3836+1G>A		5%	Pathogenic	chr16	3799627
YA8	MSS	20,26	KRAS	MISSENSE	c.38G>A	p.Gly13Asp	27%	Pathogenic	chr12	25398281
YA8	MSS	20,26	TP53	MISSENSE	c.743G>A	p.Arg248Gln	30%	Pathogenic	chr17	7577538
YA8	MSS	20,26	NOTCH3	MISSENSE	c.581G>A	p.Cys194Tyr	17%	Pathogenic	chr19	15302869
YA8	MSS	20,26	ATM	TRUNC	c.3549del	p.Asn1183LysfsTer3	21%	Likely pathogenic	chr11	108151868
YA8	MSS	20,26	REG3A	TRUNC	c.309G>A	p.Trp103Ter	23%	VUS	chr2	79385476
YA8	MSS	20,26	INPP4B	TRUNC	c.630T>A	p.Cys210Ter	16%	Likely pathogenic	chr4	143181703
YA8	MSS	20,26	JAK2	TRUNC	c.3034G>T	p.Glu1012Ter	21%	VUS	chr9	5090886
YA8	MSS	20,26	LRRC7	MISSENSE	c.122C>T	p.Thr41Ile	30%	VUS	chr1	70225895
YA8	MSS	20,26	DPYD	MISSENSE	c.1025A>G	p.Asp342Gly	59%	VUS	chr1	98058877
YA8	MSS	20,26	BRINP3	MISSENSE	c.413C>A	p.Ser138Tyr	46%	VUS	chr1	190250704
YA8	MSS	20,26	PP2D1	MISSENSE	c.725C>T	p.Thr242Ile	42%	VUS	chr3	20042887
YA8	MSS	20,26	FAT1	MISSENSE	c.4241C>T	p.Ala1414Val	9%	VUS	chr4	187554920
YA8	MSS	20,26	SFXN1	MISSENSE	c.367A>G	p.Ile123Val	46%	VUS	chr5	174937143
YA8	MSS	20,26	H3C2	MISSENSE	c.137C>T	p.Thr46Ile	22%	VUS	chr6	26032152
YA8	MSS	20,26	ARID1B	MISSENSE	c.365C>T	p.Ser122Phe	48%	VUS	chr6	157099179
YA8	MSS	20,26	CSMD3	MISSENSE	c.5462C>A	p.Pro1821Gln	17%	VUS	chr8	113421195
YA8	MSS	20,26	KMT2A	MISSENSE	c.6491C>T	p.Pro2164Leu	38%	VUS	chr11	118372558
YA8	MSS	20,26	KIAA1755	MISSENSE	c.1004G>A	p.Arg335Gln	41%	VUS	chr20	36869529
YA9	MSS	5,77	KRAS	MISSENSE	c.35G>T	p.Gly12Val	8%	Pathogenic	chr12	25398284
YA9	MSS	5,77	SMAD4	MISSENSE	c.1256G>T	p.Gly419Val	7%	Likely pathogenic	chr18	48593505
YA9	MSS	5,77	PRDM9	TRUNC	c.2331del	p.Cys778AlafsTer161	54%	VUS	chr5	23527528
YA9	MSS	5,77	C8B	MISSENSE	c.444C>G	p.Asp148Glu	48%	VUS	chr1	57420448

YA9	MSS	5,77	FOXL2	MISSENSE	c.211G>C	p.Ala71Pro	46%	VUS	chr3	138665354
YA9	MSS	5,77	FBXW7	MISSENSE	c.103C>T	p.Arg35Cys	44%	VUS	chr4	153332853
YA9	MSS	5,77	NOTCH1	MISSENSE	c.4336G>A	p.Glu1446Lys	52%	VUS	chr9	139400012
YA9	MSS	5,77	POLE	MISSENSE	c.479T>C	p.Val160Ala	51%	VUS	chr12	133256182
YA9	MSS	5,77	BRCA2	MISSENSE	c.4928T>C	p.Val1643Ala	46%	VUS	chr13	32913420
YA9	MSS	5,77	TP53	MISSENSE	c.631A>C	p.Thr211Pro	5%	VUS	chr17	7578218
YA9	MSS	5,77	MAP2K4	MISSENSE	c.1050G>C	p.Lys350Asn	8%	VUS	chr17	12043165
YA9	MSS	5,77	RAD51D	MISSENSE	c.818G>A	p.Arg273Gln	51%	VUS	chr17	33428365
YA10	i19-18378	60,45	KRAS	MISSENSE	c.436G>A	p.Ala146Thr	22%	Likely pathogenic	chr12	25378562
YA10	H-MSI	60,45	AKT1	MISSENSE	c.49G>A	p.Glu17Lys	41%	Pathogenic	chr14	105246551
YA10	H-MSI	60,45	MLH1	MISSENSE	c.350C>T	p.Thr117Met	48%	Pathogenic	chr3	37045935
YA10	i11-417	60,45	CTNNB1	MISSENSE	c.133T>G	p.Ser45Ala	50%	Likely pathogenic	chr3	41266136
YA10	H-MSI	60,45	RNF43	TRUNC	c.1976del	p.Gly659ValfsTer41	49%	Likely pathogenic	chr17	56435161
YA10	H-MSI	60,45	RIT1	MISSENSE	c.280G>A	p.Ala94Thr	24%	Pathogenic	chr1	155874530
YA10	H-MSI	60,45	OR6C68	TRUNC	c.154del	p.His52ThrfsTer2	34%	VUS	chr12	55886312
YA10	H-MSI	60,45	ZFHX3	TRUNC	c.2398del	p.Thr800ProfsTer24	25%	Likely pathogenic	chr16	72991647
YA10	H-MSI	60,45	KMT2B	TRUNC	c.5636del	p.Gly1879ValfsTer16	20%	Likely pathogenic	chr19	36223002
YA10	H-MSI	60,45	FBXW7	TRUNC	c.493dup	p.Thr165AsnfsTer13	26%	Likely pathogenic	chr4	153332463
YA10	H-MSI	60,45	FAT1	TRUNC	c.2213dup	p.Met739HisfsTer5	24%	Likely pathogenic	chr4	187628769
YA10	H-MSI	60,45	KMT2B	TRUNC	c.4235dup	p.Leu1413ProfsTer265	14%	Likely pathogenic	chr19	36218452
YA10	H-MSI	60,45	SDHB	MISSENSE	c.810G>A	p.Met270Ile	18%	VUS	chr1	17345409
YA10	H-MSI	60,45	ARID1A	MISSENSE	c.6327T>G	p.Asn2109Lys	16%	VUS	chr1	27106716
YA10	H-MSI	60,45	PIK3C2B	MISSENSE	c.2849T>C	p.Val950Ala	25%	VUS	chr1	204413214
YA10	H-MSI	60,45	VHL	MISSENSE	c.599G>A	p.Arg200Gln	23%	VUS	chr3	10191606
YA10	H-MSI	60,45	OR5H2	MISSENSE	c.751T>G	p.Leu251Val	7%	VUS	chr3	98002482
YA10	H-MSI	60,45	PIK3CA	MISSENSE	c.322C>T	p.Arg108Cys	20%	VUS	chr3	178916935
YA10	H-MSI	60,45	FBXW7	MISSENSE	c.1690C>T	p.Arg564Cys	11%	VUS	chr4	153245501
YA10	H-MSI	60,45	MAP3K4	MISSENSE	c.925G>A	p.Ala309Thr	8%	VUS	chr6	161470229
YA10	H-MSI	60,45	FAM120B	MISSENSE	c.1099G>A	p.Val367Met	16%	VUS	chr6	170627577
YA10	H-MSI	60,45	KMT2C	MISSENSE	c.13655G>T	p.Ser4552Ile	23%	VUS	chr7	151845357
YA10	H-MSI	60,45	KMT2C	MISSENSE	c.13418G>T	p.Gly4473Val	18%	VUS	chr7	151845594
YA10	H-MSI	60,45	NOTCH1	MISSENSE	c.5963T>G	p.Leu1988Arg	21%	VUS	chr9	139393683
YA10	H-MSI	60,45	GATA3	MISSENSE	c.629G>T	p.Gly210Val	39%	VUS	chr10	8100655

YA10	H-MSI	60,45	MKI67	MISSENSE	c.7793C>T	p.Thr2598Met	21%	VUS	chr10	129902311
YA10	H-MSI	60,45	WT1	MISSENSE	c.556G>A	p.Gly186Ser	8%	VUS	chr11	32456351
YA10	H-MSI	60,45	OR4C6	MISSENSE	c.160A>G	p.Arg54Gly	28%	VUS	chr11	55432802
YA10	H-MSI	60,45	OR10W1	MISSENSE	c.710C>T	p.Thr237Ile	22%	VUS	chr11	58034621
YA10	H-MSI	60,45	ATM	MISSENSE	c.473A>G	p.Glu158Gly	18%	VUS	chr11	108106538
YA10	H-MSI	60,45	TBX3	MISSENSE	c.760C>T	p.Pro254Ser	54%	VUS	chr12	115117354
YA10	H-MSI	60,45	TBX3	MISSENSE	c.492A>T	p.Lys164Asn	19%	VUS	chr12	115118849
YA10	H-MSI	60,45	SPTB	MISSENSE	c.3503T>C	p.Leu1168Pro	24%	VUS	chr14	65253180
YA10	H-MSI	60,45	FANCA	MISSENSE	c.837C>A	p.Asp279Glu	56%	VUS	chr16	89865630
YA10	H-MSI	60,45	FANCA	MISSENSE	c.290C>T	p.Ala97Val	24%	VUS	chr16	89877473
YA10	H-MSI	60,45	BRIP1	MISSENSE	c.1420C>T	p.Leu474Phe	21%	VUS	chr17	59871011
YA10	H-MSI	60,45	ABCA5	MISSENSE	c.2848G>A	p.Val950Met	31%	VUS	chr17	67267347
YA10	H-MSI	60,45	NOTCH3	MISSENSE	c.4063C>T	p.Leu1355Phe	10%	VUS	chr19	15288676
YA10	H-MSI	60,45	KMT2B	MISSENSE	c.7120C>T	p.Pro2374Ser	17%	VUS	chr19	36224734
YA10	H-MSI	60,45	ERCC2	MISSENSE	c.1940G>A	p.Arg647His	9%	VUS	chr19	45855870
YA10	H-MSI	60,45	PTPRT	MISSENSE	c.3946G>A	p.Gly1316Arg	17%	VUS	chr20	40714460
YA10	H-MSI	60,45	PTPRT	MISSENSE	c.3388G>T	p.Gly1130Trp	19%	VUS	chr20	40735494
YA10	H-MSI	60,45	TPTE	MISSENSE	c.439G>A	p.Val147Ile	34%	VUS	chr21	10951273
YA10	H-MSI	60,45	KRTAP21-1	MISSENSE	c.65C>A	p.Ser22Tyr	21%	VUS	chr21	32127632
YA10	H-MSI	60,45	ZRSR2	MISSENSE	c.1385G>A	p.Arg462Gln	43%	VUS	chrX	15841301
YA10	H-MSI	60,45	MAP3K15	MISSENSE	c.562G>A	p.Val188Met	51%	VUS	chrX	19482488
YA10	H-MSI	60,45	BCOR	MISSENSE	c.2785G>A	p.Ala929Thr	8%	VUS	chrX	39931814
YA10	H-MSI	60,45	KDM5C	MISSENSE	c.4541C>A	p.Thr1514Asn	9%	VUS	chrX	53222291
YA10	H-MSI	60,45	TAF1	MISSENSE	c.3266G>A	p.Arg1089His	7%	VUS	chrX	70613305
YA10	H-MSI	60,45	KMT2B	MISSENSE	c.303G>A	p.Trp101Ter	8%	Likely pathogenic	chr19	36209223
YA10	H-MSI	60,45	PTPRT	MISSENSE	c.3379G>T	p.Gly1127Trp	19%	VUS	chr20	40735494
YA11	MSS	6,79	APC	MISSENSE	c.2815A>T	p.Lys939Ter	18%	Pathogenic	chr5	112174106
YA11	MSS	6,79	APC	MISSENSE	c.4222G>T	p.Glu1408Ter	17%	Pathogenic	chr5	112175513
YA11	MSS	6,79	TP53	MISSENSE	c.817C>T	p.Arg273Cys	32%	Pathogenic	chr17	7577121
YA11	MSS	6,79	RAD50	MISSENSE	c.205G>A	p.Asp69Asn	3%	Likely pathogenic	chr5	131895051
YA11	MSS	6,79	CDC73	MISSENSE	c.932C>T	p.Thr311Ile	13%	VUS	chr1	193121534
YA11	MSS	6,79	FMN2	MISSENSE	c.4402C>T	p.Arg1468Cys	61%	VUS	chr1	240492733
YA11	MSS	6,79	BAP1	MISSENSE	c.1748C>T	p.Ser583Leu	4%	VUS	chr3	52437296

YA11	MSS	6,79	FAT1	MISSENSE	c.9697C>T	p.Arg3233Cys	6%	VUS	chr4	187532696
YA11	MSS	6,79	FAT1	MISSENSE	c.5506G>A	p.Asp1836Asn	53%	VUS	chr4	187542234
YA11	MSS	6,79	ROS1	MISSENSE	c.6115C>T	p.Arg2039Cys	35%	VUS	chr6	117638326
YA11	MSS	6,79	PIK3CG	MISSENSE	c.898G>A	p.Gly300Arg	6%	VUS	chr7	106508904
YA11	MSS	6,79	NBN	MISSENSE	c.1646A>C	p.Lys549Thr	19%	VUS	chr8	90965671
YA11	MSS	6,79	KMT2D	MISSENSE	c.2449C>G	p.Pro817Ala	78%	VUS	chr12	49445017
YA11	MSS	6,79	FANCA	MISSENSE	c.2267G>A	p.Arg756His	3%	VUS	chr16	89836623
YA11	MSS	6,79	PIK3R2	MISSENSE	c.1045G>A	p.Asp349Asn	4%	VUS	chr19	18273252
YA11	MSS	6,79	KMT2B	MISSENSE	c.2053G>A	p.Asp685Asn	51%	VUS	chr19	36212302
YA11	MSS	6,79	KDM5C	MISSENSE	c.1393G>A	p.Glu465Lys	3%	VUS	chrX	53240687
YA11	MSS	6,79	ARID1A	MISSENSE	c.3221G>A	p.Arg1074Gln	3%	VUS	chr1	27097632
YA11	MSS	6,79	PIM1	MISSENSE	c.736G>A	p.Glu246Lys	3%	VUS	chr6	37140900
YA12	MSS	18,46	APC	TRUNC	c.3871C>T	p.Gln1291Ter	8%	Pathogenic	chr5	112175162
YA12	MSS	18,46	KRAS	MISSENSE	c.35G>A	p.Gly12Asp	15%	Pathogenic	chr12	25398284
YA12	MSS	18,46	CREBBP	TRUNC	c.1270C>T	p.Arg424Ter	6%	Pathogenic	chr16	3842042
YA12	MSS	18,46	TP53	MISSENSE	c.700T>A	p.Tyr234Asn	10%	Likely pathogenic	chr17	7577581
YA12	MSS	18,46	SMAD2	TRUNC	c.388C>T	p.Arg130Ter	12%	Likely pathogenic	chr18	45395746
YA12	MSS	18,46	DCAF4L1	MISSENSE	c.373C>T	p.Arg125Trp	11%	VUS	chr4	41984182
YA12	MSS	18,46	ADAMTS12	MISSENSE	c.1247C>T	p.Pro416Leu	12%	VUS	chr5	33649746
YA12	MSS	18,46	KMT2C	MISSENSE	c.3320A>G	p.Asp1107Gly	13%	VUS	chr7	151921103
YA12	MSS	18,46	BRIP1	MISSENSE	c.550G>T	p.Asp184Tyr	52%	VUS	chr17	59924539
YA12	MSS	18,46	NOTCH1	MISSENSE	c.2983G>A	p.Gly995Ser	5%	VUS	chr9	139403510
YA13	MSS	91,22	POLE	MISSENSE	c.857C>G	p.Pro286Arg	24%	Pathogenic	chr12	133253184
YA13	MSS	91,22	KRAS	MISSENSE	c.38G>A	p.Gly13Asp	19%	Pathogenic	chr12	25398281
YA13	MSS	91,22	XPO1	MISSENSE	c.1711G>A	p.Glu571Lys	22%	Likely pathogenic	chr2	61719472
YA13	MSS	91,22	PIK3CA	MISSENSE	c.3073A>G	p.Thr1025Ala	24%	Pathogenic	chr3	178952018
YA13	MSS	91,22	ARID1A	TRUNC	c.5965C>T	p.Arg1989Ter	24%	Pathogenic	chr1	27106354
YA13	MSS	91,22	SMAD4	MISSENSE	c.1081C>T	p.Arg361Cys	18%	Pathogenic	chr18	48591918
YA13	MSS	91,22	APC	TRUNC	c.3093T>G	p.Tyr1031Ter	21%	Pathogenic	chr5	112174384
YA13	MSS	91,22	APC	TRUNC	c.4634C>A	p.Ser1545Ter	18%	Pathogenic	chr5	112175925
YA13	MSS	91,22	RAD50	TRUNC	c.3277C>T	p.Arg1093Ter	24%	Pathogenic	chr5	131953874
YA13	MSS	91,22	ATM	TRUNC	c.6352G>T	p.Glu2118Ter	21%	Likely pathogenic	chr11	108190685
YA13	MSS	91,22	ATM	TRUNC	c.742C>T	p.Arg248Ter	17%	Pathogenic	chr11	108115594

YA13	MSS	91,22	BRCA2	TRUNC	c.6385G>T	p.Glu2129Ter	21%	Pathogenic	chr13	32914877
YA13	MSS	91,22	EP300	TRUNC	c.1078dup	p.Glu360GlyfsTer51	21%	Likely pathogenic	chr22	41523659
YA13	MSS	91,22	BRCA2	MISSENSE	c.2647T>A	p.Phe883Ile	15%	VUS	chr13	32911139
YA13	MSS	91,22	BRCA2	MISSENSE	c.4334A>C	p.Lys1445Thr	25%	VUS	chr13	32912826
YA13	MSS	91,22	BRCA1	MISSENSE	c.1774A>C	p.Ser592Arg	22%	VUS	chr17	41245774
YA13	MSS	91,22	SMAD4	MISSENSE	c.358G>T	p.Asp120Tyr	19%	VUS	chr18	48575164
YA13	MSS	91,22	FBXW7	SPLICE	c.584+1G>A		18%	VUS	chr4	153271193
YA13	MSS	91,22	USP33	TRUNC	c.532G>T	p.Glu178Ter	13%	VUS	chr1	78201756
YA13	MSS	91,22	PIK3CB	TRUNC	c.1618G>T	p.Glu540Ter	21%	VUS	chr3	138417901
YA13	MSS	91,22	HCN1	TRUNC	c.226G>T	p.Glu76Ter	23%	VUS	chr5	45695970
YA13	MSS	91,22	PIK3R1	TRUNC	c.1669C>T	p.Arg557Ter	20%	Likely pathogenic	chr5	67591076
YA13	MSS	91,22	HDAC2	TRUNC	c.1294C>T	p.Arg432Ter	25%	VUS	chr6	114264599
YA13	MSS	91,22	LATS1	TRUNC	c.2209C>T	p.Arg737Ter	23%	Likely pathogenic	chr6	150001395
YA13	MSS	91,22	CSMD3	TRUNC	c.6745G>T	p.Glu2249Ter	15%	VUS	chr8	113349868
YA13	MSS	91,22	CSMD3	TRUNC	c.229G>T	p.Glu77Ter	31%	VUS	chr8	114326972
YA13	MSS	91,22	MAP2K4	TRUNC	c.955C>T	p.Gln319Ter	23%	Likely pathogenic	chr17	12032519
YA13	MSS	91,22	DSC3	TRUNC	c.1564G>T	p.Glu522Ter	25%	VUS	chr18	28588081
YA13	MSS	91,22	AMER1	TRUNC	c.1156G>T	p.Glu386Ter	30%	Likely pathogenic	chrX	63412011
YA13	MSS	91,22	ARID1A	MISSENSE	c.4693A>G	p.Thr1565Ala	25%	VUS	chr1	27101411
YA13	MSS	91,22	FUBP1	MISSENSE	c.871A>C	p.Ile291Leu	21%	VUS	chr1	78430008
YA13	MSS	91,22	PDE4DIP	MISSENSE	c.6143G>A	p.Arg2048Gln	10%	VUS	chr1	144865845
YA13	MSS	91,22	BRINP3	MISSENSE	c.2186G>A	p.Arg729His	16%	VUS	chr1	190067263
YA13	MSS	91,22	H3-3A	MISSENSE	c.268A>G	p.Ile90Val	16%	VUS	chr1	226253496
YA13	MSS	91,22	PARP1	MISSENSE	c.2692G>A	p.Ala898Thr	39%	VUS	chr1	226551738
YA13	MSS	91,22	OR2L13	MISSENSE	c.572C>A	p.Thr191Asn	14%	VUS	chr1	248263249
YA13	MSS	91,22	NRXN1	MISSENSE	c.2446C>T	p.Arg816Trp	22%	VUS	chr2	50733684
YA13	MSS	91,22	DNAH6	MISSENSE	c.11802A>C	p.Glu3934Asp	22%	VUS	chr2	85039527
YA13	MSS	91,22	CNTN6	MISSENSE	c.3007C>A	p.Gln1003Lys	12%	VUS	chr3	1445022
YA13	MSS	91,22	TOPAZ1	MISSENSE	c.629C>A	p.Ser210Tyr	18%	VUS	chr3	44284627
YA13	MSS	91,22	DOCK3	MISSENSE	c.1174C>T	p.Arg392Trp	21%	VUS	chr3	51251600
YA13	MSS	91,22	DOCK3	MISSENSE	c.1696C>T	p.Pro566Ser	20%	VUS	chr3	51266140
YA13	MSS	91,22	PBRM1	MISSENSE	c.1565G>A	p.Arg522Gln	22%	VUS	chr3	52651531
YA13	MSS	91,22	GABRB1	MISSENSE	c.538G>A	p.Glu180Lys	17%	VUS	chr4	47322220

YA13	MSS	91,22	KIT	MISSENSE	c.2630C>A	p.Ser877Tyr	21%	VUS	chr4	55602920
YA13	MSS	91,22	FBXW7	MISSENSE	c.744G>T	p.Glu248Asp	24%	VUS	chr4	153259071
YA13	MSS	91,22	FSTL5	MISSENSE	c.2181A>G	p.Ile727Met	13%	VUS	chr4	162307262
YA13	MSS	91,22	CBR4	MISSENSE	c.188T>C	p.Val63Ala	27%	VUS	chr4	169928862
YA13	MSS	91,22	FAT1	MISSENSE	c.340G>A	p.Asp114Asn	19%	VUS	chr4	187630642
YA13	MSS	91,22	PRDM9	MISSENSE	c.176A>C	p.Asn59Thr	13%	VUS	chr5	23509685
YA13	MSS	91,22	PRDM9	MISSENSE	c.1507A>C	p.Lys503Gln	18%	VUS	chr5	23526704
YA13	MSS	91,22	CDH10	MISSENSE	c.511G>A	p.Glu171Lys	18%	VUS	chr5	24537504
YA13	MSS	91,22	ADAMTS12	MISSENSE	c.4046C>T	p.Ala1349Val	9%	VUS	chr5	33561211
YA13	MSS	91,22	RAD50	MISSENSE	c.2371G>A	p.Asp791Asn	25%	VUS	chr5	131939155
YA13	MSS	91,22	FAT2	MISSENSE	c.3478G>A	p.Asp1160Asn	45%	VUS	chr5	150942982
YA13	MSS	91,22	TAP1	MISSENSE	c.1724G>A	p.Arg575His	15%	VUS	chr6	32816451
YA13	MSS	91,22	SIM1	MISSENSE	c.1000G>A	p.Asp334Asn	24%	VUS	chr6	100868833
YA13	MSS	91,22	MAP3K4	MISSENSE	c.1556G>A	p.Arg519Lys	19%	VUS	chr6	161470860
YA13	MSS	91,22	GALNT17	MISSENSE	c.1146G>T	p.Lys382Asn	19%	VUS	chr7	71130461
YA13	MSS	91,22	RUNDC3B	MISSENSE	c.767C>T	p.Pro256Leu	14%	VUS	chr7	87399983
YA13	MSS	91,22	RUNX1T1	MISSENSE	c.132G>T	p.Glu44Asp	27%	VUS	chr8	93107389
YA13	MSS	91,22	CSMD3	MISSENSE	c.182T>G	p.Phe61Cys	18%	VUS	chr8	114327019
YA13	MSS	91,22	MTAP	MISSENSE	c.75T>G	p.Ile25Met	15%	VUS	chr9	21815473
YA13	MSS	91,22	PPP6C	MISSENSE	c.538G>A	p.Glu180Lys	20%	VUS	chr9	127915943
YA13	MSS	91,22	NOTCH1	MISSENSE	c.6322G>A	p.Asp2108Asn	20%	VUS	chr9	139391869
YA13	MSS	91,22	TCF7L2	MISSENSE	c.1343G>A	p.Arg448Gln	21%	VUS	chr10	114920402
YA13	MSS	91,22	OR4C15	MISSENSE	c.250T>G	p.Leu84Val	20%	VUS	chr11	55322032
YA13	MSS	91,22	OR4C6	MISSENSE	c.312C>A	p.Phe104Leu	17%	VUS	chr11	55432954
YA13	MSS	91,22	CCND1	MISSENSE	c.440A>C	p.Lys147Thr	13%	VUS	chr11	69458625
YA13	MSS	91,22	ERBB3	MISSENSE	c.242G>A	p.Arg81Gln	25%	VUS	chr12	56478786
YA13	MSS	91,22	POLE	MISSENSE	c.3779C>T	p.Ala1260Val	39%	VUS	chr12	133226279
YA13	MSS	91,22	PARP4	MISSENSE	c.2011G>A	p.Glu671Lys	24%	VUS	chr13	25044067
YA13	MSS	91,22	TPP2	MISSENSE	c.2036C>T	p.Ser679Leu	25%	VUS	chr13	103295587
YA13	MSS	91,22	FANCM	MISSENSE	c.6123A>C	p.Gln2041His	54%	VUS	chr14	45669187
YA13	MSS	91,22	RAD51	MISSENSE	c.679G>A	p.Ala227Thr	21%	VUS	chr15	41021734
YA13	MSS	91,22	MAP2K4	MISSENSE	c.400C>T	p.Arg134Trp	19%	VUS	chr17	11998898
YA13	MSS	91,22	ATAD5	MISSENSE	c.3033G>T	p.Lys1011Asn	23%	VUS	chr17	29187527

YA13	MSS	91,22	KLK1	MISSENSE	c.452G>A	p.Ser151Asn	25%	VUS	chr19	51323454
YA13	MSS	91,22	CHEK2	MISSENSE	c.868A>C	p.Asn290His	21%	VUS	chr22	29099533
YA13	MSS	91,22	CHEK2	MISSENSE	c.208G>A	p.Glu70Lys	20%	VUS	chr22	29130502
YA13	MSS	91,22	EP300	MISSENSE	c.1739G>A	p.Arg580Gln	21%	VUS	chr22	41533773
YA13	MSS	91,22	EP300	MISSENSE	c.2386A>C	p.Met796Leu	17%	VUS	chr22	41545771
YA13	MSS	91,22	RTL4	MISSENSE	c.136G>T	p.Ala46Ser	36%	VUS	chrX	111698092
YA13	MSS	91,22	ATRX	MISSENSE	c.4749G>T	p.Lys1583Asn	46%	VUS	chrX	76890145
YA14	MSS	ND	APC	TRUNC	c.2547_254[illegible]	p.Asp849GlufsTer62	24%	Pathogenic	chr5	112173837
YA14	MSS	ND	APC	TRUNC	c.4473del	p.Phe1491LeufsTer16	67%	Pathogenic	chr5	112175761
YA14	MSS	ND	FANCD2	TRUNC	c.982C>T	p.Arg328Ter	13%	Pathogenic	chr3	10084827
YA14	MSS	ND	BCL6	TRUNC	c.808C>T	p.Arg270Ter	7%	VUS	chr3	187447385
YA14	MSS	ND	RPL22	MISSENSE	c.290G>A	p.Arg97His	8%	VUS	chr1	6246829
YA14	MSS	ND	SYNC	MISSENSE	c.1208G>A	p.Arg403Gln	8%	VUS	chr1	33160491
YA14	MSS	ND	RAD54L	MISSENSE	c.1637C>T	p.Thr546Met	7%	VUS	chr1	46739836
YA14	MSS	ND	TET2	MISSENSE	c.5695C>T	p.Arg1899Cys	7%	VUS	chr4	106197299
YA14	MSS	ND	PRDM9	MISSENSE	c.1084G>A	p.Gly362Ser	31%	VUS	chr5	23524576
YA14	MSS	ND	KMT2C	MISSENSE	c.219G>C	p.Glu73Asp	62%	VUS	chr7	152055703
YA14	MSS	ND	DLG5	MISSENSE	c.4950G>C	p.Gln1650His	47%	VUS	chr10	79566533
YA14	MSS	ND	DYNC1H1	MISSENSE	c.13117C>T	p.Arg4373Cys	7%	VUS	chr14	102514264
YA14	MSS	ND	THBS1	MISSENSE	c.1543C>T	p.Arg515Cys	7%	VUS	chr15	39880798
YA14	MSS	ND	CD276	MISSENSE	c.278C>T	p.Thr93Met	8%	VUS	chr15	73994794
YA14	MSS	ND	BLM	MISSENSE	c.646G>A	p.Glu216Lys	9%	VUS	chr15	91293144
YA14	MSS	ND	RPTOR	MISSENSE	c.2353G>A	p.Asp785Asn	6%	VUS	chr17	78867617
YA14	MSS	ND	CIC	MISSENSE	c.3501G>T	p.Glu1167Asp	17%	VUS	chr19	42791970
YA14	MSS	ND	KDM5C	MISSENSE	c.3494G>A	p.Arg1165His	7%	VUS	chrX	53223865
YA14	MSS	ND	KDM5C	MISSENSE	c.3019C>T	p.Arg1007Cys	11%	VUS	chrX	53225199
YA14	MSS	ND	AMER1	MISSENSE	c.622G>A	p.Val208Met	7%	VUS	chrX	63412545
YA15	MSS	ND	APC	TRUNC	c.3927_393[illegible]	p.Glu1309AspfsTer4	33%	Pathogenic	chr5	112175212
YA15	MSS	ND	PIK3CA	MISSENSE	c.1093G>A	p.Glu365Lys	8%	Pathogenic	chr3	178922324
YA15	MSS	ND	TP53	MISSENSE	c.757A>C	p.Thr253Pro	37%	Likely pathogenic	chr17	7577524
YA15	MSS	ND	RAD54L	MISSENSE	c.1655G>A	p.Arg552Gln	7%	VUS	chr1	46739854
YA15	MSS	ND	FANCD2	MISSENSE	c.1223G>A	p.Arg408Gln	9%	VUS	chr3	10088352
YA15	MSS	ND	FANCD2	MISSENSE	c.3961C>T	p.Arg1321Trp	8%	VUS	chr3	10136045

YA15	MSS	ND	PBRM1	MISSENSE	c.3209T>G	p.Phe1070Cys	17%	VUS	chr3	52620619
YA15	MSS	ND	PBRM1	MISSENSE	c.1301G>A	p.Arg434Gln	8%	VUS	chr3	52668618
YA15	MSS	ND	FAT1	MISSENSE	c.2693G>A	p.Arg898Lys	52%	VUS	chr4	187628289
YA15	MSS	ND	FLT4	MISSENSE	c.1345G>A	p.Gly449Arg	12%	VUS	chr5	180052945
YA15	MSS	ND	FLT4	MISSENSE	c.959G>A	p.Arg320Gln	7%	VUS	chr5	180056285
YA15	MSS	ND	TNFAIP3	MISSENSE	c.1186A>T	p.Met396Leu	55%	VUS	chr6	138199768
YA15	MSS	ND	ARID1B	MISSENSE	c.2105C>A	p.Pro702Gln	29%	VUS	chr6	157405902
YA15	MSS	ND	ARID1B	MISSENSE	c.5794G>A	p.Ala1932Thr	6%	VUS	chr6	157527949
YA15	MSS	ND	MET	MISSENSE	c.3497G>A	p.Arg1166Gln	8%	VUS	chr7	116418932
YA15	MSS	ND	VWA3A	MISSENSE	c.2713C>T	p.Pro905Ser	59%	VUS	chr16	22155688
YA15	MSS	ND	DSG2	MISSENSE	c.872C>T	p.Thr291Met	8%	VUS	chr18	29104709
YA15	MSS	ND	KMT2B	MISSENSE	c.1613G>A	p.Arg538His	7%	VUS	chr19	36211862
YA16	MSS	8,66	APC	TRUNC	c.4393_4394	p.Ser1465TrpfsTer3	32%	Pathogenic	chr5	112175676
YA16	MSS	8,66	APC	SPLICE	c.1312+1G>A		28%	Pathogenic	chr5	112155042
YA16	MSS	8,66	KRAS	MISSENSE	c.35G>A	p.Gly12Asp	33%	Pathogenic	chr12	25398284
YA16	MSS	8,66	AMER1	TRUNC	c.1057C>T	p.Arg353Ter	68%	Pathogenic	chrX	63412110
YA16	MSS	8,66	TP53	MISSENSE	c.524G>A	p.Arg175His	40%	Pathogenic	chr17	7578406
YA16	MSS	8,66	PTEN	TRUNC	c.128_131du	p.Val45ArgfsTer8	42%	Likely pathogenic	chr10	89653829
YA16	MSS	8,66	PTEN	MISSENSE	c.4022T>G	p.Met1341Arg	42%	VUS	chr10	89653830
YA16	MSS	8,66	PBRM1	MISSENSE	c.4178T>G	p.Met1393Arg	3%	VUS	chr3	52595893
YA16	MSS	8,66	PABPC4L	MISSENSE	c.1100C>G	p.Ala367Gly	21%	VUS	chr4	135121075
YA16	MSS	8,66	CSMD3	MISSENSE	c.8266A>T	p.Ile2756Phe	6%	VUS	chr8	113316950
YA16	MSS	8,66	OR5L2	MISSENSE	c.203T>G	p.Phe68Cys	15%	VUS	chr11	55594897
YA16	MSS	8,66	SLCO1B3	MISSENSE	c.542G>T	p.Arg181Leu	47%	VUS	chr12	21015406
YA16	MSS	8,66	TRHDE	MISSENSE	c.1893A>C	p.Glu631Asp	3%	VUS	chr12	72956671
YA16	MSS	8,66	ERBB2	MISSENSE	c.2916G>C	p.Leu972Phe	13%	VUS	chr17	37882858
YA16	MSS	8,66	ZNF407	MISSENSE	c.2870T>C	p.Leu957Ser	47%	VUS	chr18	72345845
YA16	MSS	8,66	KMT2B	MISSENSE	c.7471C>T	p.Arg2491Cys	9%	VUS	chr19	36228085
YA16	MSS	8,66	FAT1	TRUNC	c.13138+2T>G		5%	VUS	chr4	187516841
YA17	H-MSI	41,26	SMARCB1	MISSENSE	c.1121G>A	p.Arg374Gln	28%	Pathogenic	chr22	24176330
YA17	H-MSI	41,26	RNF43	TRUNC	c.1976del	p.Gly659ValfsTer41	9%	Likely pathogenic	chr17	56435161
YA17	H-MSI	41,26	RNF43	TRUNC	c.575del	p.Pro192ArgfsTer11	23%	Likely pathogenic	chr17	56440643
YA17	H-MSI	41,26	RAD50	TRUNC	c.1208_1209	p.Gln404AspfsTer12	17%	Pathogenic	chr5	131924527

YA17	H-MSI	41,26	RNF43	TRUNC	c.394C>T	p.Arg132Ter	27%	Pathogenic	chr17	56440943
YA17	H-MSI	41,26	MSH2	TRUNC	c.1216C>T	p.Arg406Ter	52%	Pathogenic	chr2	47657020
YA17	H-MSI	41,26	MSH2	TRUNC	c.2131C>T	p.Arg711Ter	23%	Pathogenic	chr2	47703631
YA17	H-MSI	41,26	TPP2	TRUNC	c.3076C>T	p.Arg1026Ter	28%	VUS	chr13	103309529
YA17	H-MSI	41,26	TP53	MISSENSE	c.469G>A	p.Val157Ile	24%	VUS	chr17	7578461
YA17	H-MSI	41,26	RTEL1	TRUNC	c.2021delC	p.Pro674HisfsTer17	23%	VUS	chr20	62320919
YA17	H-MSI	41,26	ARID1A	TRUNC	c.3344del	p.Pro1115GlnfsTer46	26%	Likely pathogenic	chr1	27097751
YA17	H-MSI	41,26	CTLA4	TRUNC	c.598del	p.Val200SerfsTer3	12%	VUS	chr2	204737458
YA17	H-MSI	41,26	FAT1	TRUNC	c.8799del	p.Gly2934ValfsTer3	14%	Likely pathogenic	chr4	187538942
YA17	H-MSI	41,26	OR8H2	TRUNC	c.896del	p.Asn299MetfsTer16	26%	VUS	chr11	55873410
YA17	H-MSI	41,26	PUS3	TRUNC	c.104del	p.Asn35IlefsTer31	27%	VUS	chr11	125766076
YA17	H-MSI	41,26	JAK3	TRUNC	c.251del	p.Pro84ArgfsTer63	30%	VUS	chr19	17954643
YA17	H-MSI	41,26	ERCC2	TRUNC	c.1780del	p.Ala594ProfsTer115	23%	Likely pathogenic	chr19	45856392
YA17	H-MSI	41,26	KDM5C	TRUNC	c.589del	p.Leu197TyrfsTer37	28%	Likely pathogenic	chrX	53246393
YA17	H-MSI	41,26	FAT1	TRUNC	c.6112_6113	p.Pro2038LeufsTer6	17%	Likely pathogenic	chr4	187541630
YA17	H-MSI	41,26	ZFHX3	TRUNC	c.10191dup	p.Ala3398SerfsTer33	24%	Likely pathogenic	chr16	72821984
YA17	H-MSI	41,26	CNTNAP4	TRUNC	c.1144_1145	p.Ser382PhefsTer7	13%	Likely pathogenic	chr16	76495881
YA17	H-MSI	41,26	SPEN	MISSENSE	c.5872C>T	p.Arg1958Trp	14%	VUS	chr1	16258607
YA17	H-MSI	41,26	EPHA2	MISSENSE	c.307C>T	p.Arg103Cys	24%	VUS	chr1	16475389
YA17	H-MSI	41,26	ID3	MISSENSE	c.166C>T	p.Pro56Ser	9%	VUS	chr1	23885752
YA17	H-MSI	41,26	MYCL	MISSENSE	c.925C>T	p.Pro309Ser	9%	VUS	chr1	40363214
YA17	H-MSI	41,26	OR2L8	MISSENSE	c.719G>A	p.Cys240Tyr	18%	VUS	chr1	248112878
YA17	H-MSI	41,26	EPCAM	MISSENSE	c.518G>A	p.Arg173His	13%	VUS	chr2	47604179
YA17	H-MSI	41,26	INPP4A	MISSENSE	c.2672G>A	p.Arg891Gln	24%	VUS	chr2	99193477
YA17	H-MSI	41,26	BCL6	MISSENSE	c.572C>T	p.Pro191Leu	10%	VUS	chr3	187447621
YA17	H-MSI	41,26	GRID2	MISSENSE	c.1886C>T	p.Ala629Val	10%	VUS	chr4	94411817
YA17	H-MSI	41,26	INPP4B	MISSENSE	c.35G>A	p.Gly12Glu	22%	VUS	chr4	143352378
YA17	H-MSI	41,26	TERT	MISSENSE	c.1844C>T	p.Ala615Val	12%	VUS	chr5	1280379
YA17	H-MSI	41,26	IL7R	MISSENSE	c.1135G>A	p.Ala379Thr	13%	VUS	chr5	35876343
YA17	H-MSI	41,26	HCN1	MISSENSE	c.1679G>A	p.Arg560His	17%	VUS	chr5	45267295
YA17	H-MSI	41,26	MAP3K1	MISSENSE	c.2557C>T	p.Arg853Cys	22%	VUS	chr5	56177584
YA17	H-MSI	41,26	ERAP2	MISSENSE	c.2572C>G	p.Leu858Val	51%	VUS	chr5	96249076
YA17	H-MSI	41,26	APC	MISSENSE	c.4978C>A	p.Leu1660Ile	23%	VUS	chr5	112176269

YA17	H-MSI	41,26	HLA-A	MISSENSE	c.499A>G	p.Thr167Ala	6%	VUS	chr6	29911176
YA17	H-MSI	41,26	TAP2	MISSENSE	c.1742A>G	p.Gln581Arg	52%	VUS	chr6	32797760
YA17	H-MSI	41,26	FANCE	MISSENSE	c.436G>T	p.Val146Leu	40%	VUS	chr6	35423711
YA17	H-MSI	41,26	ARID1B	MISSENSE	c.2960C>T	p.Ala987Val	10%	VUS	chr6	157488293
YA17	H-MSI	41,26	CUL1	MISSENSE	c.1411G>A	p.Ala471Thr	28%	VUS	chr7	148484144
YA17	H-MSI	41,26	CSMD3	MISSENSE	c.4354A>G	p.Ile1452Val	21%	VUS	chr8	113564830
YA17	H-MSI	41,26	OR13J1	MISSENSE	c.364C>T	p.Arg122Cys	36%	VUS	chr9	35870035
YA17	H-MSI	41,26	ABL1	MISSENSE	c.658C>T	p.Arg220Cys	10%	VUS	chr9	133738258
YA17	H-MSI	41,26	NOTCH1	MISSENSE	c.5522G>A	p.Arg1841Gln	6%	VUS	chr9	139396316
YA17	H-MSI	41,26	LARP4B	MISSENSE	c.578C>T	p.Thr193Met	22%	VUS	chr10	888940
YA17	H-MSI	41,26	FAS	MISSENSE	c.188G>A	p.Cys63Tyr	8%	VUS	chr10	90762943
YA17	H-MSI	41,26	TCF7L2	MISSENSE	c.1778C>T	p.Pro593Leu	21%	VUS	chr10	114925700
YA17	H-MSI	41,26	TRHDE	MISSENSE	c.3014G>A	p.Arg1005His	10%	VUS	chr12	73056914
YA17	H-MSI	41,26	ANO4	MISSENSE	c.613C>A	p.Gln205Lys	27%	VUS	chr12	101381432
YA17	H-MSI	41,26	BRCA2	MISSENSE	c.9188C>T	p.Pro3063Leu	21%	VUS	chr13	32954214
YA17	H-MSI	41,26	OR4M1	MISSENSE	c.193A>G	p.Asn65Asp	35%	VUS	chr14	20248674
YA17	H-MSI	41,26	SFTA3	MISSENSE	c.8C>T	p.Ala3Val	19%	VUS	chr14	36982351
YA17	H-MSI	41,26	FANCI	MISSENSE	c.1408G>A	p.Ala470Thr	13%	VUS	chr15	89824427
YA17	H-MSI	41,26	IDH2	MISSENSE	c.1247C>T	p.Ala416Val	22%	VUS	chr15	90628072
YA17	H-MSI	41,26	IDH2	MISSENSE	c.1207G>A	p.Val403Met	24%	VUS	chr15	90628112
YA17	H-MSI	41,26	TSC2	MISSENSE	c.4966G>A	p.Asp1656Asn	13%	VUS	chr16	2136849
YA17	H-MSI	41,26	CREBBP	MISSENSE	c.6166G>A	p.Val2056Met	10%	VUS	chr16	3778882
YA17	H-MSI	41,26	ZFHX3	MISSENSE	c.8198G>A	p.Arg2733His	20%	VUS	chr16	72828383
YA17	H-MSI	41,26	ZFHX3	MISSENSE	c.2347G>A	p.Ala783Thr	25%	VUS	chr16	72991698
YA17	H-MSI	41,26	RPTOR	MISSENSE	c.3196C>T	p.Arg1066Trp	20%	VUS	chr17	78921082
YA17	H-MSI	41,26	ANKRD12	MISSENSE	c.2695G>A	p.Asp899Asn	14%	VUS	chr18	9255960
YA17	H-MSI	41,26	KMT2B	MISSENSE	c.2848C>T	p.Arg950Trp	8%	VUS	chr19	36214022
YA17	H-MSI	41,26	ZNF320	MISSENSE	c.1177G>A	p.Val393Ile	11%	VUS	chr19	53384202
YA17	H-MSI	41,26	RALGAPA2	MISSENSE	c.1405T>G	p.Ser469Ala	24%	VUS	chr20	20600055
YA17	H-MSI	41,26	RUNX1	MISSENSE	c.833C>T	p.Pro278Leu	10%	VUS	chr21	36171732
YA17	H-MSI	41,26	NF2	MISSENSE	c.155G>A	p.Arg52Gln	9%	VUS	chr22	30032780
YA17	H-MSI	41,26	DDX3X	MISSENSE	c.109C>T	p.Arg37Cys	47%	VUS	chrX	41198294
YA17	H-MSI	41,26	PDIA3	SPLICE	c.1028+1G>A		18%	VUS	chr15	44059109

YA17	H-MSI	41,26	BRIP1	SPLICE	c.2379+2T>C		25%	VUS	chr17	59820372
YA17	H-MSI	41,26	ATRX	SPLICE	c.4214+2T>C		38%	VUS	chrX	76912048
YA18	MSS	1,91	APC	TRUNC	c.3669_3680	p.Asn1224AlafsTer12	54%	Pathogenic	chr5	112174960
YA18	MSS	1,91	TCF7L2	TRUNC	c.233_234de	p.Lys78IlefsTer18	27%	Likely pathogenic	chr10	114711008
YA18	MSS	1,91	SOX9	TRUNC	c.811del	p.Arg271AlafsTer8	21%	Likely pathogenic	chr17	70119808
YA18	MSS	1,91	PARP3	MISSENSE	c.1324C>G	p.His442Asp	45%	VUS	chr3	51981782
YA18	MSS	1,91	LARP1B	MISSENSE	c.1904G>C	p.Arg635Thr	4%	VUS	chr4	129100568
YA18	MSS	1,91	ATM	MISSENSE	c.5089A>G	p.Thr1697Ala	59%	VUS	chr11	108170524
YA18	MSS	1,91	TP53	MISSENSE	c.470T>G	p.Val157Gly	40%	VUS	chr17	7578460
YA19	MSS	8,51	KRAS	MISSENSE	c.351A>T	p.Lys117Asn	47%	Pathogenic	chr12	25378647
YA19	MSS	8,51	PIK3CA	MISSENSE	c.1357G>A	p.Glu453Lys	13%	Pathogenic	chr3	178928079
YA19	MSS	8,51	TP53	MISSENSE	c.844C>T	p.Arg282Trp	11%	Pathogenic	chr17	7577094
YA19	MSS	8,51	RBM10	TRUNC	c.883C>T	p.Arg295Ter	15%	Likely pathogenic	chrX	47038526
YA19	MSS	8,51	ARID1A	SPLICE	c.1351-1G>A		11%	Likely pathogenic	chr1	27057642
YA19	MSS	8,51	SMAD4	MISSENSE	c.1611C>G	p.Asp537Glu	13%	Likely pathogenic	chr18	48604789
YA19	MSS	8,51	MSH3	MISSENSE	c.1320C>G	p.Ile440Met	58%	VUS	chr5	79974892
YA19	MSS	8,51	ABCB1	MISSENSE	c.724A>G	p.Lys242Glu	8%	VUS	chr7	87190682
YA19	MSS	8,51	DSCC1	MISSENSE	c.1099A>G	p.Ile367Val	12%	VUS	chr8	120847216
YA19	MSS	8,51	LARP4B	MISSENSE	c.1187C>T	p.Ala396Val	44%	VUS	chr10	871749
YA19	MSS	8,51	PTPN11	MISSENSE	c.1226G>C	p.Gly409Ala	47%	VUS	chr12	112924280
YA19	MSS	8,51	MGA	MISSENSE	c.4015A>G	p.Ile1339Val	41%	VUS	chr15	42028477
YA19	MSS	8,51	RUNX1	MISSENSE	c.849G>C	p.Gln283His	46%	VUS	chr21	36171716
YA20	MSS	4,81	PIK3CA	MISSENSE	c.1633G>A	p.Glu545Lys	25%	Pathogenic	chr3	178936091
YA20	MSS	4,81	APC	TRUNC	c.2255_2256	p.Leu752ProfsTer3	24%	Likely pathogenic	chr5	112173546
YA20	MSS	4,81	APC	TRUNC	c.4479_4481	p.Ser1495ValfsTer12	27%	Likely pathogenic	chr5	112175770
YA20	MSS	4,81	ARID1B	MISSENSE	c.4718C>T	p.Thr1573Ile	53%	VUS	chr6	157522326
YA20	MSS	4,81	RUNX1T1	MISSENSE	c.169A>C	p.Asn57His	53%	VUS	chr8	93107352
YA20	MSS	4,81	CYP2C9	MISSENSE	c.1198G>A	p.Glu400Lys	53%	VUS	chr10	96745838
YA20	MSS	4,81	KRAS	MISSENSE	c.34G>A	p.Gly12Ser	43%	Pathogenic	chr12	25398285
YA20	MSS	4,81	HNF1A	MISSENSE	c.1741G>T	p.Ala581Ser	4%	VUS	chr12	121437403
YA20	MSS	4,81	IGF1R	MISSENSE	c.1816A>T	p.Thr606Ser	49%	VUS	chr15	99456499
YA20	MSS	4,81	TP53	SPLICE	c.783-1G>T		38%	Likely pathogenic	chr17	7577156
YA20	MSS	4,81	DSG2	INFRAME	c.847_849de	p.Glu283del	26%	VUS	chr18	29104684

YA20	MSS	4,81	JAK3	MISSENSE	c.934G>A	p.Ala312Thr	4%	VUS	chr19	17952499
YA21	MSS	0	TMEM123	MISSENSE	c.220C>T	p.Pro74Ser	49%	VUS	chr11	102272875
YA21	MSS	0	TPTE	MISSENSE	c.1187A>G	p.Tyr396Cys	23%	VUS	chr21	10916459
YA22	MSS	6,63	TP53	SPLICE	c.993+1del		65%	Pathogenic	chr17	7576852
YA22	MSS	6,63	AR	MISSENSE	c.2440T>G	p.Phe814Val	22%	VUS	chrX	66941796
YA22	MSS	6,63	SETD2	MISSENSE	c.7606G>A	p.Val2536Met	14%	VUS	chr3	47058672
YA22	MSS	6,63	TNFAIP3	MISSENSE	c.2165A>G	p.Lys722Arg	41%	VUS	chr6	138202248
YA22	MSS	6,63	GLI3	MISSENSE	c.3028C>T	p.Arg1010Trp	14%	VUS	chr7	42005643
YA22	MSS	6,63	FANCG	MISSENSE	c.770G>A	p.Arg257His	56%	VUS	chr9	35076975
YA22	MSS	6,63	KMT2A	MISSENSE	c.5629G>A	p.Ala1877Thr	25%	VUS	chr11	118367047
YA22	MSS	6,63	ETV6	MISSENSE	c.604C>T	p.Arg202Trp	37%	VUS	chr12	12022498
YA22	MSS	6,63	ETV6	MISSENSE	c.1186A>G	p.Arg396Gly	40%	VUS	chr12	12038893
YA22	MSS	6,63	NOTCH3	MISSENSE	c.4072T>G	p.Phe1358Val	12%	VUS	chr19	15288667
YA22	MSS	6,63	AXIN1	SPLICE	c.2294+1G>A		23%	VUS	chr16	341189
YA23	MSS	2,84	NCAPG	MISSENSE	c.862T>G	p.Leu288Val	51%	VUS	chr4	17818970
YA23	MSS	2,84	FAT1	MISSENSE	c.1313T>G	p.Val438Gly	51%	VUS	chr4	187629669
YA23	MSS	2,84	NBN	MISSENSE	c.839C>T	p.Thr280Ile	54%	VUS	chr8	90982649
YA23	MSS	2,84	KRAS	MISSENSE	c.34G>T	p.Gly12Cys	27%	Pathogenic	chr12	25398285
YA23	MSS	2,84	TRHDE	MISSENSE	c.2488C>G	p.His830Asp	18%	VUS	chr12	73015479
YA23	MSS	2,84	CREBBP	MISSENSE	c.2686G>A	p.Gly896Arg	28%	VUS	chr16	3820765
YA23	MSS	2,84	TP53	MISSENSE	c.742C>T	p.Arg248Trp	44%	Pathogenic	chr17	7577539
YA23	MSS	2,84	PRKAR1A	MISSENSE	c.38G>A	p.Arg13His	3%	VUS	chr17	66511578
YA24	MSS	16	FBXW7	MISSENSE	c.1393C>T	p.Arg465Cys	27%	Likely pathogenic	chr4	153249385
YA24	MSS	16	PTEN	TRUNC	c.195C>G	p.Tyr65Ter	16%	Pathogenic	chr10	89685300
YA24	MSS	16	FANCF	MISSENSE	c.720G>C	p.Trp240Cys	42%	VUS	chr11	22646637
YA24	MSS	16	PARP4	MISSENSE	c.3127A>G	p.Met1043Val	26%	VUS	chr13	25021312
YA24	MSS	16	GPS2	MISSENSE	c.920G>A	p.Ser307Asn	15%	VUS	chr17	7216139
YA24	MSS	16	TP53	TRUNC	c.851_852de	p.Thr284ArgfsTer21	71%	Pathogenic	chr17	7577086
YA24	MSS	16	BRCA1	MISSENSE	c.4054G>A	p.Glu1352Lys	9%	VUS	chr17	41243494
YA24	MSS	16	NOTCH3	MISSENSE	c.1971G>T	p.Glu657Asp	42%	VUS	chr19	15296471
YA24	MSS	16	PTPRT	MISSENSE	c.4109G>A	p.Arg1370His	14%	VUS	chr20	40713406
YA24	MSS	16	TPTE	MISSENSE	c.1578T>G	p.Ile526Met	27%	VUS	chr21	10906983
YA24	MSS	16	ZNF217	MISSENSE	c.3125G>A	p.Arg1042Gln	3%	VUS	chr20	52188305

YA24	MSS	16	ADAMTS12	TRUNC	c.1468C>T	p.Gln490Ter	7%	VUS	chr5	33648938
YA25	MSS	7,08	PIK3CA	MISSENSE	c.1633G>A	p.Glu545Lys	13%	Pathogenic		
YA25	MSS	7,08	APC	TRUNC	c.4285C>T	p.Gln1429Ter	7%	Likely pathogenic		
YA25	MSS	7,08	BRAF	MISSENSE	c.1799_1800	p.Val600Glu	15%	Pathogenic		
YA25	MSS	7,08	TP53	SPLICE	c.782+1G>T		25%	Likely pathogenic		
YA25	MSS	7,08	TP53	MISSENSE	c.472C>T	p.Arg158Cys	39%	Likely pathogenic		
YA25	MSS	7,08	SRSF4	MISSENSE	c.1097G>A	p.Ser366Asn	60%	VUS		
YA25	MSS	7,08	NOTCH2	MISSENSE	c.6703A>C	p.Ser2235Arg	50%	VUS		
YA25	MSS	7,08	MTERF4	MISSENSE	c.553G>C	p.Glu185Gln	53%	VUS		
YA25	MSS	7,08	SETD2	MISSENSE	c.5998G>A	p.Asp2000Asn	47%	VUS		
YA25	MSS	7,08	PIK3R1	MISSENSE	c.385C>A	p.Pro129Thr	13%	VUS		
YA25	MSS	7,08	ERAP2	MISSENSE	c.2186A>T	p.Tyr729Phe	7%	VUS		
YA25	MSS	7,08	APC	MISSENSE	c.7477C>A	p.Leu2493Ile	14%	VUS		
YA25	MSS	7,08	FANCM	MISSENSE	c.2369T>G	p.Val790Gly	49%	VUS		
YA26	MSS	3,81	ARID1A	MISSENSE	c.1641C>A	p.Ser547Arg	7%	VUS	chr1	27057933
YA26	MSS	3,81	C8B	MISSENSE	c.142G>T	p.Ala48Ser	14%	VUS	chr1	57425800
YA26	MSS	3,81	BRINP3	MISSENSE	c.203G>A	p.Arg68Gln	18%	VUS	chr1	190423818
YA26	MSS	3,81	FASTKD1	MISSENSE	c.1402T>C	p.Tyr468His	57%	VUS	chr2	170403027
YA26	MSS	3,81	TTC23L	MISSENSE	c.64_65delir	p.His22Cys	46%	VUS	chr5	34840840
YA26	MSS	3,81	ATM	MISSENSE	c.2062G>A	p.Glu688Lys	43%	VUS	chr11	108124704
YA26	MSS	3,81	ATM	TRUNC	c.8067_8071	p.Phe2690LeufsTer26	11%	Likely pathogenic	chr11	108205752
YA26	MSS	3,81	ATM	MISSENSE	c.8774G>C	p.Gly2925Ala	24%	VUS	chr11	108224595
YA26	MSS	3,81	KRAS	MISSENSE	c.183A>C	p.Gln61His	13%	Pathogenic	chr12	25380275
YA26	MSS	3,81	CYLD	MISSENSE	c.1711G>A	p.Val571Ile	49%	VUS	chr16	50816262
YA26	MSS	3,81	DSC3	MISSENSE	c.517A>C	p.Ile173Leu	51%	VUS	chr18	28605839
YA26	MSS	3,81	EP300	MISSENSE	c.6226G>A	p.Ala2076Thr	17%	VUS	chr22	41573941
YA27	MSS	1,81	EPHA2	MISSENSE	c.2585C>T	p.Pro862Leu	43%	VUS	chr1	16456805
YA27	MSS	1,81	ATR	MISSENSE	c.5257A>G	p.Ile1753Val	49%	VUS	chr3	142222235
YA27	MSS	1,81	APC	TRUNC	c.4025dup	p.Leu1342PhefsTer12	18%	Pathogenic	chr5	112175314
YA27	MSS	1,81	TCF7L2	TRUNC	c.115_116de	p.Arg39GlyfsTer4	12%	Likely pathogenic	chr10	114710630
YA27	MSS	1,81	CUL4A	MISSENSE	c.1292G>A	p.Arg431Gln	47%	VUS	chr13	113898787
YA27	MSS	1,81	MGA	MISSENSE	c.79A>G	p.Ile27Val	55%	VUS	chr15	41961171
YA27	MSS	1,81	PDIA3	MISSENSE	c.738C>G	p.His246Gln	41%	VUS	chr15	44058103

YA27	MSS	1,81	TP53	TRUNC	c.586C>T	p.Arg196Ter	27%	Pathogenic	chr17	7578263
YA27	MSS	1,81	ZNF624	TRUNC	c.412C>T	p.Arg138Ter	16%	VUS	chr17	16527788
YA27	MSS	1,81	CYP2D6	MISSENSE	c.839A>G	p.Glu280Gly	53%	VUS	chr22	42524180
YA28	MSS	6,68	APC	TRUNC	c.4332_4333	p.Thr1445LysfsTer29	25%	Likely pathogenic	chr5	112175621
YA28	MSS	6,68	KRAS	MISSENSE	c.35G>A	p.Gly12Asp	21%	Pathogenic	chr12	25398284
YA28	MSS	6,68	TP53	MISSENSE	c.524G>A	p.Arg175His	43%	Pathogenic	chr17	7578406
YA28	MSS	6,68	ASXL1	TRUNC	c.3202C>T	p.Arg1068Ter	20%	Likely pathogenic	chr20	31023717
YA28	MSS	6,68	OR2M3	MISSENSE	c.13A>C	p.Asn5His	20%	VUS	chr1	248366382
YA28	MSS	6,68	AKAP13	MISSENSE	c.2593C>T	p.Leu865Phe	47%	VUS	chr15	86123892
YA28	MSS	6,68	TPTE	MISSENSE	c.1578T>G	p.Ile526Met	8%	VUS	chr21	10906983
YA28	MSS	6,68	CANX	MISSENSE	c.676C>G	p.Leu226Val	36%	VUS	chr5	179137021
YA28	MSS	6,68	XPNPEP2	MISSENSE	c.442A>G	p.Ile148Val	49%	VUS	chrX	128880609
YA29	MSS	9,65	APC	TRUNC	c.4099C>T	p.Gln1367Ter	10%	Likely pathogenic	chr5	112175390
YA29	MSS	9,65	TP53	MISSENSE	c.734G>A	p.Gly245Asp	8%	Pathogenic	chr17	7577547
YA29	MSS	9,65	HNF1A	TRUNC	c.511C>T	p.Arg171Ter	5%	Pathogenic	chr12	121426820
YA29	MSS	9,65	TET2	TRUNC	c.3085G>T	p.Glu1029Ter	6%	Likely pathogenic	chr4	106158184
YA29	MSS	9,65	CNTN2	MISSENSE	c.409A>G	p.Lys137Glu	48%	VUS	chr1	205027713
YA29	MSS	9,65	DOCK3	MISSENSE	c.3796G>C	p.Glu1266Gln	5%	VUS	chr3	51378697
YA29	MSS	9,65	FLT4	MISSENSE	c.2111C>T	p.Ala704Val	4%	VUS	chr5	180048162
YA29	MSS	9,65	TAP1	MISSENSE	c.1784C>T	p.Ala595Val	4%	VUS	chr6	32815832
YA29	MSS	9,65	ARID1B	MISSENSE	c.4955C>T	p.Pro1652Leu	42%	VUS	chr6	157522434
YA29	MSS	9,65	TBX3	MISSENSE	c.1126G>A	p.Asp376Asn	3%	VUS	chr12	115112614
YA29	MSS	9,65	ERBB2	MISSENSE	c.1052G>A	p.Arg351Gln	4%	VUS	chr17	37868605
YA29	MSS	9,65	EP300	MISSENSE	c.2512C>T	p.Arg838Cys	4%	VUS	chr22	41545897
YA30	MSS	4,86	KRAS	MISSENSE	c.35G>A	p.Gly12Asp	46%	Pathogenic	chr12	25398284
YA30	MSS	4,86	AKT1	MISSENSE	c.49G>A	p.Glu17Lys	25%	Pathogenic	chr14	105246551
YA30	MSS	4,86	SMAD4	MISSENSE	c.1598T>G	p.Leu533Arg	49%	Pathogenic	chr18	48604776
YA30	MSS	4,86	ELF3	TRUNC	c.764_765de	p.Lys255ArgfsTer45	24%	Likely pathogenic	chr1	201982384
YA30	MSS	4,86	SOX9	TRUNC	c.1137_1138	p.His380ArgfsTer4	22%	Likely pathogenic	chr17	70120133
YA30	MSS	4,86	GPRIN3	MISSENSE	c.1346A>G	p.Glu449Gly	52%	VUS	chr4	90169916
YA30	MSS	4,86	DTX2	MISSENSE	c.352G>A	p.Asp118Asn	50%	VUS	chr7	76111908
YA30	MSS	4,86	CHD4	SPLICE	c.2948+3G>A	p.?	26%	VUS	chr12	6701556
AD1	H-MSI	22,18	RHOA	MISSENSE	c.125A>G	p.Tyr42Cys	14%	Likely pathogenic	chr3	49412898

AD1	H-MSI	22,18	PIK3CA	MISSENSE	c.3140A>G	p.His1047Arg	13%	Pathogenic	chr3	178952085
AD1	H-MSI	22,18	BRAF	MISSENSE	c.1919T>A	p.Val600Glu	14%	Pathogenic	chr7	140453136
AD1	H-MSI	22,18	AXIN1	TRUNC	c.870dup	p.Glu291ArgfsTer60	13%	Likely pathogenic	chr16	396156
AD1	H-MSI	22,18	ORC4	TRUNC	c.492del	p.Phe164LeufsTer24	12%	VUS	chr2	148710038
AD1	H-MSI	22,18	ZFHX3	TRUNC	c.394del	p.Glu132ArgfsTer16	13%	Likely pathogenic	chr16	72993651
AD1	H-MSI	22,18	CDK12	INFRAME	c.548_550de	p.Lys183del	11%	VUS	chr17	37618870
AD1	H-MSI	22,18	FUBP1	MISSENSE	c.803A>G	p.Tyr268Cys	13%	VUS	chr1	78430365
AD1	H-MSI	22,18	DNMT3A	MISSENSE	c.788A>C	p.Glu263Ala	8%	VUS	chr2	25470973
AD1	H-MSI	22,18	DNMT3A	MISSENSE	c.68G>A	p.Arg23Gln	13%	VUS	chr2	25536786
AD1	H-MSI	22,18	BARD1	MISSENSE	c.1109G>A	p.Arg370His	12%	VUS	chr2	215645489
AD1	H-MSI	22,18	ADGRV1	MISSENSE	c.7094T>C	p.Leu2365Pro	11%	VUS	chr5	89988564
AD1	H-MSI	22,18	PRDM1	MISSENSE	c.1196C>T	p.Pro399Leu	18%	VUS	chr6	106553231
AD1	H-MSI	22,18	CSMD3	MISSENSE	c.5491C>A	p.Leu1831Ile	10%	VUS	chr8	113421166
AD1	H-MSI	22,18	CDKN2B	MISSENSE	c.382G>A	p.Val128Ile	12%	VUS	chr9	22006021
AD1	H-MSI	22,18	WT1	MISSENSE	c.122C>T	p.Pro41Leu	57%	VUS	chr11	32456785
AD1	H-MSI	22,18	EMSY	MISSENSE	c.3110A>C	p.Gln1037Pro	9%	VUS	chr11	76255658
AD1	H-MSI	22,18	ANO4	MISSENSE	c.1619C>T	p.Ala540Val	10%	VUS	chr12	101480520
AD1	H-MSI	22,18	CUL4A	MISSENSE	c.2161A>G	p.Asn721Asp	9%	VUS	chr13	113915050
AD1	H-MSI	22,18	CREBBP	MISSENSE	c.2566A>G	p.Thr856Ala	51%	VUS	chr16	3820885
AD1	H-MSI	22,18	MAP2K3	MISSENSE	c.931G>A	p.Ala311Thr	7%	VUS	chr17	21216820
AD1	H-MSI	22,18	BCL2	MISSENSE	c.322T>C	p.Tyr108His	6%	VUS	chr18	60985578
AD1	H-MSI	22,18	ERCC2	MISSENSE	c.658C>A	p.Leu220Met	49%	VUS	chr19	45867742
AD1	H-MSI	22,18	ASXL1	MISSENSE	c.638G>A	p.Gly213Asp	12%	VUS	chr20	31017776
AD1	H-MSI	22,18	E2F1	MISSENSE	c.475G>A	p.Val159Met	55%	VUS	chr20	32267658
AD1	H-MSI	22,18	ZRSR2	MISSENSE	c.980A>G	p.Asn327Ser	12%	VUS	chrX	15840896
AD1	H-MSI	22,18	FAM47C	MISSENSE	c.2726T>C	p.Val909Ala	7%	VUS	chrX	37029209
AD1	H-MSI	22,18	ATRX	MISSENSE	c.1433T>C	p.Val478Ala	8%	VUS	chrX	76939315
AD1	H-MSI	22,18	PRR32	MISSENSE	c.806C>T	p.Pro269Leu	51%	VUS	chrX	125955427
AD1	H-MSI	22,18	AXIN1	SPLICE	c.1116+1G>A		13%	VUS	chr16	359972
AD2	H-MSI	44,14	BRAF	MISSENSE	c.1799T>A	p.Val600Glu	7%	Pathogenic	chr7	140453136
AD2	H-MSI	44,14	APC	TRUNC	c.4393_4394	p.Ser1465TrpfsTer3	9%	Pathogenic	chr5	112175676
AD2	H-MSI	44,14	SMARCB1	TRUNC	c.601C>T	p.Arg201Ter	16%	Pathogenic	chr22	24145582
AD2	H-MSI	44,14	BRCA2	TRUNC	c.5146_5149	p.Tyr1716LysfsTer8	7%	Pathogenic	chr13	32913636

AD2	H-MSI	44,14	SMAD4	TRUNC	c.1249_125(	p.Glu417SerfsTer11	7%	Likely pathogenic	chr18	48593498
AD2	H-MSI	44,14	ARID1B	TRUNC	c.1666C>T	p.Gln556Ter	7%	Likely pathogenic	chr6	157150484
AD2	H-MSI	44,14	MGA	TRUNC	c.7824del	p.Gly2609ValfsTer9	8%	Likely pathogenic	chr15	42057160
AD2	H-MSI	44,14	KMT2D	TRUNC	c.11830C>T	p.Gln3944Ter	13%	Likely pathogenic	chr12	49426658
AD2	H-MSI	44,14	KMT2B	TRUNC	c.2893C>T	p.Arg965Ter	5%	Likely pathogenic	chr19	36214067
AD2	H-MSI	44,14	CASP8	TRUNC	c.463G>T	p.Gly155Ter	8%	Likely pathogenic	chr2	202137412
AD2	H-MSI	44,14	TP53	MISSENSE	c.992A>G	p.Gln331Arg	9%	VUS	chr17	7576854
AD2	H-MSI	44,14	ETV6	TRUNC	c.416_417de	p.Ser139TyrfsTer14	9%	VUS	chr12	12006446
AD2	H-MSI	44,14	ATM	MISSENSE	c.690T>A	p.Asn230Lys	8%	VUS	chr11	108115542
AD2	H-MSI	44,14	ATM	MISSENSE	c.1145T>C	p.Val382Ala	8%	VUS	chr11	108119739
AD2	H-MSI	44,14	POLE	MISSENSE	c.4268T>A	p.Ile1423Asn	7%	VUS	chr12	133220445
AD2	H-MSI	44,14	POLE	MISSENSE	c.1738C>A	p.His580Asn	48%	VUS	chr12	133248857
AD2	H-MSI	44,14	POLE	MISSENSE	c.557C>T	p.Ala186Val	8%	VUS	chr12	133256104
AD2	H-MSI	44,14	ARID2	INFRAME	c.4500_4502	p.Ser1501del	8%	VUS	chr12	46246403
AD2	H-MSI	44,14	MTOR	MISSENSE	c.6979A>G	p.Met2327Val	7%	VUS	chr1	11177098
AD2	H-MSI	44,14	PARP2	SPLICE	c.802+3A>G		6%	VUS	chr14	20822409
AD2	H-MSI	44,14	ABL2	MISSENSE	c.3072G>T	p.Lys1024Asn	9%	VUS	chr1	179077330
AD2	H-MSI	44,14	ERBB4	MISSENSE	c.2150A>G	p.Glu717Gly	6%	VUS	chr2	212488699
AD2	H-MSI	44,14	TGFBR2	MISSENSE	c.1307T>G	p.Leu436Trp	8%	VUS	chr3	30715649
AD2	H-MSI	44,14	MITF	MISSENSE	c.634A>G	p.Met212Val	47%	VUS	chr3	69988300
AD2	H-MSI	44,14	ATR	MISSENSE	c.590T>C	p.Met197Thr	7%	VUS	chr3	142281654
AD2	H-MSI	44,14	BCL6	MISSENSE	c.2033G>A	p.Arg678His	8%	VUS	chr3	187440334
AD2	H-MSI	44,14	NSUN7	MISSENSE	c.1846C>T	p.Pro616Ser	6%	VUS	chr4	40810645
AD2	H-MSI	44,14	FAM71B	MISSENSE	c.1718A>C	p.Lys573Thr	10%	VUS	chr5	156589558
AD2	H-MSI	44,14	PMS2	MISSENSE	c.473G>A	p.Ser158Asn	8%	VUS	chr7	6042148
AD2	H-MSI	44,14	MET	MISSENSE	c.722T>C	p.Phe241Ser	8%	VUS	chr7	116339860
AD2	H-MSI	44,14	WT1	MISSENSE	c.1516C>T	p.Arg506Cys	6%	VUS	chr11	32410657
AD2	H-MSI	44,14	ZFHX3	MISSENSE	c.8954T>C	p.Leu2985Pro	8%	VUS	chr16	72827627
AD2	H-MSI	44,14	RARA	MISSENSE	c.860C>T	p.Ser287Leu	9%	VUS	chr17	38510606
AD2	H-MSI	44,14	PRKAR1A	MISSENSE	c.20C>T	p.Ala7Val	9%	VUS	chr17	66511560
AD2	H-MSI	44,14	MAN2B1	MISSENSE	c.944C>T	p.Thr315Ile	8%	VUS	chr19	12772156
AD2	H-MSI	44,14	MKKS	MISSENSE	c.1532C>T	p.Ser511Phe	8%	VUS	chr20	10386076
AD2	H-MSI	44,14	EP300	MISSENSE	c.2207A>G	p.His736Arg	7%	VUS	chr22	41543916

AD2	H-MSI	44,14	MAP7D2	MISSENSE	c.576A>G	p.Ile192Met	7%	VUS	chrX	20071015
AD2	H-MSI	44,14	BCOR	MISSENSE	c.2629A>G	p.Thr877Ala	9%	VUS	chrX	39931970
AD2	H-MSI	44,14	KLHL13	MISSENSE	c.175T>C	p.Ser59Pro	9%	VUS	chrX	117079471
AD2	H-MSI	44,14	ERBB3	SPLICE	c.875-1G>A		7%	VUS	chr12	56482326
AD2	H-MSI	44,14	NGF	TRUNC	c.18C>A	p.Tyr6Ter	9%	VUS	chr1	115829399
AD3	MSS	6,7	TP53	MISSENSE	c.797G>A	p.Gly266Glu	50%	Pathogenic	chr17	7577141
AD3	MSS	6,7	KRAS	MISSENSE	c.38G>A	p.Gly13Asp	35%	Pathogenic	chr12	25398281
AD3	MSS	6,7	APC	TRUNC	c.2350_2359	p.Ser787IlefsTer4	18%	Pathogenic	chr5	112173640
AD3	MSS	6,7	FGGY	MISSENSE	c.515C>G	p.Pro172Arg	11%	VUS	chr1	59844470
AD3	MSS	6,7	FANCD2	MISSENSE	c.535C>G	p.Gln179Glu	28%	VUS	chr3	10081006
AD3	MSS	6,7	APC	TRUNC	c.1222dup	p.His408ProfsTer11	27%	Pathogenic	chr5	112154951
AD3	MSS	6,7	PDE1C	MISSENSE	c.88C>T	p.Arg30Trp	18%	VUS	chr7	32109918
AD3	MSS	6,7	PON3	MISSENSE	c.275A>G	p.Glu92Gly	45%	VUS	chr7	95001577
AD3	MSS	6,7	PARP4	MISSENSE	c.2789A>G	p.His930Arg	25%	VUS	chr13	25027762
AD3	MSS	6,7	NCOR1	MISSENSE	c.540G>C	p.Glu180Asp	9%	VUS	chr17	16068371
AD3	MSS	6,7	SMARCA4	MISSENSE	c.1706G>A	p.Arg569Gln	51%	VUS	chr19	11107001
AD3	MSS	6,7	POLD1	MISSENSE	c.674G>A	p.Arg225His	55%	VUS	chr19	50905546
AD3	MSS	6,7	KDM5C	MISSENSE	c.3317C>A	p.Ala1106Glu	42%	VUS	chrX	53224234
AD3	MSS	6,7	AMER1	MISSENSE	c.704C>T	p.Pro235Leu	60%	VUS	chrX	63412463
AD4	MSS	6,74	KRAS	MISSENSE	c.38G>A	p.Gly13Asp	30%	Pathogenic	chr12	25398281
AD4	MSS	6,74	APC	TRUNC	c.3100G>T	p.Glu1034Ter	26%	Pathogenic	chr5	112174391
AD4	MSS	6,74	APC	TRUNC	c.4351G>T	p.Glu1451Ter	31%	Pathogenic	chr5	112175642
AD4	MSS	6,74	LATS1	INFRAME	c.2228_2230	p.Leu743del	30%	VUS	chr6	150001374
AD4	MSS	6,74	SPEN	MISSENSE	c.7409C>T	p.Pro2470Leu	25%	VUS	chr1	16260144
AD4	MSS	6,74	PARP3	MISSENSE	c.459C>G	p.His153Gln	57%	VUS	chr3	51978531
AD4	MSS	6,74	SLITRK2	MISSENSE	c.289G>T	p.Gly97Trp	4%	VUS	chrX	144904232
AD4	MSS	6,74	AMER1	TRUNC	c.1891C>T	p.Arg631Ter	61%	Likely pathogenic	chrX	63411276
AD4	MSS	6,74	TP53	SPLICE	c.375+5G>T		44%	VUS	chr17	7579307
AD5	MSS	5,7	BRAF	MISSENSE	c.1798_1799	p.Val600Lys	5%	Pathogenic	chr7	140453136
AD5	MSS	5,7	TP53	MISSENSE	c.536A>G	p.His179Arg	50%	Pathogenic	chr17	7578394
AD5	MSS	5,7	APC	TRUNC	c.3880C>T	p.Gln1294Ter	22%	Pathogenic	chr5	112175171
AD5	MSS	5,7	ZNF862	INFRAME	c.3273_3275	p.Ser1092del	52%	VUS	chr7	149559518
AD5	MSS	5,7	RIT1	MISSENSE	c.367C>A	p.Arg123Ser	42%	VUS	chr1	155874164

AD5	MSS	5,7	PARP3	MISSENSE	c.459C>G	p.His153Gln	42%	VUS	chr3	51978531
AD5	MSS	5,7	FAT1	MISSENSE	c.6256T>C	p.Tyr2086His	62%	VUS	chr4	187541484
AD5	MSS	5,7	POM121L1	MISSENSE	c.241C>T	p.Arg81Trp	39%	VUS	chr7	53103605
AD5	MSS	5,7	TSTD2	MISSENSE	c.311T>G	p.Ile104Ser	40%	VUS	chr9	100388134
AD5	MSS	5,7	DICER1	MISSENSE	c.216T>A	p.Phe72Leu	62%	VUS	chr14	95598943
AD5	MSS	5,7	BCL2L12	MISSENSE	c.211G>C	p.Glu71Gln	48%	VUS	chr19	50169291
AD5	MSS	5,7	KIR3DL1	MISSENSE	c.1238G>A	p.Arg413His	28%	VUS	chr19	55341633
AD5	MSS	5,7	C8B	TRUNC	c.361C>T	p.Arg121Ter	26%	VUS	chr1	57422472
AD6	H-MSI	19	TP53	MISSENSE	c.817C>T	p.Arg273Cys	17%	Pathogenic	chr17	7577121
AD6	H-MSI	19	CDC73	TRUNC	c.687_688du	p.Val230GlufsTer28	33%	Pathogenic	chr1	193111146
AD6	H-MSI	19	KMT2D	MISSENSE	c.15641G>A	p.Arg5214His	31%	Pathogenic	chr12	49420108
AD6	H-MSI	19	PHF6	TRUNC	c.820C>T	p.Arg274Ter	5%	Pathogenic	chrX	133549136
AD6	H-MSI	19	TP53	MISSENSE	c.623A>G	p.Asp208Gly	7%	VUS	chr17	7578226
AD6	H-MSI	19	ERAP1	TRUNC	c.1967del	p.Lys656ArgfsTer9	35%	VUS	chr5	96119761
AD6	H-MSI	19	KMT2D	TRUNC	c.7061del	p.Pro2354LeufsTer30	21%	Likely pathogenic	chr12	49434492
AD6	H-MSI	19	CREBBP	TRUNC	c.1050del	p.Lys350AsnfsTer4	38%	Likely pathogenic	chr16	3843553
AD6	H-MSI	19	TET2	TRUNC	c.2884_2885	p.Pro963LeufsTer12	6%	Likely pathogenic	chr4	106157918
AD6	H-MSI	19	LMX1A	MISSENSE	c.328G>A	p.Ala110Thr	36%	VUS	chr1	165218813
AD6	H-MSI	19	ALK	MISSENSE	c.3546C>G	p.Cys1182Trp	3%	VUS	chr2	29443671
AD6	H-MSI	19	LRP1B	MISSENSE	c.100C>A	p.Pro34Thr	36%	VUS	chr2	142567953
AD6	H-MSI	19	PBRM1	MISSENSE	c.2869A>G	p.Met957Val	12%	VUS	chr3	52623182
AD6	H-MSI	19	MAP3K1	MISSENSE	c.1510G>A	p.Glu504Lys	11%	VUS	chr5	56168656
AD6	H-MSI	19	ERAP2	MISSENSE	c.2215T>G	p.Trp739Gly	20%	VUS	chr5	96245329
AD6	H-MSI	19	LATS1	MISSENSE	c.994A>G	p.Met332Val	23%	VUS	chr6	150005231
AD6	H-MSI	19	PDE1C	MISSENSE	c.364G>A	p.Asp122Asn	39%	VUS	chr7	31918670
AD6	H-MSI	19	SAMD9	MISSENSE	c.1420A>C	p.Thr474Pro	13%	VUS	chr7	92733991
AD6	H-MSI	19	IKBKB	MISSENSE	c.397C>T	p.Leu133Phe	5%	VUS	chr8	42162713
AD6	H-MSI	19	PTCH1	MISSENSE	c.3080G>T	p.Trp1027Leu	38%	VUS	chr9	98220383
AD6	H-MSI	19	NOTCH1	MISSENSE	c.3787C>T	p.Arg1263Cys	9%	VUS	chr9	139401282
AD6	H-MSI	19	FGF3	MISSENSE	c.188G>A	p.Arg63His	42%	VUS	chr11	69633514
AD6	H-MSI	19	FGF23	MISSENSE	c.265A>C	p.Met89Leu	6%	VUS	chr12	4481810
AD6	H-MSI	19	KLRC4	MISSENSE	c.105A>C	p.Lys35Asn	39%	VUS	chr12	10562070
AD6	H-MSI	19	KMT2D	MISSENSE	c.14236C>T	p.Arg4746Trp	38%	VUS	chr12	49422859

AD6	H-MSI	19	KMT2D	MISSENSE	c.5174G>T	p.Gly1725Val	30%	VUS	chr12	49437997
AD6	H-MSI	19	KMT2D	MISSENSE	c.1957C>A	p.Leu653Ile	42%	VUS	chr12	49445509
AD6	H-MSI	19	CD276	MISSENSE	c.313G>A	p.Ala105Thr	26%	VUS	chr15	73994829
AD6	H-MSI	19	CDH1	MISSENSE	c.466T>C	p.Trp156Arg	9%	VUS	chr16	68842405
AD6	H-MSI	19	KMT2B	MISSENSE	c.6286C>A	p.Leu2096Ile	10%	VUS	chr19	36223736
AD6	H-MSI	19	EP300	MISSENSE	c.6028A>G	p.Met2010Val	11%	VUS	chr22	41573743
AD6	H-MSI	19	CUL4B	MISSENSE	c.2269G>T	p.Asp757Tyr	30%	VUS	chrX	119666447
AD6	H-MSI	19	GATA3	TRUNC	c.778G>T	p.Glu260Ter	23%	Likely pathogenic	chr10	8100804
AD6	H-MSI	19	STAG2	TRUNC	c.1717C>T	p.Gln573Ter	28%	Likely pathogenic	chrX	123196830
AD7	MSS	12,35	KRAS	MISSENSE	c.13A>G	p.Lys5Glu	21%	Likely pathogenic	chr12	25398306
AD7	MSS	12,35	TP53	MISSENSE	c.524G>A	p.Arg175His	36%	Pathogenic	chr17	7578406
AD7	MSS	12,35	STK11	SPLICE	c.920+1G>C		31%	Likely pathogenic	chr19	1222006
AD7	MSS	12,35	APC	TRUNC	c.2626C>T	p.Arg876Ter	17%	Pathogenic	chr5	112173917
AD7	MSS	12,35	APC	TRUNC	c.3892del	p.Ser1298LeufsTer7	22%	Likely pathogenic	chr5	112175182
AD7	MSS	12,35	EPHA2	MISSENSE	c.2446C>T	p.Arg816Trp	37%	VUS	chr1	16458245
AD7	MSS	12,35	DPYD	MISSENSE	c.2801C>T	p.Thr934Ile	45%	VUS	chr1	97547992
AD7	MSS	12,35	CACNA1D	MISSENSE	c.1382A>G	p.Lys461Arg	36%	VUS	chr3	53736829
AD7	MSS	12,35	PPP2R3A	MISSENSE	c.523G>T	p.Gly175Cys	52%	VUS	chr3	135720863
AD7	MSS	12,35	ATM	MISSENSE	c.8063C>T	p.Ala2688Val	25%	VUS	chr11	108205748
AD7	MSS	12,35	LATS2	MISSENSE	c.1129C>T	p.Arg377Cys	27%	VUS	chr13	21562790
AD7	MSS	12,35	SOX9	MISSENSE	c.352G>A	p.Ala118Thr	37%	VUS	chr17	70117884
AD7	MSS	12,35	SMCHD1	MISSENSE	c.4599G>T	p.Leu1533Phe	30%	VUS	chr18	2763667
AD7	MSS	12,35	PAK5	MISSENSE	c.2065G>A	p.Ala689Thr	28%	VUS	chr20	9520204
AD8	MSS	5,68	APC	TRUNC	c.4666dup	p.Thr1556AsnfsTer3	7%	Pathogenic	chr5	112175952
AD8	MSS	5,68	KRAS	MISSENSE	c.38G>A	p.Gly13Asp	15%	Pathogenic	chr12	25398281
AD8	MSS	5,68	TP53	MISSENSE	c.733G>A	p.Gly245Ser	17%	Pathogenic	chr17	7577548
AD8	MSS	5,68	PIK3CA	MISSENSE	c.1048G>A	p.Asp350Asn	12%	Likely pathogenic	chr3	178921566
AD8	MSS	5,68	EPS15	MISSENSE	c.2437G>A	p.Asp813Asn	51%	VUS	chr1	51826950
AD8	MSS	5,68	CASP8	MISSENSE	c.11G>T	p.Gly4Val	18%	VUS	chr2	202122965
AD8	MSS	5,68	PIK3CA	MISSENSE	c.1632_1633	p.Glu545Lys	16%	Pathogenic	chr3	178936090
AD8	MSS	5,68	RICTOR	MISSENSE	c.1904A>G	p.Asn635Ser	44%	VUS	chr5	38960028
AD8	MSS	5,68	ADAMTS2	MISSENSE	c.1000A>T	p.Asn334Tyr	50%	VUS	chr5	178585856
AD8	MSS	5,68	SMAD4	MISSENSE	c.578A>G	p.Glu193Gly	3%	VUS	chr18	48581274

AD8	MSS	5,68	HDAC9	MISSENSE	c.2023G>T	p.Gly675Trp	3%	VUS	chr7	18788741
AD9	MSS	9,5	KRAS	MISSENSE	c.35G>T	p.Gly12Val	11%	Pathogenic	chr12	25398284
AD9	MSS	9,5	SMAD4	TRUNC	c.1304_1305	p.Lys436Ter	12%	Pathogenic	chr18	48593555
AD9	MSS	9,5	APC	TRUNC	c.3880_3881	p.Gln1294GlyfsTer6	8%	Likely pathogenic	chr5	112175168
AD9	MSS	9,5	DOCK3	MISSENSE	c.3132A>G	p.Ile1044Met	51%	VUS	chr3	51349945
AD9	MSS	9,5	PBRM1	MISSENSE	c.1504T>G	p.Ser502Ala	48%	VUS	chr3	52661326
AD9	MSS	9,5	APC	TRUNC	c.3340C>T	p.Arg1114Ter	8%	Pathogenic	chr5	112174631
AD9	MSS	9,5	CREBBP	MISSENSE	c.5705C>T	p.Thr1902Met	8%	VUS	chr16	3779343
AD9	MSS	9,5	TP53	MISSENSE	c.482C>A	p.Ala161Asp	13%	VUS	chr17	7578448
AD9	MSS	9,5	CHEK2	MISSENSE	c.1556G>T	p.Arg519Leu	36%	VUS	chr22	29083961
AD9	MSS	9,5	DPYD	MISSENSE	c.2846A>T	p.Asp949Val	50%	VUS	chr1	97547947
AD10	H-MSI	49,3	MSH6	MISSENSE	c.2314C>T	p.Arg772Trp	49%	Pathogenic	chr2	48027436
AD10	H-MSI	49,3	MUTYH	MISSENSE	c.241C>T	p.Arg81Trp	29%	Pathogenic	chr1	45799108
AD10	H-MSI	49,3	TNFRSF14	MISSENSE	c.410C>T	p.Ala137Val	33%	VUS	chr1	2491367
AD10	H-MSI	49,3	MTOR	MISSENSE	c.4444C>T	p.Arg1482Cys	29%	Likely pathogenic	chr1	11217234
AD10	H-MSI	49,3	SPEN	MISSENSE	c.10747C>T	p.Arg3583Cys	29%	VUS	chr1	16265255
AD10	H-MSI	49,3	EPHA2	MISSENSE	c.307C>T	p.Arg103Cys	3%	VUS	chr1	16475389
AD10	H-MSI	49,3	ARID1A	MISSENSE	c.3815C>T	p.Ala1272Val	32%	VUS	chr1	27099936
AD10	H-MSI	49,3	RAD54L	MISSENSE	c.1375C>T	p.His459Tyr	32%	VUS	chr1	46738474
AD10	H-MSI	49,3	DPYD	MISSENSE	c.236G>A	p.Cys79Tyr	27%	VUS	chr1	98206033
AD10	H-MSI	49,3	COL11A1	MISSENSE	c.4964C>A	p.Ser1655Tyr	29%	VUS	chr1	103348762
AD10	H-MSI	49,3	LMX1A	MISSENSE	c.367T>G	p.Cys123Gly	49%	VUS	chr1	165218774
AD10	H-MSI	49,3	MYCN	MISSENSE	c.1136A>G	p.Asp379Gly	30%	VUS	chr2	16085960
AD10	H-MSI	49,3	ASXL2	MISSENSE	c.2900T>A	p.Ile967Asn	3%	VUS	chr2	25966306
AD10	H-MSI	49,3	MSH6	MISSENSE	c.3G>A	p.Met1?	10%	VUS	chr2	48010375
AD10	H-MSI	49,3	MSH6	TRUNC	c.960del	p.Ser321GlnfsTer17	30%	Likely pathogenic	chr2	48026080
AD10	H-MSI	49,3	MSH6	MISSENSE	c.962C>T	p.Ser321Leu	30%	VUS	chr2	48026084
AD10	H-MSI	49,3	REV1	MISSENSE	c.443G>A	p.Cys148Tyr	25%	VUS	chr2	100058839
AD10	H-MSI	49,3	TGFBR2	MISSENSE	c.1583G>A	p.Arg528His	58%	Pathogenic	chr3	30732970
AD10	H-MSI	49,3	PBRM1	TRUNC	c.2776del	p.Arg926GlufsTer88	62%	Likely pathogenic	chr3	52637545
AD10	H-MSI	49,3	MECOM	MISSENSE	c.3000T>G	p.His1000Gln	22%	VUS	chr3	168812883
AD10	H-MSI	49,3	PIK3CA	MISSENSE	c.332A>G	p.Lys111Arg	22%	Likely pathogenic	chr3	178916945
AD10	H-MSI	49,3	FGFR3	TRUNC	c.1311_1312	p.Pro438ThrfsTer59	4%	Likely pathogenic	chr4	1806585

AD10	H-MSI	49,3	TET2	MISSENSE	c.1694G>A	p.Arg565Gln	29%	VUS	chr4	106156730
AD10	H-MSI	49,3	TET2	MISSENSE	c.3829G>A	p.Gly1277Ser	30%	VUS	chr4	106164898
AD10	H-MSI	49,3	FAT1	MISSENSE	c.12788A>G	p.Asp4263Gly	29%	VUS	chr4	187517906
AD10	H-MSI	49,3	HCN1	SPLICE	c.1619-1G>T		29%	VUS	chr5	45267356
AD10	H-MSI	49,3	MSH3	MISSENSE	c.1241T>C	p.Leu414Pro	30%	VUS	chr5	79974813
AD10	H-MSI	49,3	APC	TRUNC	c.646C>T	p.Arg216Ter	29%	Pathogenic	chr5	112128143
AD10	H-MSI	49,3	APC	TRUNC	c.4012C>T	p.Gln1338Ter	30%	Pathogenic	chr5	112175303
AD10	H-MSI	49,3	APC	MISSENSE	c.5362C>T	p.Arg1788Cys	29%	VUS	chr5	112176653
AD10	H-MSI	49,3	PSMB8	MISSENSE	c.385C>T	p.Arg129Cys	58%	VUS	chr6	32810471
AD10	H-MSI	49,3	HTR1B	MISSENSE	c.103G>A	p.Ala35Thr	27%	VUS	chr6	78173018
AD10	H-MSI	49,3	IBTK	MISSENSE	c.1001C>T	p.Ala334Val	4%	VUS	chr6	82933280
AD10	H-MSI	49,3	PRDM1	MISSENSE	c.2233G>A	p.Val745Met	3%	VUS	chr6	106555116
AD10	H-MSI	49,3	GLI3	MISSENSE	c.2905G>A	p.Ala969Thr	29%	VUS	chr7	42005766
AD10	H-MSI	49,3	KEL	MISSENSE	c.1085A>G	p.Gln362Arg	17%	VUS	chr7	142649714
AD10	H-MSI	49,3	KMT2C	MISSENSE	c.11875G>A	p.Ala3959Thr	16%	VUS	chr7	151853080
AD10	H-MSI	49,3	KMT2C	TRUNC	c.11455G>T	p.Gly3819Ter	54%	Likely pathogenic	chr7	151859207
AD10	H-MSI	49,3	KMT2C	MISSENSE	c.10807A>G	p.Lys3603Glu	15%	VUS	chr7	151859855
AD10	H-MSI	49,3	KMT2C	MISSENSE	c.10547T>A	p.Val3516Asp	31%	VUS	chr7	151860115
AD10	H-MSI	49,3	PKHD1L1	MISSENSE	c.1398G>T	p.Gln466His	25%	VUS	chr8	110416807
AD10	H-MSI	49,3	PRUNE2	MISSENSE	c.5147C>G	p.Ala1716Gly	60%	VUS	chr9	79322043
AD10	H-MSI	49,3	PTCH1	MISSENSE	c.2794G>A	p.Val932Ile	27%	VUS	chr9	98221975
AD10	H-MSI	49,3	LARP4B	MISSENSE	c.44C>T	p.Thr15Met	51%	VUS	chr10	931618
AD10	H-MSI	49,3	SUFU	MISSENSE	c.816G>T	p.Lys272Asn	44%	VUS	chr10	104356956
AD10	H-MSI	49,3	TCF7L2	MISSENSE	c.1751G>A	p.Ser584Asn	16%	VUS	chr10	114925673
AD10	H-MSI	49,3	MEN1	MISSENSE	c.1166G>A	p.Arg389Gln	10%	VUS	chr11	64573126
AD10	H-MSI	49,3	ATM	MISSENSE	c.7357C>T	p.Arg2453Cys	27%	VUS	chr11	108200990
AD10	H-MSI	49,3	KMT2A	MISSENSE	c.10418G>A	p.Arg3473His	37%	VUS	chr11	118377025
AD10	H-MSI	49,3	RAD52	MISSENSE	c.1211A>G	p.His404Arg	47%	VUS	chr12	1022603
AD10	H-MSI	49,3	RAD52	MISSENSE	c.572C>T	p.Ala191Val	25%	VUS	chr12	1025958
AD10	H-MSI	49,3	RAD52	MISSENSE	c.262A>G	p.Ile88Val	22%	VUS	chr12	1039235
AD10	H-MSI	49,3	KRAS	MISSENSE	c.35G>A	p.Gly12Asp	41%	Pathogenic	chr12	25398284
AD10	H-MSI	49,3	ARID2	MISSENSE	c.376G>A	p.Ala126Thr	39%	VUS	chr12	46205292
AD10	H-MSI	49,3	LATS2	SPLICE	c.2666-1G>T		23%	VUS	chr13	21553937

AD10	H-MSI	49,3	LATS2	MISSENSE	c.1738G>A	p.Val580Ile	19%	VUS	chr13	21562181
AD10	H-MSI	49,3	PARP4	MISSENSE	c.770G>A	p.Ser257Asn	44%	VUS	chr13	25067843
AD10	H-MSI	49,3	RB1	TRUNC	c.2622dup	p.Leu875ThrfsTer4	9%	Likely pathogenic	chr13	49050934
AD10	H-MSI	49,3	PCNX1	MISSENSE	c.1702C>T	p.Arg568Trp	32%	VUS	chr14	71444756
AD10	H-MSI	49,3	MLH3	MISSENSE	c.194A>G	p.Asp65Gly	30%	VUS	chr14	75516165
AD10	H-MSI	49,3	PDIA3	MISSENSE	c.1046T>C	p.Leu349Pro	30%	VUS	chr15	44060704
AD10	H-MSI	49,3	FANCI	MISSENSE	c.3824T>C	p.Leu1275Pro	28%	VUS	chr15	89858520
AD10	H-MSI	49,3	CREBBP	MISSENSE	c.6793G>A	p.Ala2265Thr	27%	VUS	chr16	3778255
AD10	H-MSI	49,3	CREBBP	MISSENSE	c.6789G>T	p.Gln2263His	26%	VUS	chr16	3778259
AD10	H-MSI	49,3	CREBBP	MISSENSE	c.6227C>T	p.Ser2076Leu	25%	VUS	chr16	3778821
AD10	H-MSI	49,3	CREBBP	MISSENSE	c.5879G>A	p.Arg1960Gln	32%	VUS	chr16	3779169
AD10	H-MSI	49,3	CREBBP	MISSENSE	c.4807G>A	p.Ala1603Thr	15%	VUS	chr16	3781860
AD10	H-MSI	49,3	CREBBP	SPLICE	c.3836+1G>A		14%	Pathogenic	chr16	3799627
AD10	H-MSI	49,3	CDK12	MISSENSE	c.1811C>A	p.Pro604His	31%	VUS	chr17	37627896
AD10	H-MSI	49,3	RARA	SPLICE	c.1172-1G>T		29%	VUS	chr17	38512260
AD10	H-MSI	49,3	RNF43	TRUNC	c.1976del	p.Gly659ValfsTer41	3%	Pathogenic	chr17	56435161
AD10	H-MSI	49,3	BRIP1	MISSENSE	c.2963C>T	p.Ser988Phe	30%	VUS	chr17	59761444
AD10	H-MSI	49,3	AXIN2	MISSENSE	c.950G>A	p.Ser317Asn	27%	VUS	chr17	63545644
AD10	H-MSI	49,3	SOX9	TRUNC	c.490C>T	p.Gln164Ter	26%	Likely pathogenic	chr17	70118918
AD10	H-MSI	49,3	SOX9	MISSENSE	c.491A>C	p.Gln164Pro	27%	Pathogenic	chr17	70118919
AD10	H-MSI	49,3	SETBP1	MISSENSE	c.842A>G	p.Asn281Ser	29%	VUS	chr18	42530147
AD10	H-MSI	49,3	DYNAP	MISSENSE	c.617C>A	p.Pro206His	28%	VUS	chr18	52265360
AD10	H-MSI	49,3	NOTCH3	MISSENSE	c.901G>A	p.Val301Met	25%	VUS	chr19	15302370
AD10	H-MSI	49,3	CCNE1	MISSENSE	c.1198G>A	p.Gly400Ser	35%	VUS	chr19	30314649
AD10	H-MSI	49,3	KMT2B	MISSENSE	c.1828C>T	p.Arg610Cys	26%	VUS	chr19	36212077
AD10	H-MSI	49,3	KMT2B	MISSENSE	c.3577C>T	p.Leu1193Phe	31%	VUS	chr19	36216169
AD10	H-MSI	49,3	CIC	MISSENSE	c.2150A>C	p.Glu717Ala	36%	VUS	chr19	42778085
AD10	H-MSI	49,3	POLD1	MISSENSE	c.671G>A	p.Arg224His	26%	VUS	chr19	50905543
AD10	H-MSI	49,3	PTPRT	MISSENSE	c.1830G>A	p.Met610Ile	21%	VUS	chr20	40979303
AD10	H-MSI	49,3	ATP6AP2	MISSENSE	c.829A>G	p.Thr277Ala	66%	VUS	chrX	40460104
AD10	H-MSI	49,3	STAG2	MISSENSE	c.2858G>A	p.Arg953Gln	78%	VUS	chrX	123215312
AD11	MSS	3,78	TP53	MISSENSE	c.524G>A	p.Arg175His	53%	Pathogenic	chr17	7578406
AD11	MSS	3,78	SMAD4	MISSENSE	c.1082G>A	p.Arg361His	34%	Pathogenic	chr18	48591919

AD11	MSS	3,78	SETD2	MISSENSE	c.2546G>T	p.Cys849Phe	58%	VUS	chr3	47163580
AD11	MSS	3,78	ATR	MISSENSE	c.1945C>G	p.Leu649Val	61%	VUS	chr3	142275358
AD11	MSS	3,78	RASA1	MISSENSE	c.3122C>T	p.Thr1041Ile	62%	VUS	chr5	86686678
AD11	MSS	3,78	XRCC2	MISSENSE	c.625C>A	p.Pro209Thr	53%	VUS	chr7	152345945
AD11	MSS	3,78	KMT2D	MISSENSE	c.983C>T	p.Ala328Val	20%	VUS	chr12	49446827
AD11	MSS	3,78	KMT2D	MISSENSE	c.341A>G	p.Asp114Gly	60%	VUS	chr12	49448370
AD11	MSS	3,78	MTUS2	MISSENSE	c.2540G>A	p.Arg847His	15%	VUS	chr13	29674973
AD11	MSS	3,78	RAD51B	MISSENSE	c.535C>T	p.Leu179Phe	54%	VUS	chr14	68352668
AD11	MSS	3,78	FANCI	MISSENSE	c.350G>C	p.Arg117Thr	49%	VUS	chr15	89804877
AD11	MSS	3,78	RAD51D	MISSENSE	c.932G>A	p.Arg311His	77%	VUS	chr17	33428251
AD11	MSS	3,78	POLD1	MISSENSE	c.1505A>G	p.Asp502Gly	56%	VUS	chr19	50910250
AD12	MSS	3,79	BRAF	MISSENSE	c.1799T>A	p.Val600Glu	20%	Pathogenic	chr7	140453136
AD12	MSS	3,79	TP53	TRUNC	c.637C>T	p.Arg213Ter	22%	Pathogenic	chr17	7578212
AD12	MSS	3,79	POT1	TRUNC	c.1306_1307	p.Val436AspfsTer7	21%	Likely pathogenic	chr7	124481090
AD12	MSS	3,79	KEL	TRUNC	c.1912dup	p.Asp638GlyfsTer46	12%	VUS	chr7	142639991
AD12	MSS	3,79	CHEK1	TRUNC	c.737_738in	p.Trp247LeufsTer14	23%	Likely pathogenic	chr11	125505400
AD12	MSS	3,79	CASP8	MISSENSE	c.91G>T	p.Val31Leu	30%	VUS	chr2	202123045
AD12	MSS	3,79	SETD2	MISSENSE	c.2054C>A	p.Thr685Lys	25%	VUS	chr3	47164072
AD12	MSS	3,79	EYA1	MISSENSE	c.1702G>A	p.Ala568Thr	42%	VUS	chr8	72111652
AD12	MSS	3,79	NOTCH1	MISSENSE	c.5608G>A	p.Asp1870Asn	56%	VUS	chr9	139396230
AD13	L-MSI	11,34	APC	TRUNC	c.4033G>T	p.Glu1345Ter	24%	Pathogenic	chr5	112175324
AD13	L-MSI	11,34	KRAS	MISSENSE	c.34G>A	p.Gly12Ser	28%	Pathogenic	chr12	25398285
AD13	L-MSI	11,34	APC	TRUNC	c.1837del	p.Ala614HisfsTer16	30%	Likely pathogenic	chr5	112170741
AD13	L-MSI	11,34	NOTCH4	MISSENSE	c.4825T>C	p.Trp1609Arg	43%	VUS	chr6	32165303
AD13	L-MSI	11,34	LARGE2	MISSENSE	c.403G>A	p.Ala135Thr	51%	VUS	chr11	45945739
AD13	L-MSI	11,34	ATM	MISSENSE	c.2579A>T	p.Asp860Val	53%	VUS	chr11	108138010
AD13	L-MSI	11,34	FLT3	MISSENSE	c.2418+1G>(	intronic variant	21%	VUS	chr13	28597486
AD13	L-MSI	11,34	SLX4	MISSENSE	c.299C>A	p.Thr100Asn	31%	VUS	chr16	3658667
AD13	L-MSI	11,34	MAP2K4	MISSENSE	c.551C>T	p.Ser184Leu	33%	VUS	chr17	12011144
AD13	L-MSI	11,34	MBOAT2	MISSENSE	c.1462G>A	p.Glu488Lys	46%	VUS	chr2	8998910
AD13	L-MSI	11,34	SETD2	MISSENSE	c.6241C>T	p.Leu2081Phe	49%	VUS	chr3	47103705
AD13	L-MSI	11,34	DOCK3	MISSENSE	c.1430T>G	p.Phe477Cys	8%	VUS	chr3	51264766
AD13	L-MSI	11,34	FAT1	SPLICE	c.10206+2_1	intronic variant	29%	VUS	chr4	187530334

AD14	MSS	7,59	APC	TRUNC	c.2547_2548	p.Asp849GlufsTer62	8%	Pathogenic	chr5	112173837
AD14	MSS	7,59	APC	TRUNC	c.4348C>T	p.Arg1450Ter	6%	Pathogenic	chr5	112175639
AD14	MSS	7,59	TP53	MISSENSE	c.733G>C	p.Gly245Arg	14%	Likely pathogenic	chr17	7577548
AD14	MSS	7,59	ARID2	TRUNC	c.1834_1843	p.Tyr612AsnfsTer32	12%	Likely pathogenic	chr12	46243480
AD14	MSS	7,59	PIK3CA	MISSENSE	c.1618C>T	p.Leu540Phe	8%	VUS	chr3	178936076
AD14	MSS	7,59	TET2	MISSENSE	c.5636A>T	p.Glu1879Val	4%	VUS	chr4	106197303
AD14	MSS	7,59	ARID2	MISSENSE	c.83A>G	p.His28Arg	9%	VUS	chr12	46123702
AD14	MSS	7,59	CD276	MISSENSE	c.664G>A	p.Val222Met	51%	VUS	chr15	73995358
AD14	MSS	7,59	NF2	MISSENSE	c.871C>T	p.Arg291Cys	44%	VUS	chr22	30061039
AD15	MSS	8,52	APC	TRUNC	c.4348C>T	p.Arg1450Ter	62%	Pathogenic	chr5	112175639
AD15	MSS	8,52	KRAS	MISSENSE	c.35G>T	p.Gly12Val	30%	Pathogenic	chr12	25398284
AD15	MSS	8,52	TP53	TRUNC	c.224_233de	p.Pro75GlnfsTer45	49%	Likely pathogenic	chr17	7579455
AD15	MSS	8,52	ACVR2A	TRUNC	c.1403_1407	p.Ala468ValfsTer8	43%	Likely pathogenic	chr2	148684704
AD15	MSS	8,52	ARID2	TRUNC	c.159_160du	p.Val54GlufsTer5	31%	Likely pathogenic	chr12	46123891
AD15	MSS	8,52	ARID2	TRUNC	c.2720C>A	p.Ser907Ter	28%	Likely pathogenic	chr12	46244626
AD15	MSS	8,52	SOX9	TRUNC	c.1315C>T	p.Gln439Ter	33%	Likely pathogenic	chr17	70120313
AD15	MSS	8,52	CTNNB1	MISSENSE	c.1147T>A	p.Trp383Arg	44%	VUS	chr3	41274897
AD15	MSS	8,52	HDAC9	MISSENSE	c.740G>T	p.Arg247Met	7%	VUS	chr7	18669048
AD15	MSS	8,52	KMT2C	MISSENSE	c.10192C>T	p.Arg3398Trp	49%	VUS	chr7	151860470
AD15	MSS	8,52	TCF7L2	MISSENSE	c.1385C>T	p.Pro462Leu	61%	VUS	chr10	114920444
AD15	MSS	8,52	NTF3	MISSENSE	c.261_266de	p.Gly89Glu	54%	VUS	chr12	5603602
AD15	MSS	8,52	LATS2	MISSENSE	c.2713G>A	p.Glu905Lys	3%	VUS	chr13	21553889
AD15	MSS	8,52	AGBL1	MISSENSE	c.977A>C	p.Asp326Ala	30%	VUS	chr15	86806016
AD15	MSS	8,52	CNTNAP4	MISSENSE	c.2060C>T	p.Pro687Leu	33%	VUS	chr16	76532505
AD15	MSS	8,52	NF1	INFRAME	c.3179_3181	p.Asp1060del	56%	VUS	chr17	29557925
AD16	MSS	5,69	KRAS	MISSENSE	c.35G>T	p.Gly12Val	19%	Pathogenic	chr12	25398284
AD16	MSS	5,69	PIK3CA	MISSENSE	c.3140A>G	p.His1047Arg	12%	Pathogenic	chr3	178952085
AD16	MSS	5,69	APC	TRUNC	c.3927_3931	p.Glu1309AspfsTer4	12%	Pathogenic	chr5	112175212
AD16	MSS	5,69	SOX9	TRUNC	c.542C>A	p.Ser181Ter	18%	Likely pathogenic	chr17	70118970
AD16	MSS	5,69	APC	TRUNC	c.1875_1878	p.Asn627LeufsTer2	11%	Likely pathogenic	chr5	112170779
AD16	MSS	5,69	DPYD	TRUNC	c.2703del	p.Asn901LysfsTer2	47%	VUS	chr1	97564108
AD16	MSS	5,69	MYC	TRUNC	c.30_31insG	p.Gln11ValfsTer108	24%	VUS	chr8	128748869
AD16	MSS	5,69	PCBP1	MISSENSE	c.299T>A	p.Leu100Gln	24%	VUS	chr2	70315174

AD16	MSS	5,69	SETD2	MISSENSE	c.4307C>T	p.Thr1436Ile	48%	VUS	chr3	47161819
AD16	MSS	5,69	KMT2C	MISSENSE	c.1331G>A	p.Arg444Gln	41%	VUS	chr7	151949769
AD16	MSS	5,69	ZRSR2	MISSENSE	c.782A>G	p.Asn261Ser	16%	VUS	chrX	15836720
AD16	MSS	5,69	FANCM	MISSENSE	c.322C>A	p.Leu108Met	4%	VUS	chr14	45605556
AD17	MSS	4,75	BRAF	MISSENSE	c.1799T>A	p.Val600Glu	25%	Pathogenic	chr7	140453136
AD17	MSS	4,75	PIK3CA	MISSENSE	c.1633G>A	p.Glu545Lys	35%	Pathogenic	chr3	178936091
AD17	MSS	4,75	TP53	TRUNC	c.916C>T	p.Arg306Ter	33%	Pathogenic	chr17	7577022
AD17	MSS	4,75	SMAD4	MISSENSE	c.1082G>A	p.Arg361His	18%	Pathogenic	chr18	48591919
AD17	MSS	4,75	MSH6	MISSENSE	c.2926C>T	p.Arg976Cys	5%	Likely pathogenic	chr2	48028048
AD17	MSS	4,75	PDCD6IP	MISSENSE	c.946G>A	p.Asp316Asn	61%	VUS	chr3	33877647
AD17	MSS	4,75	ACSL1	MISSENSE	c.1240C>T	p.Arg414Trp	22%	VUS	chr4	185687799
AD17	MSS	4,75	TG	MISSENSE	c.7648G>A	p.Glu2550Lys	47%	VUS	chr8	134125741
AD17	MSS	4,75	CDKN2A	MISSENSE	c.334C>T	p.Arg112Cys	8%	VUS	chr9	21971024
AD17	MSS	4,75	CDKN2B	MISSENSE	c.407C>T	p.Thr136Met	43%	VUS	chr9	22005996
AD17	MSS	4,75	TCF7L2	MISSENSE	c.748G>A	p.Val250Ile	47%	VUS	chr10	114903744
AD17	MSS	4,75	AXIN1	MISSENSE	c.2243C>T	p.Pro748Leu	28%	VUS	chr16	341241
AD17	MSS	4,75	KMT2B	MISSENSE	c.328G>A	p.Glu110Lys	4%	VUS	chr19	36209248
AD17	MSS	4,75	POLD1	MISSENSE	c.923C>T	p.Ala308Val	31%	VUS	chr19	50905951
AD18	MSS	5,68	ACVR1	MISSENSE	c.617G>A	p.Arg206His	37%	Pathogenic	chr2	158630626
AD18	MSS	5,68	APC	SPLICE	c.835-8A>G	Intronic variant	48%	Pathogenic	chr5	112151184
AD18	MSS	5,68	SETD2	MISSENSE	c.4034A>G	p.Asp1345Gly	37%	VUS	chr3	47162092
AD18	MSS	5,68	PRDM9	MISSENSE	c.652G>T	p.Ala218Ser	55%	VUS	chr5	23522764
AD18	MSS	5,68	PDE1C	MISSENSE	c.1406G>T	p.Ser469Ile	57%	VUS	chr7	31864481
AD18	MSS	5,68	CD274	MISSENSE	c.723G>C	p.Leu241Phe	79%	VUS	chr9	5465539
AD18	MSS	5,68	ABL1	MISSENSE	c.760G>A	p.Gly254Arg	52%	VUS	chr9	133738360
AD18	MSS	5,68	TBX3	MISSENSE	c.927C>G	p.His309Gln	36%	VUS	chr12	115114230
AD18	MSS	5,68	SRRM4	MISSENSE	c.1172G>A	p.Arg391His	54%	VUS	chr12	119588917
AD18	MSS	5,68	MAP2K7	MISSENSE	c.980C>T	p.Thr327Met	28%	VUS	chr19	7976364
AD18	MSS	5,68	AMER1	MISSENSE	c.1973C>T	p.Pro658Leu	70%	VUS	chrX	63411194
AD19	MSS	8,95	KRAS	MISSENSE	c.175G>A	p.Ala59Thr	28%	Pathogenic	chr12	25380283
AD19	MSS	8,95	TP53	MISSENSE	c.524G>A	p.Arg175His	45%	Pathogenic	chr17	7578406
AD19	MSS	8,95	FBXW7	MISSENSE	c.1394G>A	p.Arg465His	43%	Likely pathogenic	chr4	153249384
AD19	MSS	8,95	TCF7L2	TRUNC	c.1704_170[illegible]	p.Ser569LeufsTer40	10%	Likely pathogenic	chr10	114925621

AD19	MSS	8,95	ACVR2A	INFRAME	c.1296_1298	p.Val433del	26%	VUS	chr2	148683674
AD19	MSS	8,95	HCN1	MISSENSE	c.88G>A	p.Ala30Thr	12%	VUS	chr5	45696108
AD19	MSS	8,95	SIRT5	MISSENSE	c.661G>A	p.Val221Met	71%	VUS	chr6	13599307
AD19	MSS	8,95	AHI1	TRUNC	c.3032C>G	p.Ser1011Ter	51%	VUS	chr6	135715991
AD19	MSS	8,95	ARID2	TRUNC	c.1450C>T	p.Gln484Ter	28%	Likely pathogenic	chr12	46233231
AD19	MSS	8,95	ARID2	MISSENSE	c.5333A>G	p.Asn1778Ser	31%	VUS	chr12	46287474
AD19	MSS	8,95	PPFIA2	MISSENSE	c.1252G>A	p.Ala418Thr	26%	VUS	chr12	81768427
AD19	MSS	8,95	PARP4	MISSENSE	c.652T>C	p.Tyr218His	46%	VUS	chr13	25068800
AD20	MSS	15,21	NRAS	MISSENSE	c.182A>G	p.Gln61Arg	39%	Pathogenic	chr1	115256529
AD20	MSS	15,21	TP53	MISSENSE	c.524G>A	p.Arg175His	29%	Pathogenic	chr17	7578406
AD20	MSS	15,21	OR2T4	TRUNC	c.185dup	p.Ile63HisfsTer22	8%	VUS	chr1	248525067
AD20	MSS	15,21	ALK	MISSENSE	c.3350C>G	p.Thr1117Ser	24%	VUS	chr2	29446217
AD20	MSS	15,21	SLC8A1	TRUNC	c.2080G>T	p.Glu694Ter	31%	VUS	chr2	40392083
AD20	MSS	15,21	ADGRL3	MISSENSE	c.808C>T	p.Leu270Phe	17%	VUS	chr4	62598681
AD20	MSS	15,21	FAT1	MISSENSE	c.10003T>G	p.Phe3335Val	9%	VUS	chr4	187531020
AD20	MSS	15,21	TAP2	MISSENSE	c.1856G>A	p.Arg619Gln	5%	VUS	chr6	32797253
AD20	MSS	15,21	RUNDC3B	MISSENSE	c.1025A>G	p.Asn342Ser	27%	VUS	chr7	87436705
AD20	MSS	15,21	KMT2C	MISSENSE	c.1331G>A	p.Arg444Gln	22%	VUS	chr7	151949769
AD20	MSS	15,21	JAK2	MISSENSE	c.1789G>A	p.Ala597Thr	23%	VUS	chr9	5073710
AD20	MSS	15,21	PTEN	MISSENSE	c.454C>G	p.Leu152Val	36%	VUS	chr10	89692970
AD20	MSS	15,21	KMT2D	MISSENSE	c.3890G>A	p.Arg1297His	3%	VUS	chr12	49443481
AD20	MSS	15,21	PPFIA2	MISSENSE	c.2864G>A	p.Arg955Gln	42%	VUS	chr12	81688675
AD20	MSS	15,21	MGA	MISSENSE	c.4043G>A	p.Arg1348Gln	5%	VUS	chr15	42028505
AD20	MSS	15,21	BRIP1	MISSENSE	c.1736G>A	p.Arg579His	5%	VUS	chr17	59858259
AD20	MSS	15,21	AXIN2	TRUNC	c.1092del	p.Val365TrpfsTer10	57%	Likely pathogenic	chr17	63534433
AD20	MSS	15,21	DGCR8	MISSENSE	c.1580G>T	p.Arg527Leu	39%	VUS	chr22	20079467
AD20	MSS	15,21	EP300	MISSENSE	c.5288G>A	p.Arg1763Gln	4%	VUS	chr22	41573003
AD20	MSS	15,21	EP300	MISSENSE	c.7146G>A	p.Met2382Ile	29%	VUS	chr22	41574861
AD20	MSS	15,21	KDM5C	MISSENSE	c.418C>T	p.Arg140Cys	12%	VUS	chrX	53247082
AD20	MSS	15,21	AR	MISSENSE	c.2360G>A	p.Arg787Gln	4%	VUS	chrX	66941716
AD20	MSS	15,21	FAT1	MISSENSE	c.9691G>A	p.Glu3231Lys	4%	VUS	chr4	187532702
AD20	MSS	15,21	KMT2D	MISSENSE	c.14237G>A	p.Arg4746Gln	4%	VUS	chr12	49422858
AD21	MSS	4,76	APC	TRUNC	c.4138_416(	p.Cys1387Ter	30%	Pathogenic	chr5	112175428

AD21	MSS	4,76	APC	TRUNC	c.4323del	p.Pro1442LeufsTer31	23%	Pathogenic	chr5	112175614
AD21	MSS	4,76	KRAS	MISSENSE	c.35G>T	p.Gly12Val	27%	Pathogenic	chr12	25398284
AD21	MSS	4,76	TP53	MISSENSE	c.844C>T	p.Arg282Trp	31%	Pathogenic	chr17	7577094
AD21	MSS	4,76	TP53	MISSENSE	c.524G>A	p.Arg175His	4%	Pathogenic	chr17	7578406
AD21	MSS	4,76	KMT2D	TRUNC	c.13724del	p.Leu4575ProfsTer17	31%	Pathogenic	chr12	49424499
AD21	MSS	4,76	SPAG16	MISSENSE	c.1312G>A	p.Ala438Thr	26%	VUS	chr2	214794781
AD21	MSS	4,76	ADAMTS2	MISSENSE	c.1736C>T	p.Thr579Met	31%	VUS	chr5	178566930
AD21	MSS	4,76	NAV2	MISSENSE	c.317G>A	p.Arg106His	18%	VUS	chr11	19854079
AD21	MSS	4,76	HERC2	MISSENSE	c.10993G>A	p.Val3665Ile	50%	VUS	chr15	28391398
AD21	MSS	4,76	SLX4	MISSENSE	c.4484C>T	p.Ala1495Val	35%	VUS	chr16	3639155
AD21	MSS	4,76	CREBBP	MISSENSE	c.6580C>T	p.Arg2194Trp	39%	VUS	chr16	3778468
AD21	MSS	4,76	CDH1	MISSENSE	c.268C>T	p.Arg90Trp	33%	VUS	chr16	68835677
AD22	MSS	13,32	APC	TRUNC	c.637C>T	p.Arg213Ter	20%	Pathogenic	chr5	112116592
AD22	MSS	13,32	APC	TRUNC	c.4348C>T	p.Arg1450Ter	18%	Pathogenic	chr5	112175639
AD22	MSS	13,32	MAP2K1	MISSENSE	c.171G>T	p.Lys57Asn	23%	Pathogenic	chr15	66727455
AD22	MSS	13,32	TP53	TRUNC	c.880del	p.Glu294SerfsTer51	27%	Likely pathogenic	chr17	7577058
AD22	MSS	13,32	ELF3	TRUNC	c.796dup	p.Ser266LysfsTer35	21%	Likely pathogenic	chr1	201982417
AD22	MSS	13,32	MSH3	MISSENSE	c.1154A>G	p.Asn385Ser	55%	VUS	chr5	79970928
AD22	MSS	13,32	WT1	MISSENSE	c.1088C>T	p.Thr363Met	22%	VUS	chr11	32421519
AD22	MSS	13,32	KMT2A	MISSENSE	c.4778G>A	p.Arg1593His	48%	VUS	chr11	118361992
AD22	MSS	13,32	SMAD4	TRUNC	c.1612G>T	p.Glu538Ter	25%	Likely pathogenic	chr18	48604790
AD22	MSS	13,32	ERCC2	MISSENSE	c.2150C>G	p.Ala717Gly	49%	VUS	chr19	45855507
AD22	MSS	13,32	AMER1	TRUNC	c.1756C>T	p.Arg586Ter	41%	Pathogenic	chrX	63411411
AD23	MSS	2,84	APC	TRUNC	c.2510C>G	p.Ser837Ter	22%	Pathogenic	chr5	112173801
AD23	MSS	2,84	APC	TRUNC	c.4349del	p.Arg1450GlnfsTer23	22%	Pathogenic	chr5	112175640
AD23	MSS	2,84	KMT2C	MISSENSE	c.7826G>A	p.Arg2609Gln	42%	VUS	chr7	151874712
AD23	MSS	2,84	FGF4	MISSENSE	c.2T>C	p.Met1Thr	37%	VUS	chr11	69589851
AD23	MSS	2,84	KRAS	MISSENSE	c.35G>A	p.Gly12Asp	23%	Pathogenic	chr12	25398284
AD23	MSS	2,84	DICER1	MISSENSE	c.3462A>C	p.Arg1154Ser	48%	VUS	chr14	95570271
AD23	MSS	2,84	AHNAK2	MISSENSE	c.15892A>G	p.Arg5298Gly	51%	VUS	chr14	105405896
AD23	MSS	2,84	TP53	TRUNC	c.437G>A	p.Trp146Ter	28%	Pathogenic	chr17	7578493
AD24	MSS	4,78	APC	TRUNC	c.904C>T	p.Arg302Ter	50%	Pathogenic	chr5	112151261
AD24	MSS	4,78	TP53	MISSENSE	c.524G>A	p.Arg175His	32%	Pathogenic	chr17	7578406

AD24	MSS	4,78	ATR	TRUNC	c.6442C>T	p.Arg2148Ter	31%	Likely pathogenic	chr3	142188289
AD24	MSS	4,78	PTEN	TRUNC	c.81T>G	p.Tyr27Ter	32%	Likely pathogenic	chr10	89653783
AD24	MSS	4,78	BRCA1	MISSENSE	c.4993G>A	p.Val1665Met	50%	VUS	chr17	41219706
AD24	MSS	4,78	HEATR6	MISSENSE	c.3005A>G	p.Asn1002Ser	53%	VUS	chr17	58121465
AD24	MSS	4,78	SMAD4	MISSENSE	c.344G>T	p.Cys115Phe	30%	VUS	chr18	48575150
AD24	MSS	4,78	CDHR4	MISSENSE	c.222A>T	p.Gln74His	11%	VUS	chr3	49836698
AD25	MSS	51,89	BRAF	MISSENSE	c.1799T>A	p.Val600Glu	18%	Pathogenic	chr7	140453136
AD25	MSS	51,89	APC	SPLICE	c.1626+2T>C		11%	Pathogenic	chr5	112163705
AD25	MSS	51,89	PTEN	TRUNC	c.1012del	p.Ser338LeufsTer6	20%	Pathogenic	chr10	89720858
AD25	MSS	51,89	PTEN	MISSENSE	c.2T>C	p.Met1?	19%	Pathogenic	chr10	89624228
AD25	MSS	51,89	NBN	TRUNC	c.2051del	p.Asn684IlefsTer25	15%	Pathogenic	chr8	90958387
AD25	MSS	51,89	PIK3R1	TRUNC	c.1761del	p.Gly588ValfsTer7	19%	Likely pathogenic	chr5	67591259
AD25	MSS	51,89	CTCF	TRUNC	c.579del	p.Asp194ThrfsTer28	19%	Likely pathogenic	chr16	67645310
AD25	MSS	51,89	CUL4B	TRUNC	c.27del	p.Ser10ValfsTer32	19%	Likely pathogenic	chrX	119694467
AD25	MSS	51,89	USP9X	TRUNC	c.4104_4105	p.Arg1368SerfsTer2	15%	Likely pathogenic	chrX	41055854
AD25	MSS	51,89	TET2	TRUNC	c.3014delA	p.Lys1005ArgfsTer2	21%	Likely pathogenic	chr4	106158109
AD25	MSS	51,89	BRCA2	MISSENSE	c.7088A>G	p.Tyr2363Cys	23%	VUS	chr13	32929078
AD25	MSS	51,89	CHEK2	MISSENSE	c.337T>C	p.Tyr113His	20%	VUS	chr22	29121338
AD25	MSS	51,89	TP53	MISSENSE	c.847C>T	p.Arg283Cys	19%	VUS	chr17	7577091
AD25	MSS	51,89	ZNF429	TRUNC	c.531del	p.Lys177AsnfsTer12	19%	VUS	chr19	21719381
AD25	MSS	51,89	EPHA2	MISSENSE	c.12G>T	p.Gln4His	22%	VUS	chr1	16482416
AD25	MSS	51,89	C8A	MISSENSE	c.286T>C	p.Cys96Arg	5%	VUS	chr1	57340736
AD25	MSS	51,89	LEPR	MISSENSE	c.3082T>G	p.Ser1028Ala	21%	VUS	chr1	66102282
AD25	MSS	51,89	HIPK1	MISSENSE	c.2042C>T	p.Pro681Leu	24%	VUS	chr1	114504999
AD25	MSS	51,89	NOTCH2	MISSENSE	c.5654C>T	p.Ala1885Val	26%	VUS	chr1	120462062
AD25	MSS	51,89	ZDBF2	MISSENSE	c.5024G>A	p.Arg1675Gln	21%	VUS	chr2	207174276
AD25	MSS	51,89	ERBB4	MISSENSE	c.2549T>C	p.Leu850Ser	20%	VUS	chr2	212295764
AD25	MSS	51,89	CNTN6	MISSENSE	c.2710A>G	p.Ser904Gly	7%	VUS	chr3	1443122
AD25	MSS	51,89	FAT1	MISSENSE	c.10903A>G	p.Thr3635Ala	53%	VUS	chr4	187524777
AD25	MSS	51,89	APC	MISSENSE	c.7649A>C	p.Glu2550Ala	22%	VUS	chr5	112178940
AD25	MSS	51,89	SIMC1	MISSENSE	c.683G>A	p.Cys228Tyr	21%	VUS	chr5	175717210
AD25	MSS	51,89	H3C2	MISSENSE	c.155T>A	p.Ile52Asn	20%	VUS	chr6	26032134
AD25	MSS	51,89	DAXX	MISSENSE	c.779G>A	p.Arg260His	18%	VUS	chr6	33288773

AD25	MSS	51,89	FYN	MISSENSE	c.1526A>G	p.Glu509Gly	18%	VUS	chr6	111983030
AD25	MSS	51,89	RNF217	MISSENSE	c.788G>A	p.Cys263Tyr	17%	VUS	chr6	125284478
AD25	MSS	51,89	ETV1	MISSENSE	c.237G>T	p.Leu79Phe	17%	VUS	chr7	13978870
AD25	MSS	51,89	EGFR	MISSENSE	c.2164G>T	p.Ala722Ser	16%	VUS	chr7	55241716
AD25	MSS	51,89	TSC1	MISSENSE	c.3224C>A	p.Pro1075His	5%	VUS	chr9	135771893
AD25	MSS	51,89	LARP4B	MISSENSE	c.703C>T	p.Arg235Cys	14%	VUS	chr10	882390
AD25	MSS	51,89	NPY4R	MISSENSE	c.722T>C	p.Leu241Pro	8%	VUS	chr10	47087505
AD25	MSS	51,89	OR5F1	MISSENSE	c.254T>C	p.Leu85Ser	48%	VUS	chr11	55761848
AD25	MSS	51,89	CCND1	MISSENSE	c.406G>A	p.Glu136Lys	20%	VUS	chr11	69458006
AD25	MSS	51,89	FGF4	MISSENSE	c.169C>T	p.Arg57Cys	19%	VUS	chr11	69589684
AD25	MSS	51,89	FGF4	MISSENSE	c.155C>T	p.Ala52Val	6%	VUS	chr11	69589698
AD25	MSS	51,89	STAT6	MISSENSE	c.1750C>A	p.Pro584Thr	4%	VUS	chr12	57493218
AD25	MSS	51,89	TBX3	MISSENSE	c.1328C>T	p.Ala443Val	18%	VUS	chr12	115112352
AD25	MSS	51,89	RNASEH2B	MISSENSE	c.240A>T	p.Gln80His	20%	VUS	chr13	51503714
AD25	MSS	51,89	SYT16	MISSENSE	c.1534G>A	p.Gly512Arg	19%	VUS	chr14	62551013
AD25	MSS	51,89	IGF1R	MISSENSE	c.2000A>C	p.Lys667Thr	51%	VUS	chr15	99459904
AD25	MSS	51,89	CREBBP	MISSENSE	c.4671A>T	p.Glu1557Asp	22%	VUS	chr16	3786094
AD25	MSS	51,89	CTCF	MISSENSE	c.1060T>C	p.Ser354Pro	19%	VUS	chr16	67650755
AD25	MSS	51,89	ZFHX3	MISSENSE	c.4517C>T	p.Thr1506Met	18%	VUS	chr16	72832064
AD25	MSS	51,89	ZFHX3	MISSENSE	c.641C>T	p.Ala214Val	49%	VUS	chr16	72993404
AD25	MSS	51,89	NF1	MISSENSE	c.7404G>A	p.Met2468Ile	19%	VUS	chr17	29677283
AD25	MSS	51,89	ERBB2	MISSENSE	c.703T>C	p.Cys235Arg	20%	VUS	chr17	37866398
AD25	MSS	51,89	RPS6KB1	MISSENSE	c.1198C>T	p.Leu400Phe	20%	VUS	chr17	58018275
AD25	MSS	51,89	PPM1D	TRUNC	c.1349del	p.Leu450Ter	21%	Likely pathogenic	chr17	58740439
AD25	MSS	51,89	PRKAR1A	MISSENSE	c.1079G>A	p.Gly360Asp	17%	VUS	chr17	66526523
AD25	MSS	51,89	SMAD4	MISSENSE	c.1109T>C	p.Val370Ala	26%	VUS	chr18	48591946
AD25	MSS	51,89	NOTCH3	MISSENSE	c.6304G>A	p.Val2102Met	13%	VUS	chr19	15272135
AD25	MSS	51,89	ZNF429	MISSENSE	c.988A>G	p.Ser330Gly	19%	VUS	chr19	21719843
AD25	MSS	51,89	CCNE1	MISSENSE	c.718C>T	p.Arg240Cys	18%	VUS	chr19	30312915
AD25	MSS	51,89	KMT2B	MISSENSE	c.4081G>A	p.Asp1361Asn	20%	VUS	chr19	36218134
AD25	MSS	51,89	KMT2B	MISSENSE	c.6958G>T	p.Gly2320Trp	21%	VUS	chr19	36224408
AD25	MSS	51,89	ERCC2	MISSENSE	c.1598A>G	p.Asp533Gly	7%	VUS	chr19	45858055
AD25	MSS	51,89	ARHGAP35	MISSENSE	c.2231G>A	p.Arg744His	17%	VUS	chr19	47424163

AD25	MSS	51,89	PRND	MISSENSE	c.467G>A	p.Arg156Gln	8%	VUS	chr20	4705664
AD25	MSS	51,89	CBLL2	MISSENSE	c.560T>A	p.Met187Lys	19%	VUS	chrX	22291668
AD25	MSS	51,89	USP9X	MISSENSE	c.3353T>C	p.Leu1118Ser	19%	VUS	chrX	41043723
AD25	MSS	51,89	KDM6A	MISSENSE	c.1340A>G	p.His426Arg	20%	VUS	chrX	44919936
AD25	MSS	51,89	KDM6A	MISSENSE	c.2846G>A	p.Arg949His	10%	VUS	chrX	44937658
AD25	MSS	51,89	AMER1	MISSENSE	c.2383T>G	p.Ser795Ala	20%	VUS	chrX	63410784
AD25	MSS	51,89	ATRX	MISSENSE	c.3300T>A	p.Ser1100Arg	20%	VUS	chrX	76937448
AD25	MSS	51,89	DDR1	SPLICE	c.418-1G>T		20%	VUS	chr6	30858749
AD25	MSS	51,89	KELL	MISSENSE	c.238T>C	p.Ser80Pro	3%	VUS	chr7	142658177
AD26	MSS	7,72	KRAS	MISSENSE	c.35G>T	p.Gly12Val	17%	Pathogenic	chr12	25398284
AD26	MSS	7,72	SMAD4	TRUNC	c.726_729de	p.Gly243LeufsTer92	14%	Pathogenic	chr18	48584553
AD26	MSS	7,72	PIK3CA	MISSENSE	c.323G>A	p.Arg108His	15%	Likely pathogenic	chr3	178916936
AD26	MSS	7,72	TP53	MISSENSE	c.536A>G	p.His179Arg	10%	Likely pathogenic	chr17	7578394
AD26	MSS	7,72	TET2	MISSENSE	c.5633G>A	p.Arg1878His	19%	VUS	chr4	106197300
AD26	MSS	7,72	TMEM132D	MISSENSE	c.2068A>C	p.Ile690Leu	14%	VUS	chr12	129563126
AD26	MSS	7,72	CSNK1A1L	MISSENSE	c.194C>T	p.Thr65Met	6%	VUS	chr13	37679200
AD26	MSS	7,72	TSC2	MISSENSE	c.694C>T	p.Pro232Ser	55%	VUS	chr16	2106690
AD27	MSS	6,64	PDGFRB	MISSENSE	c.1853C>T	p.Thr618Met	58%	VUS	chr5	149504349
AD27	MSS	6,64	ARID1B	INFRAME	c.278_298de	p.His93_His99del	81%	VUS	chr6	157099327
AD27	MSS	6,64	ADAM18	MISSENSE	c.1515A>G	p.Ile505Met	45%	VUS	chr8	39525705
AD27	MSS	6,64	RB1	MISSENSE	c.2318C>T	p.Ser773Phe	5%	VUS	chr13	49039240
AD27	MSS	6,64	COL4A2	TRUNC	c.1571del	p.Gly524AlafsTer47	51%	VUS	chr13	111111255
AD27	MSS	6,64	BLM	MISSENSE	c.842A>C	p.His281Pro	54%	VUS	chr15	91295059
AD27	MSS	6,64	DSC3	MISSENSE	c.526C>T	p.Arg176Cys	5%	VUS	chr18	28605830
AD27	MSS	6,64	TP53	MISSENSE	c.761T>G	p.Ile254Ser	3%	VUS	chr17	7577520
AD28	MSS	6,6	APC	TRUNC	c.3927_3931	p.Glu1309AspfsTer4	46%	Pathogenic	chr5	112175212
AD28	MSS	6,6	TP53	TRUNC	c.882del	p.Glu294AspfsTer51	65%	Likely pathogenic	chr17	7577056
AD28	MSS	6,6	SMARCB1	MISSENSE	c.1129C>T	p.Arg377Cys	69%	Likely pathogenic	chr22	24176338
AD28	MSS	6,6	TSC2	MISSENSE	c.3730C>A	p.His1244Asn	30%	VUS	chr16	2131715
AD28	MSS	6,6	PIK3R1	INFRAME	c.1703_1714	p.Pro568_Ile571del	56%	VUS	chr5	67591109
AD28	MSS	6,6	IP6K2	MISSENSE	c.640G>A	p.Glu214Lys	42%	VUS	chr3	48727111
AD28	MSS	6,6	ATR	MISSENSE	c.2653G>A	p.Val885Ile	31%	VUS	chr3	142272221
AD28	MSS	6,6	TCOF1	MISSENSE	c.1848C>G	p.Asp616Glu	79%	VUS	chr5	149755427

AD28	MSS	6,6	FAM120B	MISSENSE	c.1118C>G	p.Pro373Arg	35%	VUS	chr6	170627596
AD28	MSS	6,6	GLI3	MISSENSE	c.3985G>A	p.Gly1329Ser	6%	VUS	chr7	42004686
AD29	MSS	14,21	NOTCH2	MISSENSE	c.1033G>A	p.Ala345Thr	25%	VUS	chr1	120512209
AD29	MSS	14,21	CRIM1	TRUNC	c.1113T>A	p.Cys371Ter	20%	VUS	chr2	36704153
AD29	MSS	14,21	PIK3CA	MISSENSE	c.1634A>C	p.Glu545Ala	33%	Pathogenic	chr3	178936092
AD29	MSS	14,21	PDGFRA	MISSENSE	c.1425G>T	p.Glu475Asp	50%	VUS	chr4	55139764
AD29	MSS	14,21	APC	MISSENSE	c.1214G>A	p.Arg405Gln	20%	VUS	chr5	112154943
AD29	MSS	14,21	APC	TRUNC	c.2413C>T	p.Arg805Ter	28%	Pathogenic	chr5	112173704
AD29	MSS	14,21	APC	TRUNC	c.4099C>T	p.Gln1367Ter	29%	Likely pathogenic	chr5	112175390
AD29	MSS	14,21	AHI1	MISSENSE	c.3055T>C	p.Ser1019Pro	20%	VUS	chr6	135715968
AD29	MSS	14,21	TCF7L2	MISSENSE	c.1258C>T	p.Arg420Trp	27%	VUS	chr10	114912188
AD29	MSS	14,21	MEN1	MISSENSE	c.1481C>T	p.Pro494Leu	48%	VUS	chr11	64572158
AD29	MSS	14,21	KRAS	MISSENSE	c.34G>A	p.Gly12Ser	29%	Pathogenic	chr12	25398285
AD29	MSS	14,21	ARID2	TRUNC	c.5030_5031	p.Gln1678ThrfsTer12	15%	Likely pathogenic	chr12	46285671
AD29	MSS	14,21	TP53	TRUNC	c.626_627de	p.Arg209LysfsTer6	11%	Pathogenic	chr17	7578222
AD29	MSS	14,21	PTPRT	TRUNC	c.3061C>T	p.Arg1021Ter	29%	Likely pathogenic	chr20	40743943
AD29	MSS	14,21	ATRX	MISSENSE	c.3G>T	p.Met1Ile	4%	VUS	chrX	77041485
AD30	MSS	4,72	KRAS	MISSENSE	c.34G>T	p.Gly12Cys	17%	Pathogenic	chr12	25398285
AD30	MSS	4,72	TP53	MISSENSE	c.743G>A	p.Arg248Gln	22%	Pathogenic	chr17	7577538
AD30	MSS	4,72	APC	TRUNC	c.2752G>T	p.Glu918Ter	13%	Likely pathogenic	chr5	112174043
AD30	MSS	4,72	APC	TRUNC	c.4359del	p.Asn1455IlefsTer18	18%	Likely pathogenic	chr5	112175650
AD30	MSS	4,72	USF3	MISSENSE	c.3208G>A	p.Asp1070Asn	49%	VUS	chr3	113377321
AD30	MSS	4,72	APC	MISSENSE	c.3341G>A	p.Arg1114Gln	52%	VUS	chr5	112174632
AD30	MSS	4,72	POM121L1	MISSENSE	c.713C>T	p.Pro238Leu	14%	VUS	chr7	53104077
AD30	MSS	4,72	DCAF4L2	MISSENSE	c.671G>C	p.Ser224Thr	50%	VUS	chr8	88885529
AD30	MSS	4,72	RECQL4	MISSENSE	c.308C>T	p.Pro103Leu	53%	VUS	chr8	145742480
AD30	MSS	4,72	ATM	MISSENSE	c.7993C>T	p.Pro2665Ser	18%	VUS	chr11	108204678
AD30	MSS	4,72	TBX3	MISSENSE	c.1893C>G	p.Asp631Glu	44%	VUS	chr12	115109925
AD30	MSS	4,72	POLE	MISSENSE	c.5200C>G	p.Leu1734Val	50%	VUS	chr12	133218411
AD30	MSS	4,72	KLF5	TRUNC	c.679dup	p.His227ProfsTer27	15%	VUS	chr13	73636415
AD30	MSS	4,72	AXIN2	MISSENSE	c.1184T>C	p.Leu395Pro	52%	VUS	chr17	63534337
AD30	MSS	4,72	CIC	MISSENSE	c.2593G>T	p.Ala865Ser	51%	VUS	chr19	42778528
AD31	MSS	4,86	KRAS	MISSENSE	c.38G>A	p.Gly13Asp	30%	Pathogenic	chr12	25398281

AD31	MSS	4,86	APC	TRUNC	c.3291dup	p.Cys1098MetfsTer21	30%	Likely pathogenic	chr5	112174581
AD31	MSS	4,86	APC	TRUNC	c.4199C>A	p.Ser1400Ter	32%	Likely pathogenic	chr5	112175490
AD31	MSS	4,86	GLI3	MISSENSE	c.1024A>G	p.Ile342Val	49%	VUS	chr7	42079641
AD31	MSS	4,86	TCF7L2	MISSENSE	c.1411C>T	p.Arg471Cys	60%	VUS	chr10	114925333
AD31	MSS	4,86	TYR	TRUNC	c.556dup	p.Asp186GlyfsTer8	8%	VUS	chr11	88911676
AD31	MSS	4,86	PDE1A	TRUNC	c.219_220in	p.Leu74SerfsTer5	5%	VUS	chr2	183105016
AD31	MSS	4,86	COL4A2	MISSENSE	c.1505A>G	p.Lys502Arg	36%	VUS	chr13	111111190
AD31	MSS	4,86	RAD51B	MISSENSE	c.553T>G	p.Cys185Gly	50%	VUS	chr14	68352686
AD31	MSS	4,86	HAS3	MISSENSE	c.206G>A	p.Arg69His	15%	VUS	chr16	69143504
AD31	MSS	4,86	TP53	MISSENSE	c.613T>C	p.Tyr205His	57%	VUS	chr17	7578236
AD32	MSS	4,77	PIK3CA	MISSENSE	c.263G>A	p.Arg88Gln	38%	Pathogenic	chr3	178916876
AD32	MSS	4,77	TP53	MISSENSE	c.329G>T	p.Arg110Leu	57%	Pathogenic	chr17	7579358
AD32	MSS	4,77	SPEG	MISSENSE	c.3748G>A	p.Val1250Met	60%	VUS	chr2	220336622
AD32	MSS	4,77	PRDM9	MISSENSE	c.1759C>T	p.Arg587Trp	57%	VUS	chr5	23526956
AD32	MSS	4,77	HDAC9	TRUNC	c.1987C>T	p.Arg663Ter	35%	VUS	chr7	18788705
AD32	MSS	4,77	CSMD3	MISSENSE	c.3932C>G	p.Thr1311Ser	44%	VUS	chr8	113585840
AD32	MSS	4,77	CSMD3	MISSENSE	c.2729G>C	p.Gly910Ala	34%	VUS	chr8	113678593
AD32	MSS	4,77	CDKN2A	MISSENSE	c.17G>A	p.Gly6Glu	37%	VUS	chr9	21974810
AD32	MSS	4,77	FANCM	MISSENSE	c.2190A>T	p.Gln730His	48%	VUS	chr14	45642287
AD32	MSS	4,77	FANCI	MISSENSE	c.3113C>T	p.Ser1038Leu	21%	VUS	chr15	89848400
AD32	MSS	4,77	RPA1	MISSENSE	c.1681G>C	p.Val561Leu	31%	VUS	chr17	1798324
AD33	MSS	6,64	KRAS	MISSENSE	c.183A>T	p.Gln61His	24%	Pathogenic	chr12	25380275
AD33	MSS	6,64	TP53	MISSENSE	c.713G>T	p.Cys238Phe	7%	Pathogenic	chr17	7577568
AD33	MSS	6,64	AXIN2	TRUNC	c.724_727de	p.Lys242PhefsTer13	53%	Likely pathogenic	chr17	63554012
AD33	MSS	6,64	NTRK1	MISSENSE	c.1841G>A	p.Gly614Glu	3%	VUS	chr1	156848949
AD33	MSS	6,64	CASR	MISSENSE	c.1618T>C	p.Ser540Pro	23%	VUS	chr3	122000969
AD33	MSS	6,64	NSUN7	MISSENSE	c.1834C>T	p.His612Tyr	60%	VUS	chr4	40810633
AD33	MSS	6,64	ACSL1	MISSENSE	c.1210G>A	p.Gly404Ser	35%	VUS	chr4	185687829
AD33	MSS	6,64	KMT2C	MISSENSE	c.994G>A	p.Asp332Asn	6%	VUS	chr7	151970808
AD33	MSS	6,64	CSMD3	MISSENSE	c.10387G>C	p.Val3463Leu	44%	VUS	chr8	113256638
AD33	MSS	6,64	NPY4R	MISSENSE	c.632G>A	p.Arg211His	28%	VUS	chr10	47087415
AD33	MSS	6,64	ZFHX3	MISSENSE	c.9587C>T	p.Pro3196Leu	28%	VUS	chr16	72822588
AD33	MSS	6,64	MAP2K4	TRUNC	c.786del	p.Asp263MetfsTer13	4%	Likely pathogenic	chr17	12016650

AD33	MSS	6,64	BRIP1	MISSENSE	c.1899C>G	p.Ile633Met	25%	VUS	chr17	59857658
AD33	MSS	6,64	NWD1	MISSENSE	c.208G>A	p.Gly70Ser	49%	VUS	chr19	16855241
AD34	MSS	5,71	BRAF	MISSENSE	c.1799T>A	p.Val600Glu	27%	Pathogenic	chr7	140453136
AD34	MSS	5,71	TP53	MISSENSE	c.725G>A	p.Cys242Tyr	31%	Pathogenic	chr17	7577556
AD34	MSS	5,71	NOTCH2	MISSENSE	c.3467A>G	p.Asn1156Ser	49%	VUS	chr1	120479960
AD34	MSS	5,71	NOTCH4	MISSENSE	c.1609T>G	p.Cys537Gly	32%	VUS	chr6	32185787
AD34	MSS	5,71	GLI3	MISSENSE	c.2836C>G	p.Pro946Ala	17%	VUS	chr7	42005835
AD34	MSS	5,71	XRCC2	MISSENSE	c.283A>G	p.Ile95Val	45%	VUS	chr7	152346287
AD34	MSS	5,71	HNF1A	MISSENSE	c.901G>A	p.Ala301Thr	48%	VUS	chr12	121432154
AD34	MSS	5,71	MTUS2	MISSENSE	c.2749G>A	p.Gly917Ser	39%	VUS	chr13	29855915
AD34	MSS	5,71	CREBBP	MISSENSE	c.4415G>T	p.Trp1472Leu	11%	VUS	chr16	3786796
AD34	MSS	5,71	ZNF568	MISSENSE	c.1866G>T	p.Lys622Asn	11%	VUS	chr19	37488459
AD35	H-MSI	32,83	ERRFI1	SPLICE	c.203-2A>G		6%	VUS	chr1	8074458
AD35	H-MSI	32,83	SPEN	MISSENSE	c.2602G>T	p.Ala868Ser	9%	VUS	chr1	16255337
AD35	H-MSI	32,83	SPEN	MISSENSE	c.5701C>T	p.Arg1901Cys	9%	VUS	chr1	16258436
AD35	H-MSI	32,83	SPEN	MISSENSE	c.9469G>A	p.Glu3157Lys	19%	VUS	chr1	16262204
AD35	H-MSI	32,83	NOTCH2	MISSENSE	c.5612C>T	p.Ala1871Val	10%	VUS	chr1	120462104
AD35	H-MSI	32,83	NTRK1	MISSENSE	c.1748G>A	p.Arg583His	7%	VUS	chr1	156846307
AD35	H-MSI	32,83	BRINP3	MISSENSE	c.1357G>A	p.Ala453Thr	9%	VUS	chr1	190068092
AD35	H-MSI	32,83	PARP1	MISSENSE	c.1305G>T	p.Glu435Asp	7%	VUS	chr1	226567861
AD35	H-MSI	32,83	OR2T33	TRUNC	c.906G>A	p.Trp302Ter	6%	VUS	chr1	248436211
AD35	H-MSI	32,83	ASXL2	MISSENSE	c.1289A>C	p.Glu430Ala	10%	VUS	chr2	25973136
AD35	H-MSI	32,83	PDE1A	MISSENSE	c.1429A>G	p.Thr477Ala	5%	VUS	chr2	183050754
AD35	H-MSI	32,83	TGFBR2	MISSENSE	c.760C>T	p.Arg254Cys	19%	VUS	chr3	30713435
AD35	H-MSI	32,83	PIK3CA	MISSENSE	c.3140A>G	p.His1047Arg	13%	Pathogenic	chr3	178952085
AD35	H-MSI	32,83	FBXW7	MISSENSE	c.1394G>A	p.Arg465His	9%	Likely pathogenic	chr4	153249384
AD35	H-MSI	32,83	FBXW7	MISSENSE	c.1393C>T	p.Arg465Cys	10%	Likely pathogenic	chr4	153249385
AD35	H-MSI	32,83	GABRR2	TRUNC	c.1261A>T	p.Arg421Ter	21%	VUS	chr6	89967526
AD35	H-MSI	32,83	ARID1B	MISSENSE	c.6089G>C	p.Arg2030Pro	52%	VUS	chr6	157528244
AD35	H-MSI	32,83	CARD11	MISSENSE	c.1726C>T	p.Arg576Cys	21%	VUS	chr7	2968260
AD35	H-MSI	32,83	HECW1	MISSENSE	c.1573C>T	p.Arg525Trp	12%	VUS	chr7	43484344
AD35	H-MSI	32,83	BRAF	MISSENSE	c.1799T>A	p.Val600Glu	22%	Pathogenic	chr7	140453136
AD35	H-MSI	32,83	KEL	MISSENSE	c.1211G>A	p.Arg404His	8%	VUS	chr7	142643397

AD35	H-MSI	32,83	KMT2C	MISSENSE	c.12626G>A	p.Gly4209Asp	17%	VUS	chr7	151848567
AD35	H-MSI	32,83	CSMD1	MISSENSE	c.4969G>A	p.Val1657Ile	12%	VUS	chr8	3063041
AD35	H-MSI	32,83	RUNX1T1	MISSENSE	c.304G>A	p.Val102Met	9%	VUS	chr8	93029553
AD35	H-MSI	32,83	ARMC4	MISSENSE	c.1415C>T	p.Ala472Val	17%	VUS	chr10	28233863
AD35	H-MSI	32,83	FGFR2	MISSENSE	c.1990C>T	p.Arg664Trp	7%	VUS	chr10	123246935
AD35	H-MSI	32,83	ATM	MISSENSE	c.5295G>T	p.Gln1765His	6%	VUS	chr11	108172492
AD35	H-MSI	32,83	SORL1	TRUNC	c.3805C>T	p.Gln1269Ter	13%	Likely pathogenic	chr11	121457029
AD35	H-MSI	32,83	RAD52	MISSENSE	c.178G>T	p.Gly60Cys	19%	VUS	chr12	1040394
AD35	H-MSI	32,83	CDKN1B	TRUNC	c.444C>A	p.Cys148Ter	23%	Likely pathogenic	chr12	12871217
AD35	H-MSI	32,83	SLCO1B3	MISSENSE	c.293G>T	p.Gly98Val	18%	VUS	chr12	21011439
AD35	H-MSI	32,83	KMT2D	MISSENSE	c.16411A>G	p.Arg5471Gly	5%	VUS	chr12	49416064
AD35	H-MSI	32,83	KMT2D	MISSENSE	c.961C>T	p.Arg321Trp	6%	VUS	chr12	49446849
AD35	H-MSI	32,83	KMT2D	TRUNC	c.231del	p.His77GlnfsTer53	6%	Likely pathogenic	chr12	49448480
AD35	H-MSI	32,83	FGF9	MISSENSE	c.308G>A	p.Gly103Asp	7%	VUS	chr13	22255211
AD35	H-MSI	32,83	B2M	SPLICE	c.68-2A>G		12%	VUS	chr15	45007619
AD35	H-MSI	32,83	B2M	TRUNC	c.194_195de	p.Arg65AsnfsTer2	7%	Likely pathogenic	chr15	45007741
AD35	H-MSI	32,83	B2M	TRUNC	c.204del	p.Val69TrpfsTer34	15%	Likely pathogenic	chr15	45007753
AD35	H-MSI	32,83	AXIN1	MISSENSE	c.1958C>A	p.Ser653Tyr	20%	VUS	chr16	343716
AD35	H-MSI	32,83	TSC2	MISSENSE	c.1748C>T	p.Ala583Val	48%	VUS	chr16	2120488
AD35	H-MSI	32,83	TSC2	MISSENSE	c.2297T>C	p.Val766Ala	48%	VUS	chr16	2122926
AD35	H-MSI	32,83	CDH1	MISSENSE	c.1571G>A	p.Arg524Gln	12%	VUS	chr16	68853188
AD35	H-MSI	32,83	AXIN2	MISSENSE	c.1087A>G	p.Thr363Ala	9%	VUS	chr17	63534434
AD35	H-MSI	32,83	JAK3	MISSENSE	c.2869G>C	p.Val957Leu	21%	VUS	chr19	17942146
AD35	H-MSI	32,83	ASXL1	MISSENSE	c.185C>G	p.Ser62Cys	19%	VUS	chr20	30956859
AD35	H-MSI	32,83	BCOR	MISSENSE	c.1306G>A	p.Val436Ile	21%	VUS	chrX	39933293
AD35	H-MSI	32,83	USP9X	MISSENSE	c.1478G>A	p.Arg493His	22%	VUS	chrX	41007680
AD35	H-MSI	32,83	DIPK2B	MISSENSE	c.427C>T	p.Arg143Cys	13%	VUS	chrX	45051067
AD35	H-MSI	32,83	AMER1	MISSENSE	c.2861G>T	p.Gly954Val	14%	VUS	chrX	63410306
AD35	H-MSI	32,83	AMER1	MISSENSE	c.1423A>G	p.Thr475Ala	9%	VUS	chrX	63411744
AD35	H-MSI	32,83	AMER1	MISSENSE	c.714A>C	p.Lys238Asn	8%	VUS	chrX	63412453
AD36	MSS	2,84	BRAF	MISSENSE	c.1919T>A	p.Val600Glu	50%	Pathogenic	chr7	140453136
AD36	MSS	2,84	TP53	MISSENSE	c.524G>A	p.Arg175His	50%	Pathogenic	chr17	7578406
AD36	MSS	2,84	SMAD4	MISSENSE	c.1081C>T	p.Arg361Cys	46%	Pathogenic	chr18	48591918

AD36	MSS	2,84	MARCO	MISSENSE	c.1551G>T	p.Glu517Asp	48%	VUS	chr2	119752084
AD36	MSS	2,84	SDHA	MISSENSE	c.512G>A	p.Arg171His	5%	VUS	chr5	226053
AD36	MSS	2,84	ARID1B	MISSENSE	c.116C>T	p.Ser39Phe	69%	VUS	chr6	157099179
AD37	H-MSI	18,95	MLH1	MISSENSE	c.350C>T	p.Thr117Met	48%	Pathogenic	chr3	37045935
AD37	H-MSI	18,95	APC	TRUNC	c.637C>T	p.Arg213Ter	19%	Pathogenic	chr5	112116592
AD37	H-MSI	18,95	KDM6A	TRUNC	c.1834C>T	p.Arg612Ter	32%	Pathogenic	chrX	44922973
AD37	H-MSI	18,95	ERBB3	MISSENSE	c.310G>A	p.Val104Met	33%	Likely pathogenic	chr12	56478854
AD37	H-MSI	18,95	SPEN	TRUNC	c.2417_2418	p.Arg807AspfsTer3	19%	Likely pathogenic	chr1	16255142
AD37	H-MSI	18,95	CDH1	TRUNC	c.885_888du	p.Asn297LeufsTer15	15%	Likely pathogenic	chr16	68845637
AD37	H-MSI	18,95	PPFIA2	TRUNC	c.3084G>A	p.Trp1028Ter	15%	VUS	chr12	81675164
AD37	H-MSI	18,95	CNTNAP4	TRUNC	c.436C>T	p.Arg146Ter	16%	VUS	chr16	76461469
AD37	H-MSI	18,95	ARHGAP35	TRUNC	c.4335delC	p.Gly1446AlafsTer353	19%	Likely pathogenic	chr19	47503774
AD37	H-MSI	18,95	SLC25A17	TRUNC	c.107_109de	p.Arg36GlnfsTer36	50%	VUS	chr22	41195033
AD37	H-MSI	18,95	TGFBR2	SPLICE	c.454+2T>C	intronic variant	12%	VUS	chr3	30691954
AD37	H-MSI	18,95	C8B	MISSENSE	c.1445C>T	p.Ser482Phe	45%	VUS	chr1	57399115
AD37	H-MSI	18,95	SDHC	MISSENSE	c.206T>A	p.Ile69Asn	21%	VUS	chr1	161310410
AD37	H-MSI	18,95	AKT3	MISSENSE	c.505G>A	p.Ala169Thr	4%	VUS	chr1	243800969
AD37	H-MSI	18,95	THADA	MISSENSE	c.1415C>G	p.Ala472Gly	45%	VUS	chr2	43801789
AD37	H-MSI	18,95	MSH6	MISSENSE	c.3079G>C	p.Val1027Leu	46%	VUS	chr2	48028201
AD37	H-MSI	18,95	CASP8	MISSENSE	c.446T>G	p.Leu149Arg	18%	VUS	chr2	202131478
AD37	H-MSI	18,95	ERBB4	MISSENSE	c.2717A>G	p.Tyr906Cys	16%	VUS	chr2	212293135
AD37	H-MSI	18,95	DCAF4L1	MISSENSE	c.298G>A	p.Gly100Ser	9%	VUS	chr4	41984107
AD37	H-MSI	18,95	SDHA	MISSENSE	c.970G>A	p.Glu324Lys	19%	VUS	chr5	233666
AD37	H-MSI	18,95	CDH10	MISSENSE	c.1000A>G	p.Lys334Glu	48%	VUS	chr5	24511438
AD37	H-MSI	18,95	HDAC2	MISSENSE	c.1110T>A	p.Asn370Lys	15%	VUS	chr6	114265556
AD37	H-MSI	18,95	LATS1	MISSENSE	c.1504C>T	p.Arg502Cys	17%	VUS	chr6	150004721
AD37	H-MSI	18,95	HDAC9	MISSENSE	c.2134G>A	p.Gly712Arg	13%	VUS	chr7	18801870
AD37	H-MSI	18,95	PTCH1	MISSENSE	c.3422C>T	p.Ala1141Val	17%	VUS	chr9	98215787
AD37	H-MSI	18,95	NOTCH1	MISSENSE	c.4793G>A	p.Arg1598His	12%	VUS	chr9	139399350
AD37	H-MSI	18,95	LARP4B	MISSENSE	c.1046C>A	p.Pro349His	3%	VUS	chr10	875404
AD37	H-MSI	18,95	OR5F1	MISSENSE	c.383G>A	p.Arg128His	20%	VUS	chr11	55761719
AD37	H-MSI	18,95	TBX3	MISSENSE	c.1402G>A	p.Ala468Thr	18%	VUS	chr12	115112278
AD37	H-MSI	18,95	OR4M2	MISSENSE	c.611G>A	p.Gly204Asp	29%	VUS	chr15	22369186

AD37	H-MSI	18,95	CDH1	MISSENSE	c.466T>C	p.Trp156Arg	19%	VUS	chr16	68842405
AD37	H-MSI	18,95	ERBB2	MISSENSE	c.661G>A	p.Ala221Thr	5%	VUS	chr17	37866356
AD37	H-MSI	18,95	RARA	MISSENSE	c.833C>T	p.Thr278Met	19%	VUS	chr17	38510579
AD37	H-MSI	18,95	SRC	MISSENSE	c.763A>T	p.Thr255Ser	17%	VUS	chr20	36026161
AD37	H-MSI	18,95	PHF6	MISSENSE	c.488G>A	p.Arg163His	39%	VUS	chrX	133547590
AD37	H-MSI	18,95	EPHA2	MISSENSE	c.2T>C	p.Met1?	24%	VUS	chr1	16482426
AD37	H-MSI	18,95	ZMYM3	MISSENSE	c.2218C>T	p.Arg740Cys	3%	VUS	chrX	70467291
AD38	MSS	5,68	PIK3CA	MISSENSE	c.1624G>A	p.Glu542Lys	5%	Pathogenic	chr3	178936082
AD38	MSS	5,68	PIK3CA	MISSENSE	c.1633G>A	p.Glu545Lys	5%	Pathogenic	chr3	178936091
AD38	MSS	5,68	PIK3CA	MISSENSE	c.3140A>T	p.His1047Leu	2%	Pathogenic	chr3	178952085
AD38	MSS	5,68	APC	TRUNC	c.4348C>T	p.Arg1450Ter	10%	Pathogenic	chr5	112175639
AD38	MSS	5,68	KRAS	MISSENSE	c.34G>T	p.Gly12Cys	21%	Pathogenic	chr12	25398285
AD38	MSS	5,68	PIK3CA	MISSENSE	c.278G>T	p.Arg93Leu	5%	Likely pathogenic	chr3	178916891
AD38	MSS	5,68	APC	TRUNC	c.2413C>T	p.Arg805Ter	26%	Likely pathogenic	chr5	112173704
AD38	MSS	5,68	SOX9	TRUNC	c.788delG	p.Gly263AlafsTer16	39%	Likely pathogenic	chr17	70119780
AD38	MSS	5,68	KDM6A	TRUNC	c.1866_1867	p.Asn623GlnfsTer3	38%	Likely pathogenic	chrX	44923005
AD38	MSS	5,68	DNMT3A	MISSENSE	c.148C>T	p.Arg50Trp	6%	VUS	chr2	25523037
AD38	MSS	5,68	SETD2	MISSENSE	c.4627C>T	p.Arg1543Trp	5%	VUS	chr3	47155454
AD38	MSS	5,68	POT1	MISSENSE	c.1370G>A	p.Gly457Glu	3%	VUS	chr7	124475468
AD38	MSS	5,68	CSMD3	TRUNC	c.7324C>T	p.Arg2442Ter	4%	VUS	chr8	113331102
AD38	MSS	5,68	CCND2	MISSENSE	c.842C>G	p.Pro281Arg	21%	VUS	chr12	4409147
AD38	MSS	5,68	FANCA	MISSENSE	c.2129G>A	p.Arg710Lys	5%	VUS	chr16	89838108
AD38	MSS	5,68	RBM10	MISSENSE	c.305G>A	p.Arg102Gln	4%	VUS	chrX	47028806
AD38	MSS	5,68	AMER1	TRUNC	c.1627G>T	p.Glu543Ter	42%	Pathogenic	chrX	63411540
AD39	MSS	7,59	APC	TRUNC	c.2413C>T	p.Arg805Ter	13%	Pathogenic	chr5	112173704
AD39	MSS	7,59	APC	TRUNC	c.4192_4193	p.Arg1399PhefsTer9	9%	Pathogenic	chr5	112175480
AD39	MSS	7,59	FBXW7	MISSENSE	c.1394G>A	p.Arg465His	8%	Likely pathogenic	chr4	153249384
AD39	MSS	7,59	TP53	MISSENSE	c.514G>T	p.Val172Phe	16%	Likely pathogenic	chr17	7578416
AD39	MSS	7,59	ARID1A	MISSENSE	c.226G>C	p.Gly76Arg	69%	VUS	chr1	27023120
AD39	MSS	7,59	MARCO	MISSENSE	c.1472C>T	p.Thr491Met	6%	VUS	chr2	119752005
AD39	MSS	7,59	HDAC9	MISSENSE	c.3074T>C	p.Leu1025Pro	53%	VUS	chr7	19015480
AD39	MSS	7,59	TCF7L2	TRUNC	c.1186C>T	p.Gln396Ter	15%	Likely pathogenic	chr10	114912116
AD39	MSS	7,59	KMT2D	MISSENSE	c.286G>A	p.Val96Met	52%	VUS	chr12	49448425

AD39	MSS	7,59	MTA1	MISSENSE	c.1172G>A	p.Arg391Gln	47%	VUS	chr14	105930464
AD39	MSS	7,59	AXIN2	MISSENSE	c.607G>A	p.Gly203Arg	7%	VUS	chr17	63554132
AD39	MSS	7,59	SMARCA4	MISSENSE	c.2089G>A	p.Asp697Asn	15%	VUS	chr19	11118665
AD39	MSS	7,59	CST5	MISSENSE	c.107C>T	p.Ala36Val	47%	VUS	chr20	23860207
AD39	MSS	7,59	USP9X	MISSENSE	c.4122C>G	p.Asp1374Glu	7%	VUS	chrX	41055880
AD40	MSS	11,34	NRAS	MISSENSE	c.37G>C	p.Gly13Arg	18%	Pathogenic	chr1	115258745
AD40	MSS	11,34	APC	TRUNC	c.832C>T	p.Gln278Ter	12%	Pathogenic	chr5	112137078
AD40	MSS	11,34	APC	TRUNC	c.3907C>T	p.Gln1303Ter	14%	Pathogenic	chr5	112175198
AD40	MSS	11,34	TP53	MISSENSE	c.725G>A	p.Cys242Tyr	27%	Pathogenic	chr17	7577556
AD40	MSS	11,34	FBXW7	MISSENSE	c.1513C>T	p.Arg505Cys	17%	Likely pathogenic	chr4	153247289
AD40	MSS	11,34	CDH10	MISSENSE	c.1360T>G	p.Trp454Gly	27%	VUS	chr5	24505254
AD40	MSS	11,34	CSMD3	MISSENSE	c.6820C>T	p.His2274Tyr	36%	VUS	chr8	113349793
AD40	MSS	11,34	PTCH1	TRUNC	c.755del	p.Pro252LeufsTer17	18%	Likely pathogenic	chr9	98242862
AD40	MSS	11,34	OS9	MISSENSE	c.1385G>A	p.Arg462Gln	48%	VUS	chr12	58112179
AD40	MSS	11,34	POLE	MISSENSE	c.1588G>A	p.Asp530Asn	49%	VUS	chr12	133249311
AD40	MSS	11,34	TSC2	MISSENSE	c.1119G>T	p.Gln373His	26%	VUS	chr16	2110814
AD40	MSS	11,34	ZFHX3	MISSENSE	c.8110G>A	p.Val2704Ile	45%	VUS	chr16	72828471
AD40	MSS	11,34	ZFHX3	MISSENSE	c.2548C>G	p.Leu850Val	4%	VUS	chr16	72991497
AD40	MSS	11,34	SETBP1	MISSENSE	c.1799C>T	p.Thr600Met	18%	VUS	chr18	42531104
AD40	MSS	11,34	SMAD2	MISSENSE	c.862G>A	p.Glu288Lys	22%	VUS	chr18	45374981
AD40	MSS	11,34	JAK3	MISSENSE	c.349C>T	p.Arg117Cys	19%	VUS	chr19	17954260
AD40	MSS	11,34	ERCC2	MISSENSE	c.170T>C	p.Met57Thr	6%	VUS	chr19	45872341
AD40	MSS	11,34	PATJ	MISSENSE	c.3853G>A	p.Asp1285Asn	52%	VUS	chr1	62456022
AD40	MSS	11,34	MARCO	MISSENSE	c.1466G>A	p.Arg489Gln	52%	VUS	chr2	119751999
AD41	MSS	10,39	KRAS	MISSENSE	c.35G>T	p.Gly12Val	11%	Pathogenic	chr12	25398284
AD41	MSS	10,39	ASXL1	TRUNC	c.2077C>T	p.Arg693Ter	10%	Pathogenic	chr20	31022592
AD41	MSS	10,39	APC	TRUNC	c.4464_4471	p.Leu1488PhefsTer25	14%	Pathogenic	chr5	112175755
AD41	MSS	10,39	APC	TRUNC	c.3100G>T	p.Glu1034Ter	11%	Pathogenic	chr5	112174391
AD41	MSS	10,39	CREBBP	TRUNC	c.2467C>T	p.Gln823Ter	9%	Likely pathogenic	chr16	3820984
AD41	MSS	10,39	SOX9	TRUNC	c.1005G>A	p.Trp335Ter	9%	Likely pathogenic	chr17	70120003
AD41	MSS	10,39	SOX9	TRUNC	c.1228C>T	p.Gln410Ter	18%	Likely pathogenic	chr17	70120226
AD41	MSS	10,39	BRCA1	MISSENSE	c.3020C>T	p.Ser1007Leu	9%	VUS	chr17	41244528
AD41	MSS	10,39	KLHL13	MISSENSE	c.530T>G	p.Leu177Arg	10%	VUS	chrX	117053533

AD41	MSS	10,39	MAGEA8	MISSENSE	c.653G>A	p.Arg218His	11%	VUS	chrX	149013699
AD41	MSS	10,39	EP300	SPLICE	c.1282+1G>A		11%	VUS	chr22	41526008
AD41	MSS	10,39	AXIN2	INFRAME	c.567_578de	p.Met189_Ser193deli	8%	VUS	chr17	63554161
AD41	MSS	10,39	PDE1A	MISSENSE	c.74G>C	p.Gly25Ala	11%	VUS	chr2	183387042
AD41	MSS	10,39	TGFBR2	MISSENSE	c.1493C>T	p.Pro498Leu	5%	VUS	chr3	30729897
AD41	MSS	10,39	OR2B2	MISSENSE	c.80C>T	p.Pro27Leu	45%	VUS	chr6	27880018
AD41	MSS	10,39	PLXDC2	MISSENSE	c.1549G>T	p.Val517Phe	52%	VUS	chr10	20568707
AD41	MSS	10,39	KMT2D	MISSENSE	c.5218G>A	p.Ala1740Thr	51%	VUS	chr12	49437752
AD41	MSS	10,39	MGA	MISSENSE	c.8316T>G	p.Asp2772Glu	50%	VUS	chr15	42058596
AD41	MSS	10,39	BCOR	MISSENSE	c.2531C>T	p.Pro844Leu	7%	VUS	chrX	39932068
AD42	L-MSI	10,51	KRAS	MISSENSE	c.34G>T	p.Gly12Cys	23%	Pathogenic	chr12	25398285
AD42	L-MSI	10,51	TP53	MISSENSE	c.517G>A	p.Val173Met	24%	Pathogenic	chr17	7578413
AD42	L-MSI	10,51	APC	TRUNC	c.4233del	p.Ser1411ArgfsTer4	28%	Likely pathogenic	chr5	112175524
AD42	L-MSI	10,51	SMAD4	TRUNC	c.766C>T	p.Gln256Ter	7%	Likely pathogenic	chr18	48584593
AD42	L-MSI	10,51	RAD50	MISSENSE	c.3508G>A	p.Asp1170Asn	51%	VUS	chr5	131973805
AD42	L-MSI	10,51	NOTCH4	MISSENSE	c.1153C>T	p.Arg385Cys	51%	VUS	chr6	32188188
AD42	L-MSI	10,51	PRDM1	MISSENSE	c.1100C>T	p.Ser367Phe	50%	VUS	chr6	106553135
AD42	L-MSI	10,51	RUNX1T1	MISSENSE	c.73T>C	p.Trp25Arg	5%	VUS	chr8	93107623
AD42	L-MSI	10,51	CSMD3	MISSENSE	c.9211A>G	p.Asn3071Asp	40%	VUS	chr8	113299413
AD42	L-MSI	10,51	JAK2	MISSENSE	c.171T>A	p.Phe57Leu	51%	VUS	chr9	5022158
AD42	L-MSI	10,51	NPY4R	MISSENSE	c.620T>C	p.Leu207Pro	25%	VUS	chr10	47087403
AD42	L-MSI	10,51	FGF4	MISSENSE	c.331A>T	p.Thr111Ser	50%	VUS	chr11	69589522
AD42	L-MSI	10,51	NTF3	MISSENSE	c.261_266de	p.Gly89Glu	46%	VUS	chr12	5603602
AD42	L-MSI	10,51	KMT2D	MISSENSE	c.15908G>A	p.Arg5303His	53%	VUS	chr12	49418606
AD42	L-MSI	10,51	ACVR1B	MISSENSE	c.1357C>T	p.Arg453Cys	24%	VUS	chr12	52385742
AD42	L-MSI	10,51	CD276	MISSENSE	c.290_291de	p.Pro97Leu	64%	VUS	chr15	73994806
AD42	L-MSI	10,51	IGF1R	MISSENSE	c.2524C>T	p.Pro842Ser	74%	VUS	chr15	99467143
AD42	L-MSI	10,51	KMT2B	MISSENSE	c.1033G>A	p.Gly345Arg	49%	VUS	chr19	36211282
AD42	L-MSI	10,51	ZNF544	TRUNC	c.2069_2072	p.Ser691ArgfsTer2	51%	VUS	chr19	58774042
AD42	L-MSI	10,51	THOC5	MISSENSE	c.1544T>C	p.Val515Ala	48%	VUS	chr22	29913301
AD42	L-MSI	10,51	EPHA2	MISSENSE	c.2344C>T	p.Arg782Cys	51%	VUS	chr1	16458347
AD42	L-MSI	10,51	NRAS	MISSENSE	c.187G>A	p.Glu63Lys	4%	VUS	chr1	115256524
AD42	L-MSI	10,51	DOCK3	MISSENSE	c.3278C>T	p.Pro1093Leu	21%	VUS	chr3	51352435

AD42	L-MSI	10,51	FHIT	MISSENSE	c.322C>T	p.His108Tyr	46%	VUS	chr3	59908098
AD42	L-MSI	10,51	FBXW7	TRUNC	c.1099C>T	p.Arg367Ter	46%	Likely pathogenic	chr4	153251907
AD42	L-MSI	10,51	RASA1	MISSENSE	c.482G>A	p.Gly161Glu	23%	VUS	chr5	86564750
AD43	MSS	10,47	PTPN11	MISSENSE	c.854T>C	p.Phe285Ser	7%	Pathogenic	chr12	112915455
AD43	MSS	10,47	TP53	MISSENSE	c.528C>G	p.Cys176Trp	30%	Likely pathogenic	chr17	7578402
AD43	MSS	10,47	APC	TRUNC	c.2353_2357	p.His785Ter	37%	Likely pathogenic	chr5	112173644
AD43	MSS	10,47	SOX9	TRUNC	c.1230del	p.Gln410HisfsTer60	63%	Likely pathogenic	chr17	70120228
AD43	MSS	10,47	PIK3CA	MISSENSE	c.3145G>C	p.Gly1049Arg	23%	Likely pathogenic	chr3	178952090
AD43	MSS	10,47	POLD1	TRUNC	c.1924_1928	p.Thr642GlyfsTer95	15%	VUS	chr19	50912410
AD43	MSS	10,47	ACVR2A	TRUNC	c.1322T>G	p.Leu441Ter	15%	VUS	chr2	148683705
AD43	MSS	10,47	ACVR2A	SPLICE	c.56-2A>C		31%	VUS	chr2	148653868
AD43	MSS	10,47	JAK1	TRUNC	c.1778C>T	p.Thr593Met	5%	VUS	chr1	65313336
AD43	MSS	10,47	PIK3CA	MISSENSE	c.3052G>C	p.Asp1018His	22%	VUS	chr3	178951997
AD43	MSS	10,47	FAT1	MISSENSE	c.9208T>A	p.Phe3070Ile	25%	VUS	chr4	187535366
AD43	MSS	10,47	SDHA	MISSENSE	c.1775A>G	p.His592Arg	25%	VUS	chr5	251564
AD43	MSS	10,47	CSMD3	MISSENSE	c.6461T>C	p.Val2154Ala	4%	VUS	chr8	113353897
AD43	MSS	10,47	PTCH1	MISSENSE	c.67G>A	p.Gly23Ser	53%	VUS	chr9	98279036
AD43	MSS	10,47	CACNB3	TRUNC	c.1336C>T	p.Leu446Phe	59%	VUS	chr12	49221602
AD43	MSS	10,47	LETMD1	MISSENSE	c.229C>G	p.Arg77Gly	44%	VUS	chr12	51442923
AD43	MSS	10,47	TTLL5	MISSENSE	c.3746C>T	p.Ser1249Phe	20%	VUS	chr14	76368490
AD43	MSS	10,47	CIC	MISSENSE	c.6556C>T	p.Arg2186Cys	40%	VUS	chr19	42797777
AD43	MSS	10,47	PDGFRA	TRUNC	c.1435C>T	p.Arg479Ter	9%	VUS	chr4	55139774
AD43	MSS	10,47	MYBL2	TRUNC	c.1237C>T	p.Gln413Ter	40%	VUS	chr20	42331415
AD44	MSS	3,79	BRAF	MISSENSE	c.1799T>A	p.Val600Glu	23%	Pathogenic	chr7	140453136
AD44	MSS	3,79	TP53	TRUNC	c.825_826de	p.Ala276LeufsTer29	20%	Likely pathogenic	chr17	7577114
AD44	MSS	3,79	FBXW7	MISSENSE	c.1394G>A	p.Arg465His	21%	Likely pathogenic	chr4	153249384
AD44	MSS	3,79	MYCN	MISSENSE	c.847G>A	p.Glu283Lys	23%	VUS	chr2	16085671
AD44	MSS	3,79	KIT	MISSENSE	c.1720A>G	p.Thr574Ala	50%	VUS	chr4	55593654
AD44	MSS	3,79	CHD4	MISSENSE	c.3314G>A	p.Arg1105Gln	20%	VUS	chr12	6700658
AD44	MSS	3,79	ZFHX3	MISSENSE	c.8110G>A	p.Val2704Ile	56%	VUS	chr16	72828471
AD44	MSS	3,79	KMT2B	MISSENSE	c.5363C>T	p.Pro1788Leu	49%	VUS	chr19	36221694
AD45	MSS	3,02	KRAS	MISSENSE	c.35G>A	p.Gly12Asp	28%	Pathogenic	chr12	25398284
AD45	MSS	3,02	AURKC	TRUNC	c.145del	p.Leu49TrpfsTer23	44%	Pathogenic	chr19	57743438

AD45	MSS	3,02	APC	TRUNC	c.2626C>T	p.Arg876Ter	28%	Pathogenic	chr5	112173917
AD45	MSS	3,02	APC	TRUNC	c.4289_4292	p.Thr1430SerfsTer42	25%	Pathogenic	chr5	112175580
AD45	MSS	3,02	TP53	TRUNC	c.851_852de	p.Thr284ArgfsTer21	37%	Likely pathogenic	chr12	4479706
AD45	MSS	3,02	FGF23	MISSENSE	c.559C>T	p.Arg187Trp	32%	VUS	chr17	7577086
AD45	MSS	3,02	EP300	SPLICE	c.1282+1G>A		36%	VUS	chr22	41526008
AD46	MSS	9,58	APC	TRUNC	c.3340C>T	p.Arg1114Ter	23%	Pathogenic	chr5	112174631
AD46	MSS	9,58	PTPN11	MISSENSE	c.1403C>T	p.Thr468Met	15%	Pathogenic	chr12	112926270
AD46	MSS	9,58	TP53	TRUNC	c.538G>T	p.Glu180Ter	65%	Pathogenic	chr17	7578392
AD46	MSS	9,58	APC	TRUNC	c.4058_4059	p.Glu1353ValfsTer21	31%	Likely pathogenic	chr5	112175349
AD46	MSS	9,58	INPP4B	MISSENSE	c.2587G>T	p.Asp863Tyr	22%	VUS	chr4	143003239
AD46	MSS	9,58	ADAMTS12	MISSENSE	c.2533C>T	p.Arg845Cys	22%	VUS	chr5	33596160
AD46	MSS	9,58	APC	MISSENSE	c.2584A>G	p.Asn862Asp	34%	VUS	chr5	112173875
AD46	MSS	9,58	CARD11	MISSENSE	c.868G>A	p.Gly290Arg	55%	VUS	chr7	2978462
AD46	MSS	9,58	JAK2	MISSENSE	c.1117T>C	p.Tyr373His	72%	VUS	chr9	5064943
AD46	MSS	9,58	TCF7L2	MISSENSE	c.1258C>T	p.Arg420Trp	29%	VUS	chr10	114912188
AD46	MSS	9,58	PTPN11	MISSENSE	c.226G>A	p.Glu76Lys	39%	VUS	chr12	112888210
AD46	MSS	9,58	POLE	MISSENSE	c.218A>G	p.Asp73Gly	27%	VUS	chr12	133257260
AD46	MSS	9,58	XRCC3	MISSENSE	c.242G>A	p.Arg81His	9%	VUS	chr14	104173504
AD46	MSS	9,58	NTRK3	MISSENSE	c.1751A>G	p.Asp584Gly	18%	VUS	chr15	88476381
AD46	MSS	9,58	ZFHX3	TRUNC	c.9589_9598	p.Gln3197SerfsTer41	41%	Likely pathogenic	chr16	72822578
AD46	MSS	9,58	FANCA	MISSENSE	c.2783C>T	p.Thr928Ile	20%	VUS	chr16	89828426
AD46	MSS	9,58	SMAD4	MISSENSE	c.1606C>A	p.Leu536Ile	33%	VUS	chr18	48604784
AD46	MSS	9,58	CIC	MISSENSE	c.6233G>A	p.Arg2078Gln	7%	VUS	chr19	42797150
AD46	MSS	9,58	AR	MISSENSE	c.2661G>A	p.Met887Ile	6%	VUS	chrX	66943581
AD46	MSS	9,58	ZMYM3	MISSENSE	c.3148C>T	p.Arg1050Trp	5%	VUS	chrX	70464284
AD47	MSS	4,82	APC	TRUNC	c.4316del	p.Pro1439LeufsTer34	27%	Pathogenic	chr5	112175606
AD47	MSS	4,82	TP53	MISSENSE	c.733G>A	p.Gly245Ser	57%	Pathogenic	chr17	7577548
AD47	MSS	4,82	APC	TRUNC	c.2805_2816	p.Tyr935Ter	13%	Likely pathogenic	chr5	112174096
AD47	MSS	4,82	ERBB4	MISSENSE	c.2398C>T	p.Pro800Ser	18%	VUS	chr2	212426717
AD47	MSS	4,82	TBC1D5	MISSENSE	c.2147G>C	p.Gly716Ala	79%	VUS	chr3	17208272
AD47	MSS	4,82	FAS	MISSENSE	c.317T>C	p.Leu106Ser	83%	VUS	chr10	90767577
AD47	MSS	4,82	LARGE2	MISSENSE	c.403G>A	p.Ala135Thr	46%	VUS	chr11	45945739
AD47	MSS	4,82	LNX2	MISSENSE	c.541T>G	p.Leu181Val	42%	VUS	chr13	28143280

AD47	MSS	4,82	CDK12	MISSENSE	c.778G>A	p.Asp260Asn	9%	VUS	chr17	37619102
AD48	MSS	1,9	KRAS	mISSENSE	c.180_181de	p.Gln61Lys	19%	Pathogenic	chr12	25380277
AD48	MSS	1,9	TP53	MISSENSE	c.817C>T	p.Arg273Cys	28%	Pathogenic	chr17	7577121
AD48	MSS	1,9	CSMD1	MISSENSE	c.4892G>A	p.Gly1631Glu	57%	VUS	chr8	3063118
AD48	MSS	1,9	CSMD3	MISSENSE	c.3289G>A	p.Val1097Ile	10%	VUS	chr8	113657359
AD48	MSS	1,9	GDPD4	MISSENSE	c.680A>C	p.His227Pro	39%	VUS	chr11	76979529
AD48	MSS	1,9	COG1	MISSENSE	c.1187A>G	p.Asn396Ser	32%	VUS	chr17	71196821
AD49	MSS	nv	APC	TRUNC	c.646C>T	p.Arg216Ter	28%	Pathogenic	chr5	112128143
AD49	MSS	nv	KRAS	MISSENSE	c.35G>A	p.Gly12Asp	35%	Pathogenic	chr12	25398284
AD49	MSS	nv	TP53	TRUNC	c.586C>T	p.Arg196Ter	70%	Pathogenic	chr17	7578263
AD49	MSS	nv	CTNNB1	MISSENSE	c.1161T>G	p.Asn387Lys	23%	Likely pathogenic	chr3	41274911
AD49	MSS	nv	LRP4	MISSENSE	c.3232A>G	p.Ile1078Val	62%	VUS	chr11	46898795
AD49	MSS	nv	ATM	MISSENSE	c.7475T>G	p.Leu2492Arg	74%	VUS	chr11	108201108
AD49	MSS	nv	CD276	MISSENSE	c.932C>T	p.Thr311Met	15%	VUS	chr15	73996198
AD49	MSS	nv	PFAS	MISSENSE	c.1085T>G	p.Leu362Arg	73%	VUS	chr17	8161134
AD49	MSS	nv	NCOR1	MISSENSE	c.6548G>A	p.Arg2183His	26%	VUS	chr17	15950396
AD49	MSS	nv	NCOR1	MISSENSE	c.6217C>T	p.Pro2073Ser	44%	VUS	chr17	15961003
AD49	MSS	nv	NOTCH3	MISSENSE	c.6103G>A	p.Gly2035Ser	58%	VUS	chr19	15272336
AD50	MSS	3,83	APC	TRUNC	c.2547_2550	p.Asp849GlufsTer11	20%	Pathogenic	chr5	112173835
AD50	MSS	3,83	APC	TRUNC	c.4476del	p.Thr1493ArgfsTer14	19%	Pathogenic	chr5	112175766
AD50	MSS	3,83	KRAS	MISSENSE	c.35G>T	p.Gly12Val	21%	Pathogenic	chr12	25398284
AD50	MSS	3,83	TP53	MISSENSE	c.747G>T	p.Arg249Ser	25%	Likely pathogenic	chr17	7577534
AD50	MSS	3,83	ADGRL3	MISSENSE	c.875G>A	p.Arg292His	12%	VUS	chr4	62598748
AD50	MSS	3,83	APC	MISSENSE	c.8182G>A	p.Val2728Met	52%	VUS	chr5	112179473
AD50	MSS	3,83	MET	MISSENSE	c.4061G>A	p.Arg1354Gln	52%	VUS	chr7	116436012
AD50	MSS	3,83	BRINP1	MISSENSE	c.1413C>A	p.Ser471Arg	17%	VUS	chr9	121930235
AD50	MSS	3,83	PTEN	MISSENSE	c.95T>G	p.Ile32Ser	23%	VUS	chr10	89653797
AD50	MSS	3,83	PARP4	MISSENSE	c.607_608de	p.Ala203Phe	30%	VUS	chr13	25068844
AD50	MSS	3,83	OR4M2	MISSENSE	c.13A>G	p.Asn5Asp	18%	VUS	chr15	22368588
AD50	MSS	3,83	RBMXL3	MISSENSE	c.2825G>A	p.Arg942His	28%	VUS	chrX	114426829
AD51	MSS	7,59	APC	TRUNC	c.646C>T	p.Arg216Ter	10%	Pathogenic	chr5	112128143
AD51	MSS	7,59	PIK3CA	MISSENSE	c.1633G>A	p.Glu545Lys	12%	Pathogenic	chr3	178936091
AD51	MSS	7,59	KRAS	MISSENSE	c.35G>T	p.Gly12Val	16%	Pathogenic	chr12	25398284

AD51	MSS	7,59	APC	TRUNC	c.3856G>T	p.Glu1286Ter	6%	Likely pathogenic	chr5	112175147
AD51	MSS	7,59	NKAPL	MISSENSE	c.283C>T	p.Arg95Cys	15%	VUS	chr6	28227432
AD51	MSS	7,59	BRINP3	TRUNC	c.1236del	p.Asn413ThrfsTer4	7%	VUS	chr1	190068213
AD51	MSS	7,59	FYN	MISSENSE	c.1600G>A	p.Gly534Ser	47%	VUS	chr6	111982956
AD51	MSS	7,59	ZNF479	MISSENSE	c.1097C>T	p.Ser366Leu	15%	VUS	chr7	57188025
AD51	MSS	7,59	BRCA2	MISSENSE	c.94T>C	p.Phe32Leu	48%	VUS	chr13	32893240
AD52	MSS	4,73	PIK3CA	MISSENSE	c.1634A>G	p.Glu545Gly	14%	Pathogenic	chr3	178936092
AD52	MSS	4,73	APC	TRUNC	c.694C>T	p.Arg232Ter	27%	Pathogenic	chr5	112128191
AD52	MSS	4,73	APC	TRUNC	c.4099C>T	p.Gln1367Ter	13%	Pathogenic	chr5	112175390
AD52	MSS	4,73	KRAS	MISSENSE	c.35G>T	p.Gly12Val	39%	Pathogenic	chr12	25398284
AD52	MSS	4,73	PLCG1	MISSENSE	c.436C>A	p.Gln146Lys	26%	VUS	chr17	5280473
AD52	MSS	4,73	RABEP1	MISSENSE	c.2088C>A	p.Asp696Glu	48%	VUS	chrY	14832620
AD52	MSS	4,73	NOTCH2	MISSENSE	c.230G>A	p.Gly77Glu	17%	VUS	chr1	120548137
AD53	L-MSI	6,66	APC	TRUNC	c.646C>T	p.Arg216Ter	7%	Pathogenic	chr5	112128143
AD53	L-MSI	6,66	KRAS	mISSENSE	c.35G>A	p.Gly12Asp	10%	Pathogenic	chr12	25398284
AD53	L-MSI	6,66	PIK3CA	mISSENSE	c.3140A>G	p.His1047Arg	14%	Pathogenic	chr3	178952085
AD53	L-MSI	6,66	APC	TRUNC	c.694C>T	p.Arg232Ter	16%	Pathogenic	chr5	112128191
AD53	L-MSI	6,66	SMAD4	MISSENSE	c.1157G>A	p.Gly386Asp	30%	Pathogenic	chr18	48593406
AD53	L-MSI	6,66	NCOR1	MISSENSE	c.6139C>T	p.Arg2047Trp	3%	VUS	chr17	15961250
AD53	L-MSI	6,66	FGF23	MISSENSE	c.722C>T	p.Pro241Leu	9%	VUS	chr12	4479543
AD53	L-MSI	6,66	CREBBP	MISSENSE	c.7222A>G	p.Met2408Val	38%	VUS	chr16	3777826
AD53	L-MSI	6,66	AHR	MISSENSE	c.1644T>G	p.Ile548Met	52%	VUS	chr7	17379093
AD53	L-MSI	6,66	BRCA1	MISSENSE	c.1363A>G	p.Asn455Asp	48%	VUS	chr17	41246185
AD53	L-MSI	6,66	SOX9	TRUNC	c.817dup	p.Val273GlyfsTer23	13%	Likely pathogenic	chr17	70119815
AD53	L-MSI	6,66	BCOR	MISSENSE	c.1789_1791	p.His597Tyr	4%	VUS	chrX	39932808
AD54	MSS	5,7	APC	TRUNC	c.4348C>T	p.Arg1450Ter	38%	Pathogenic	chr5	112175639
AD54	MSS	5,7	KRAS	MISSENSE	c.35G>A	p.Gly12Asp	28%	Pathogenic	chr12	25398284
AD54	MSS	5,7	TP53	MISSENSE	c.743G>A	p.Arg248Gln	4%	Pathogenic	chr17	7577538
AD54	MSS	5,7	AMER1	TRUNC	c.1489C>T	p.Arg497Ter	30%	Pathogenic	chrX	63411678
AD54	MSS	5,7	SOX9	TRUNC	c.788_789in	p.Arg264GlnfsTer32	23%	Likely pathogenic	chr17	70119780
AD54	MSS	5,7	FANCD2	TRUNC	c.479_480in	p.Tyr160Ter	31%	Likely pathogenic	chr3	10078012
AD54	MSS	5,7	MUTYH	MISSENSE	c.571A>T	p.Thr191Ser	48%	VUS	chr1	45798281
AD54	MSS	5,7	SPRR4	MISSENSE	c.202C>T	p.Pro68Ser	30%	VUS	chr1	152944568

AD54	MSS	5,7	NTRK1	MISSENSE	c.1021G>A	p.Val341Met	28%	VUS	chr1	156843595
AD54	MSS	5,7	CTNNB1	MISSENSE	c.1000G>A	p.Glu334Lys	3%	VUS	chr3	41268762
AD54	MSS	5,7	INPP4B	MISSENSE	c.586C>A	p.His196Asn	4%	VUS	chr4	143191845
AD54	MSS	5,7	APC	INFRAME	c.1598_160(	p.Leu533_Val2843deli	28%	VUS	chr5	112163675
AD54	MSS	5,7	TNFAIP3	MISSENSE	c.851A>T	p.Glu284Val	4%	VUS	chr6	138198258
AD54	MSS	5,7	LATS1	MISSENSE	c.2210G>A	p.Arg737Gln	14%	VUS	chr6	150001394
AD54	MSS	5,7	POM121L1	MISSENSE	c.134C>A	p.Ser45Tyr	30%	VUS	chr7	53103498
AD54	MSS	5,7	KMT2C	MISSENSE	c.11142A>C	p.Lys3714Asn	33%	VUS	chr7	151859520
AD54	MSS	5,7	CSMD3	MISSENSE	c.6490T>G	p.Tyr2164Asp	30%	VUS	chr8	113353868
AD54	MSS	5,7	ATM	MISSENSE	c.6860G>A	p.Gly2287Glu	45%	VUS	chr11	108196837
AD54	MSS	5,7	KMT2D	MISSENSE	c.12556C>G	p.Pro4186Ala	3%	VUS	chr12	49425932
AD54	MSS	5,7	RAD51	MISSENSE	c.140A>G	p.His47Arg	52%	VUS	chr15	40993314
AD54	MSS	5,7	SLX4	MISSENSE	c.1152_115(	p.Met386Val	41%	VUS	chr16	3650987
AD54	MSS	5,7	NF1	MISSENSE	c.3574G>A	p.Glu1192Lys	3%	VUS	chr17	29560097
AD55	MSS	11,35	APC	TRUNC	c.1286del	p.Pro429GlnfsTer25	21%	Pathogenic	chr5	112155014
AD55	MSS	11,35	APC	TRUNC	c.994C>T	p.Arg332Ter	23%	Pathogenic	chr5	112154723
AD55	MSS	11,35	TP53	TRUNC	c.916C>T	p.Arg306Ter	23%	Pathogenic	chr17	7577022
AD55	MSS	11,35	CYLD	TRUNC	c.340A>T	p.Lys114Ter	6%	Likely pathogenic	chr16	50783949
AD55	MSS	11,35	POLE	INFRAME	c.3672_367(	p.Ala1225dup	47%	VUS	chr12	133226384
AD55	MSS	11,35	SPEN	MISSENSE	c.10363C>G	p.Gln3455Glu	6%	VUS	chr1	16263994
AD55	MSS	11,35	MYCN	MISSENSE	c.1031C>T	p.Ala344Val	70%	VUS	chr2	16085855
AD55	MSS	11,35	PMS1	MISSENSE	c.287C>G	p.Ala96Gly	53%	VUS	chr2	190660649
AD55	MSS	11,35	CASP8	MISSENSE	c.203G>A	p.Arg68Gln	23%	VUS	chr2	202131412
AD55	MSS	11,35	SLF1	MISSENSE	c.795C>A	p.His265Gln	46%	VUS	chr5	93987463
AD55	MSS	11,35	CPSF1	MISSENSE	c.1682C>T	p.Thr561Ile	33%	VUS	chr8	145623985
AD55	MSS	11,35	PCDH15	MISSENSE	c.58C>A	p.Leu20Ile	22%	VUS	chr10	56423965
AD55	MSS	11,35	MADD	MISSENSE	c.2265G>T	p.Gln755His	56%	VUS	chr11	47306599
AD55	MSS	11,35	CREBBP	MISSENSE	c.1603G>A	p.Gly535Arg	9%	VUS	chr16	3831278
AD55	MSS	11,35	CYLD	MISSENSE	c.2483C>T	p.Pro828Leu	51%	VUS	chr16	50828136
AD55	MSS	11,35	DMD	MISSENSE	c.7036C>A	p.Pro2346Thr	11%	VUS	chrX	31893367
AD55	MSS	11,35	PIK3R1	SPLICE	c.1746-2A>T		20%	VUS	chr5	67591246
AD56	MSS	4,82	KRAS	MISSENSE	c.38G>A	p.Gly13Asp	28%	Pathogenic	chr12	25398281
AD56	MSS	4,82	APC	TRUNC	c.646C>T	p.Arg216Ter	26%	Pathogenic	chr5	112128143

AD56	MSS	4,82	APC	TRUNC	c.4199C>A	p.Ser1400Ter	28%	Pathogenic	chr5	112175490
AD56	MSS	4,82	TP53	TRUNC	c.818G>A	p.Arg273His	42%	Pathogenic	chr17	7577120
AD56	MSS	4,82	PARP1	TRUNC	c.1600_1612	p.Asp534ThrfsTer16	21%	Likely pathogenic	chr1	226567261
AD56	MSS	4,82	TMEM132I	MISSENSE	c.1954G>A	p.Ala652Thr	25%	VUS	chr12	129563240
AD56	MSS	4,82	TSC2	MISSENSE	c.2395C>T	p.Arg799Cys	32%	VUS	chr16	2124240
AD56	MSS	4,82	TNP2	MISSENSE	c.281G>A	p.Arg94Gln	45%	VUS	chr16	11362839
AD56	MSS	4,82	ZFHX3	MISSENSE	c.9093C>G	p.Ile3031Met	45%	VUS	chr16	72827488
AD56	MSS	4,82	COG1	MISSENSE	c.1229C>T	p.Pro410Leu	13%	VUS	chr17	71196863
AD56	MSS	4,82	IQCA1L	MISSENSE	c.277C>T	p.Arg93Cys	4%	VUS	chr7	150901657
